# Short-Term Incubation of H9c2 Cardiomyocytes with Cannabigerol Attenuates Diacylglycerol Accumulation in Lipid Overload Conditions

**DOI:** 10.3390/cells14130998

**Published:** 2025-06-30

**Authors:** Sylwia Dziemitko, Adrian Chabowski, Ewa Harasim-Symbor

**Affiliations:** Department of Physiology, Medical University of Bialystok, Mickiewicz Str. 2C, 15-222 Bialystok, Poland

**Keywords:** CBG, DAG, fatty acid transporters, H9c2 cardiomyocytes, lipids

## Abstract

Fatty acids (FAs) play a crucial role in human physiology, including energy production and serving as signaling molecules. However, a dysregulation in their balance can lead to multiple disorders, such as obesity and metabolic syndrome. These pathological conditions alter the balance between the heart’s energetic substrates, promoting an increased reliance on FAs and decreased cardiac efficiency. A therapeutic application of a non-psychotropic phytocannabinoid, cannabigerol (CBG), seems to be a promising target since it interacts with different receptors and ion channels, including cannabinoid receptors—CB_1_ and CB_2_, α2 adrenoceptor, or 5-hydroxytryptamine receptor. Therefore, in the current study, we evaluated a concentration-dependent effect of CBG (2.5 µM, 5 µM, and 10 µM) on H9c2 cardiomyocytes in lipid overload conditions. Gas–liquid chromatography and Western blotting techniques were used to determine the cellular lipid content and the level of selected proteins involved in FA metabolism, glucose transport, and the insulin signaling pathway. The glucose uptake assay was performed using a colorimetric method. Eighteen-hour CBG treatment in the highest concentration (10 µM) significantly diminished the accumulation of diacylglycerols (DAGs) and the saturation status of this lipid fraction. Moreover, the same concentration of CBG markedly decreased the level of FA transporters, namely fatty acid translocase (CD36) and plasma membrane fatty acid-binding protein (FABPpm), in the presence of palmitate (PA) in the culture medium. The results of our experiment suggest that CBG can significantly modulate lipid storage and composition in cardiomyocytes, thereby protecting against lipid-induced cellular dysfunction.

## 1. Introduction

Fatty acids (FAs), apart from being an important dietary energy source for humans, also serve as components of cell membranes and biologically active compounds influencing various metabolic and signaling pathways [1]. An imbalance between the amount of FAs consumed and the amount of FAs expended on energy production generates negative consequences, such as obesity, where an excess of FAs accumulates not only in the adipose tissue but also in other tissues and organs such as the liver and heart [2,3]. Consequently, obesity is often associated with hyperglycemia, dyslipidemia, insulin resistance, and hypertension, which are referred to as metabolic syndrome [4].

Physiologically, the heart exhibits high energy demands compared to other organs, due to maintaining continuous function as a pump. More than 95% of the produced ATP originates from oxidative phosphorylation in the mitochondria, and less than 5% comes from glycolysis and the citric acid cycle [5]. The main myocardial energy source is FA oxidation, constituting approximately 60% of all produced energy. The remaining 40% is covered by carbohydrates (approximately 30% by glucose and 10% by lactate) [6]. The presence of membrane-associated proteins known as glucose transporter types 1 and 4 (GLUT-1 and GLUT-4) and FA transporters (fatty acid translocase—CD36/SR-B2 and plasma membrane fatty acid-binding protein—FABPpm as well as fatty acid transport proteins—FATPs; FATP-1, -4, -6) determines the degree of glucose and FAs utilization by the heart [6,7], respectively. Upon entering the cell, FAs can be utilized or stored in the cytoplasm as triacylglycerols (TAGs). In healthy individuals, the intramyocardial TAG pool is maintained at low levels, with the majority of the FAs being converted into long-chain acyl-coenzyme A (CoA) esters by fatty acyl-CoA synthetase and directed to mitochondria for β-oxidation [8]. In parallel, GLUT-1 facilitates glucose transport independently of insulin, while GLUT-4 is responsible for insulin-sensitive glucose uptake. When insulin binds to its receptor on the cardiomyocytes’ surface, the receptor undergoes a conformational change and autophosphorylation, leading to the recruitment and phosphorylation of insulin receptor substrate (IRS). This in turn activates phosphatidylinositide-3 kinase (PI3K), protein kinase B (PKB/Akt), and Akt substrate of 160 kDa (AS160), resulting in the translocation of intracellular depots of GLUT-4 transporters to the plasma membrane and thereby increasing glucose uptake. Additionally, insulin enhances glycogen synthesis by activating glycogen synthase (GS) and inhibiting glycogen synthase kinase 3 (GSK-3) [9]. Once inside the cell, glucose is phosphorylated by hexokinase to form glucose-6-phosphate, which undergoes glycolysis, ultimately producing pyruvate, which is then oxidized in the mitochondria [10]. The above-mentioned proportion of preferred energy substrate (FA—60%; glucose—30%) utilization is altered in pathological states such as obesity, where an increased FA availability leads to elevated cardiac reliance on FAs. This results in lessened glucose oxidation and glycolysis as well as decreased heart efficiency [11].

The endocannabinoid system (ECS) is a complex endogenous signaling system influencing multiple physiological processes, including various metabolic routes, feeding, emotional behavior, pain sensation, and fertility [12,13]. The ECS consists of endogenous cannabinoids (endocannabinoids; eCBs), their receptors, and the proteins that are involved in eCBs transport, synthesis, and degradation. Due to the ECS complexity, it is not an isolated system, but it rather influences plenty of different signaling pathways. For that reason, the term ‘endocannabinoidome’ was introduced to cover a wider endocannabinoid-related network affected by the modulation of ECS components [14]. So far, more than 550 chemical compounds and more than 100 phytocannabinoids (pCBs) have been identified in the Cannabis plant. The pCBs display their actions by binding to various receptors within the ECS. Cannabidiol (CBD) and Δ^9^-tetrahydrocannabinol (Δ^9^-THC) are the two most widely known and extensively studied pCBs. However, researchers are now focusing more frequently on some lesser known pCBs, such as cannabigerol (CBG) [15,16]. In contrast to Δ^9^-THC, CBG does not possess psychotropic activity. This compound is often defined as the ‘mother of all cannabinoids’ as it serves as a precursor for other pCBs. CBG binds to cannabinoid receptors, namely CB1 and CB2, acting as a weak/partial agonist [17,18], as well as influencing six transient receptor potential cation channels (TRPA1, TRPV1, TRPV2, TRPV3, TRPV4, and TRPM8) [19]. CBG also exhibits unique properties, so far unidentified in other pCBs, including the modulation of α2 adrenoceptor (agonist) and 5-hydroxytryptamine receptor (5HT1A; antagonist) [20]. A wide spectrum of biological activity has been identified up to this point, such as anti-inflammatory, neuromodulatory, or antibacterial effects [21,22].

Taking into account the above, researchers are constantly searching for new therapies and substances, especially plant-derived ones, serving as metabolic modulators that can restore physiological energetic balance in the cells, alleviate the symptoms of metabolic disorders, or protect against the development of obesity-related diseases. To date, no studies have shown the involvement of CBG in the regulation of lipid metabolism and energetic homeostasis in terms of FA transport and their cellular turnover in the heart in obesity, which indicates the existence of a gap in the knowledge regarding this pCB. In light of the above, the study’s major aim was to uncover whether CBG is a potential regulator of FA transport and lipid accumulation in the heart during the development of obesity. Therefore, we conducted our experiments on the H9c2 cell line in lipid overload conditions and different concentrations of CBG, where we evaluated the content and composition of selected lipid fractions, the level of proteins involved in FA transport and metabolism, the insulin signaling pathway, and glucose uptake.

## 2. Materials and Methods

### 2.1. Cell Culture

H9c2 rat cardiomyoblast cell line was purchased from American Type Culture Collection (cat. No. CRL1446TM, ATCC, Manassas, VA, USA). The cells were cultured in Dulbecco’s modified Eagle medium (DMEM) (Pan Biotech, Aidenbach, Germany) supplemented with 10% fetal bovine serum (FBS) (Pan Biotech, Aidenbach, Germany), 1% penicillin/streptomycin, 1% nonessential amino acids solution (Thermo Scientific, Rockford, IL, USA), and 2.5% HEPES (Thermo Scientific, Rockford, IL, USA) in humidified air (CO_2_ 5%) at 37 °C. After reaching a high degree of confluence, the medium was replaced with DMEM with 1% FBS, 1% penicillin/streptomycin, 1% non-essential amino acids solution, 2.5% HEPES, and 1μM retinoic acid (RA) for 5 days to induce differentiation.

### 2.2. Cell Treatment

Differentiated cardiac cells were then exposed to 300 µM palmitate (PA; cat. No. P9767, Sigma–Aldrich, St. Louis, MO, USA) and 2.5 µM, 5 µM, and 10 µM concentrations of CBG (cat. No. 15293; Cayman Chemical, Ann Arbor, MI, USA) for 18 h. The CBG concentrations were selected based on the available literature [23,24,25]. CBG was dissolved in dimethyl sulfoxide (DMSO) as the vehicle for treatment. The control groups in the experiment consisted of vehicle-only treatment and were subjected to the same experimental procedures and conditions as the experimental groups. The above concentration of PA and the time of incubation were established in the preliminary studies using a gas–liquid chromatography (GLC) method to measure lipid accumulation (Figure S2), and the cells’ viability was assessed with the use of an automated cell counter and trypan blue staining. It was determined that an 18 h incubation period was optimal for cardiomyocytes, ensuring high cell viability (cell viability was 70–80% in all experimental groups) and FA accumulation in contrast to a 24 h incubation time where a marked increase in the apoptosis of cardiomyocytes was observed (cell viability was 30–50% in all experimental groups). The PA solution was prepared by conjugating FAs with FA-free and endotoxin-free bovine serum albumin (BSA; cat. No. A8806; Sigma-Aldrich, St. Louis, MO, USA) as previously described [26]. In brief, to prepare the PA stock solution, PA was dissolved in a mixture of absolute ethanol and 1 M NaOH. The solution was then heated to 70 °C and conjugated with 10% BSA. Finally, the PA–BSA solution was diluted to the required concentration in media for cell treatment. The next stage of the experiment included the serum-starvation of the cardiomyocytes in DMEM (w/o glucose) for 4 h, which was followed by 10 min incubation with 100 nM insulin. The time used for the treatment with insulin was based on preliminary studies, where a 10–45 min time frame was examined (Figure S1). Based on the obtained results, the 10 min time point was selected as the most representative for our experimental conditions, consistent with findings reported in the literature [27,28].

### 2.3. Lipid Analysis

The Folch method [29] was used to extract lipids from H9c2 cells. The lysates were mixed with the solution of methanol, butylated hydroxytoluene (antioxidant), and chloroform, along with heptadecanoic acid as an internal standard. Using thin-layer chromatography (TLC), the desired lipid fractions, i.e., free fatty acid (FFA), diacylglycerol (DAG), TAG, and phospholipid (PL) were separated on silica gel plates (Silica Plate 60, 0.25 mm; Merck, Darmstadt, Germany). A resolving solution of heptane/isopropyl ether/acetic acid (60:40:3, *v*/*v*/*v*) was used for the separation. The desired lipid fractions were visualized on the dried plates under ultraviolet light. Subsequently, bands corresponding to the selected lipid fractions were collected and then eluted in appropriate solutions, i.e., chloroform/methanol (9:1, *v*/*v*) for DAG fraction, diethyl ether/hexane (1:1, *v*/*v*) for TAG fraction, and chloroform/methanol/water (5:5:1, *v*/*v*/*v*) for PL fraction. Next, in case of FFA and DAG fractions, transmethylation procedure was conducted by incubating the samples in a boron trifluoride-methanol solution for 10 min at 100 °C. In parallel to the samples containing TAG or PL fractions, diethyl ether solution and methyl acetate were added. The test tubes were mixed gently, and then, under these conditions, 1 M sodium methoxide in methanol was added for the methylation of the samples (10 min incubation at room temperature). The reaction was stopped by adding a saturated solution of oxalic acid, and the mixture was evaporated under a stream of nitrogen gas. After the methylation step, pentane was added to extract fatty acid methyl esters, and the samples were again evaporated. Samples were dissolved in hexane and analyzed. The identification and quantification of individual fatty acid methyl esters within each fraction were based on retention times and standard curves, respectively, using GLC (Hewlett-Packard 5890 Series II gas chromatograph, HP-INNOWax capillary column; Agilent Technologies, Santa Clara, CA, USA). The following individual FAs were evaluated: myristic acid (C14:0), palmitic acid (C16:0), palmitoleic acid (C16:1), stearic acid (C18:0), oleic acid (C18:1), linoleic acid (C18:2), linolenic acid (C18:3), arachidic acid (C20:0), arachidonic acid (C20:4), eicosapentaenoic acid (C20:5), behenic acid (C22:0), docosahexaenoic acid (C22:6), and lignoceric acid (C24:0). The total quantities of FFA, DAG, TAG, and PL were determined by summing the respective FA species in each specific fraction, and the results were expressed in nanomoles per milligram of protein. Additionally, the content of saturated fatty acids (SFAs) was calculated as a sum of C14:0, C16:0, C18:0, C20:0, C22:0, and C24:0; the content of monounsaturated fatty acids (MUFAs) was estimated as a sum of C16:1, C18:1, and C24:1; and the content of polyunsaturated fatty acids (PUFAs) was assessed as a sum of C18:3, C20:5, C22:6, C18:2, and C20:4.

### 2.4. Western Blot

The Western blot method was used to detect protein levels in the total cell lysate. In brief, cells were lysed in a radioimmunoprecipitation assay buffer (RIPA) containing a cocktail of protease and phosphatase inhibitors (Roche Diagnostics GmbH, Mannheim, Germany) and sonicated for 30 sec at 4 °C. The total protein concentration was assessed using the bicinchoninic acid (BCA) method with BSA as a standard. Afterwards, Laemmli buffer was used to reconstitute the homogenates, which were subsequently loaded (10 µg of protein per lane) on Criterion TGX Stain-Free™ Precast Gels (Bio-Rad, Hercules, CA, USA) and separated during electrophoresis. The separated proteins were then transferred onto polyvinylidene fluoride (PVDF) (semi-dry transfer) or nitrocellulose membranes (wet transfer). Thereafter, membranes were blocked in 5% non-fat dry milk or BSA for 1 h and incubated overnight at 4 °C with the selected primary antibodies: CD36 (1:500, cat. No. sc-7309, Santa Cruz Biotechnology, Inc., Dallas, TX, USA), FABPpm (1:8000, cat. No. ab180162, Abcam, Cambridge, UK), FATP-1 (1:500, cat. No. sc-25541, Santa Cruz Biotechnology, Inc., Dallas, TX, USA), FATP-4 (1:500, cat. No. sc-5834, Santa Cruz Biotechnology, Inc., Dallas, TX, USA), IRS-1 (1:1000, cat. No. 2382, Cell Signaling Technology, Danvers, MA, USA), phosphorylated insulin receptor substrate 1 (pIRS1 [Ser307]; 1:1000, cat. No. 44–813 G, Thermo Scientific, Rockford, IL, USA), Akt/PKB(1:1000, cat. No. 4691, Cell Signaling Technology), phosphorylated protein kinase B (pAkt/PKB [Ser473]; 1:1000, cat. No. 4051, Cell Signaling Technology, Danvers, MA, USA), GSK-3 (1:500, cat. No. MA5-15109, Thermo Scientific, Rockford, IL, USA), phosphorylated glycogen synthase kinase 3 (pGSK-3 [Ser9]; 1:500, cat. No. MA5-14873, Thermo Scientific, Rockford, IL, USA), AS160 (1:500, cat. No. 2670, Cell Signaling Technology, Danvers, MA, USA), phosphorylated Akt substrate of 160 kDa (pAS160 [Thr642]; 1:1000, cat. No. 4288,Cell Signaling Technology, Danvers, MA, USA), GLUT-4 (1:500, cat. No. sc-53566, Santa Cruz Biotechnology, Inc., Dallas, TX, USA), adipose triglyceride lipase (ATGL; 1:1000, cat. No. sc-67355, Santa Cruz Biotechnology, Inc., Dallas, TX, USA), citrate synthase (CS; 1:500, cat. No. sc-390693, Santa Cruz Biotechnology, Inc., Dallas, TX, USA), carnitine palmitoyltransferase I (CPTI; 1:500, cat. No. sc-393070, Santa Cruz Biotechnology, Inc., Dallas, TX, USA), diacylglycerol O-acyltransferase 1 (DGAT1; 1:500, cat. No. NB110-41487, Novus Biologicals, LLC, Centennial, CO, USA), monoacylglycerol lipase (MAGL; 1:500, cat. No. sc-398942, Santa Cruz Biotechnology, Inc., Dallas, TX, USA), cytosolic phospholipase A_2_ (cPLA_2_; 1:200, cat. No. sc-454, Santa Cruz Biotechnology, Inc., Dallas, TX, USA), cyclooxygenase 1 (COX-1; 1:500, cat. No. sc-19998, Santa Cruz Biotechnology, Inc., Dallas, TX, USA) COX-2 (1:500, cat. No. sc-166475, Santa Cruz Biotechnology, Inc., Dallas, TX, USA), 5-lipoxygenase (5-LOX; 1:1000; ab169755, Abcam, Cambridge, UK), 12/15-LOX (1:500; cat. No. sc-133085, Santa Cruz Biotechnology, Inc., Dallas, TX, USA), fatty-acid amide hydrolase 1 (FAAH1; 1:200, cat. No. ab54615, Abcam, Cambridge, UK), cannabinoid receptor 1 (CB_1_; 1:200, cat. No. ab23703, Abcam, Cambridge, UK), and CB_2_ (1:200; cat. No. ab3561, Abcam, Cambridge, UK). A secondary antibody conjugated with horseradish peroxidase (HRP; 1:3000; Santa Cruz Biotechnology, Dallas, TX, USA) was then used to detect the above proteins. The protein bands were visualized by adding the appropriate substrate for HRP and using a ChemiDoc visualization system (Bio-Rad, Hercules, CA, USA). The level of the examined proteins was quantified using Bio-Rad’s Stain-Free™ gels technology and the total protein normalization method. This technique is based on the detection of the total protein content in each lane before and after transfer. This approach enables precise normalization without the need for housekeeping proteins [30]. The total levels of the aforementioned proteins were presented as a percentage, and the control group was set as 100%. All groups for each examined protein were analyzed on the same gels, ensuring that they were subjected to consistent experimental conditions.

### 2.5. Glucose Uptake

The glucose uptake assay was performed using the Glucose Uptake Colorimetric Assay Kit (Sigma-Aldrich, St. Louis, MO, USA). Before the experiment, cells were serum-starved for 40 min by planting them in Krebs–Ringer-Phosphate-HEPES (KRPH) buffer containing 2% BSA. Afterward, cardiomyocytes were stimulated in the presence or absence of insulin (100 nM, 10 min) [31] or 2-deoxyglucose (2-DG [10 nM, 20 min]), respectively. Following the incubation period, the cells were lyzed, freezed, and then heated to 85 °C. The next step involved the neutralization and subsequent dilution of the cells’ lysates. After incubations, the absorbance was measured at 412 nm using a Synergy H1 Hybrid reader for a microplate (BioTek, Instruments, Winooski, VT, USA).

### 2.6. Statistical Analysis

All data are reported as mean values ± SD or percentage of the control group based on six (GLC), three (Western blotting), or sixteen (glucose uptake) independent determinations. The distribution of values and homogeneity of variance were assessed using the Shapiro–Wilk test and Bartlett’s test, respectively. Statistical differences between groups were evaluated by performing two-way ANOVA followed by an appropriate post hoc test (Tukey’s test and *t*-test) using GraphPad Prism version 7.0 for Windows (GraphPad Software, La Jolla, CA, USA). For all data, *p*-value < 0.05 was considered statistically significant.

## 3. Results

### 3.1. The Influence of 18 h PA and/or CBG Treatment on the Content of FFA, DAG, TAG, and PL Fractions in H9c2 Cardiomyocytes

**Figure 1:**
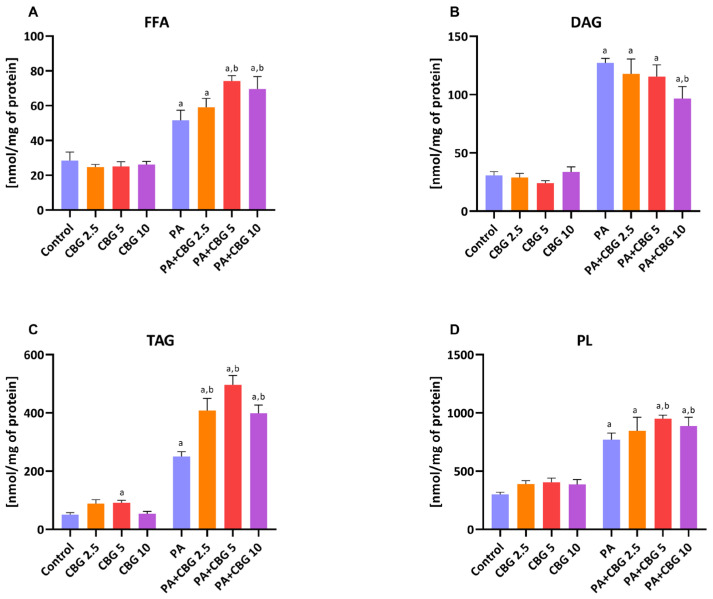
The content of free fatty acid—FFA (**A**), diacyloglycerol—DAG (**B**), triacyloglycerol—TAG (**C**), and phospholipid—PL (**D**), fractions in H9c2 cardiomyocytes after 18 h incubation with palmitate (PA; 300 μM) and/or cannabigerol (CBG; 2.5 μM, 5 μM, 10 μM). The data are expressed in nmol/mg of protein as mean values ± SD and are based on six independent determinations in each group. ^a^ *p* < 0.05 indicates a significant difference between the control group and the examined group; ^b^ *p* < 0.05 indicates a significant difference between the PA group and the examined group.

To assess the influence of CBG on the accumulation of lipids in H9c2 cardiomyocytes, we examined the contents of different lipid pools, i.e., FFA, DAG, TAG, and PL, in control and lipid overload conditions. Data in Figure 1A–D showed that the contents of all examined fractions, namely FFA, DAG, TAG, and PL, were considerably increased after incubation with PA (+80.42%, +312.47%, +396.07%, and +156.59%, *p* < 0.05 vs. the control group, respectively). Additionally, in each CBG and PA group, the levels of FFA (PA + CBG 2.5 µM: +106.89%, PA + CBG 5 µM: +159.33%, PA + CBG 10 µM: +155.42%, *p* < 0.05; Figure 1A), DAG (PA + CBG 2.5 µM: +281.91%, PA + CBG 5 µM: +274.34%, PA + CBG 10 µM: +213.32, *p* < 0.05; Figure 1B), TAG (PA + CBG 2.5 µM: +709.78, PA + CBG 5 µM: +885.75%, PA + CBG 10 µM: +690.90%, *p* < 0.05; Figure 1C), and PL (PA + CBG 2.5 µM: +181.62%, PA + CBG 5 µM: +216.06%, PA + CBG 10 µM: +195.75%, *p* < 0.05; Figure 1D) were elevated after PA treatment compared to the control group. The incubation of H9c2 cardiomyocytes with CBG in the concentration of 2.5µM in the lipid overload conditions significantly heightened only the TAG content (+63.24%, *p* < 0.05; Figure 1C), whereas higher CBG concentrations, namely 5 µM and 10 µM, raised the content of FFA (PA + CBG 5 µM:+43.73%, PA + CBG 10 µM: +41.57%, *p* < 0.05 vs. the PA group; Figure 1A), TAG (PA + CBG 5 µM:+98.71%, PA + CBG 10 µM: +59.43%, *p* < 0.05 vs. the PA group; Figure 1C), and PL (PA + CBG 5 µM:+23.18%, PA + CBG 10 µM: +15.26%, *p* < 0.05 vs. the PA group; Figure 1D) fractions. Moreover, the highest concentration of CBG, 10 µM, was found to markedly diminish the amount of DAG pool (−24.04%, *p* < 0.05 vs. the PA group; Figure 1B) in lipid overload conditions, while the concentration of 5 µM increased the level of TAG pool (+82.07%, *p* < 0.05 vs. the control group; Figure 1C).

### 3.2. The Influence of 18 h PA and/or CBG Treatment on the Level of Fatty Acid Transporters, Including CD36, FABPpm, FATP 1, and FATP 4 in H9c2 Cardiomyocytes

**Figure 2:**
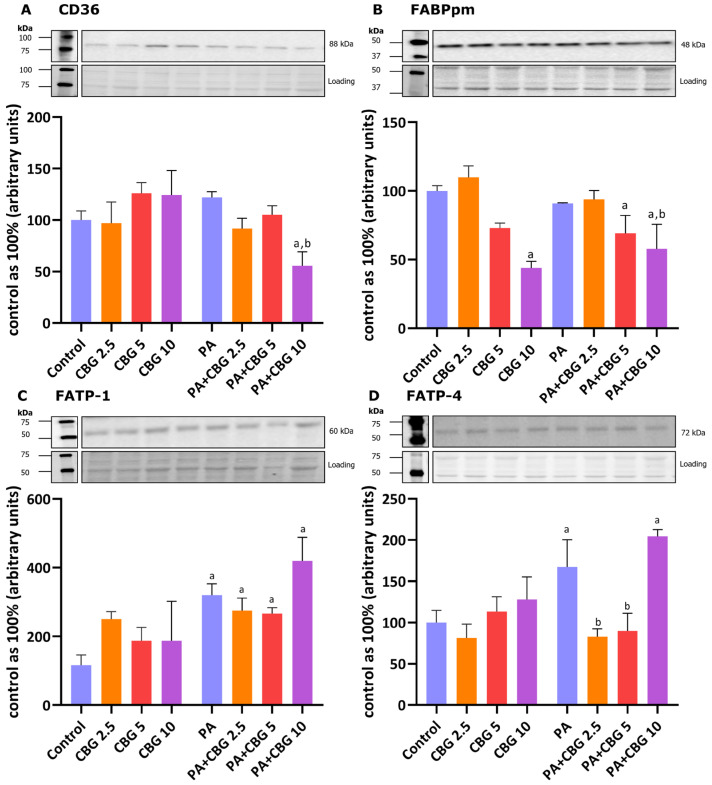
The levels of fatty acid transport proteins, including fatty acid translocase—CD36 (**A**), plasma membrane fatty acid-binding protein—FABPpm (**B**), fatty acid transport protein 1 and 4—FATP-1 and FATP-4 (**C**,**D**, respectively) in H9c2 cardiomyocytes after 18 h incubation with palmitate (PA; 300 μM) and/or cannabigerol (CBG; 2.5 μM, 5 μM, 10 μM). The levels of the abovementioned proteins are presented as percentage differences compared to the control group, which was set as 100%. The data are expressed as mean values ± SD and are based on three independent determinations in each group; ^a^ *p* < 0.05 indicates a significant difference: the control group in comparison to the examined group; ^b^ *p* < 0.05 indicates a significant difference: the PA group in comparison to the examined group.

To gain a further understanding of the CBG effect on lipid metabolism in H9c2 cardiomyocytes, we also analyzed the proteins involved in FA transport by evaluating the levels of FA CD36, FABPpm, FATP-1, and FATP-4 in control and lipid overload conditions. The incubation of H9c2 cardiomyocytes with PA for 18 h pronouncedly elevated FATP-1 and FATP-4 (+238.55%, +67.29%, *p* < 0.05 vs. the control group, respectively) levels as presented in Figure 2C,D. The level of FATP-1 was additionally increased in groups incubated with PA and all examined CBG concentrations (PA + CBG 2.5 µM: +193.57%, PA + CBG 5 µM: +166.98%, PA + CBG 10 µM: +351.99%, *p* < 0.05 vs. the control group; Figure 2C), whereas the similar effect was observed in FATP-4 level only with the highest CBG concentration (CBG 10 µM: +104.41%, *p* < 0.05 vs. the control group; Figure 2D). Interestingly, the FATP-4 level, which was elevated by 18 h PA treatment, was further diminished by the introduction of 2.5 µM and 5 µM CBG (−50.49% and −46.23%, *p* < 0.05 vs. the PA group, respectively; Figure 2D). Both CD36 and FABPpm levels were considerably decreased by the highest CBG concentration (10 µM) in the lipid overload conditions compared to the control H9c2 cells (−50.20% and −44.44%, *p* < 0.05, respectively; Figure 2A,B), as well as compared to the PA group alone (−51.11% and −54.41%, *p* < 0.05, respectively; Figure 2A,B). Additionally, the level of FABPpm was decreased in the PA + CBG 5 µM group (−30.80%, *p* < 0.05 vs. the control group; Figure 2B). The highest CBG concentration of 10 µM decreased the FABPpm level in H9c2 cardiomyocytes (−56.07%, *p* < 0.05; Figure 2B) compared to the control cells.

### 3.3. The Influence of 18 h PA and/or CBG Treatment on the Level of Proteins Involved in Fatty Acid Metabolism in H9c2 Cardiomyocytes

**Figure 3:**
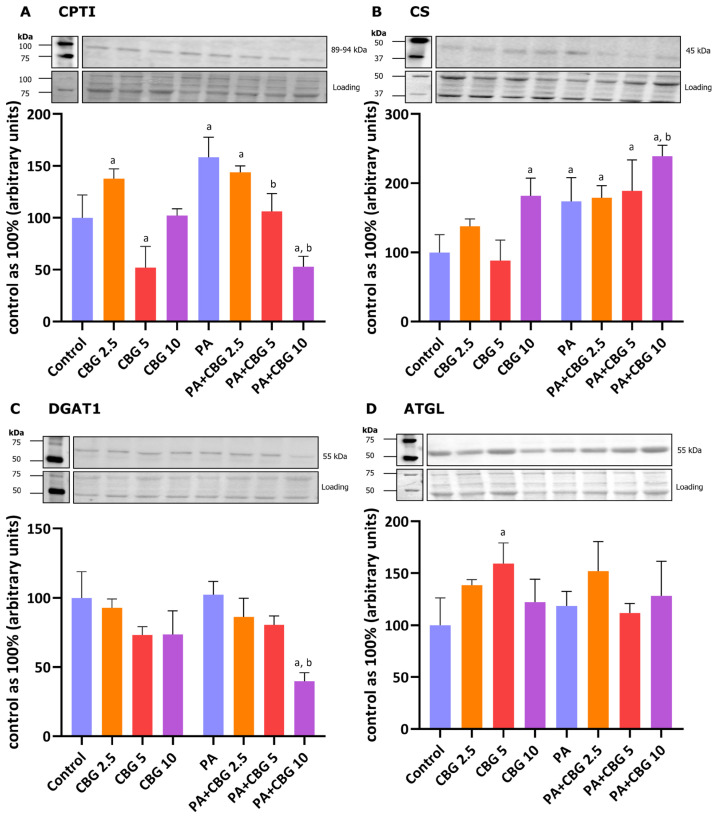
The levels of proteins involved in fatty acid metabolism, including carnitine palmitoyltransferase I—CPTI (**A**), citrate synthase—CS (**B**), diacylglycerol O-acyltransferase 1—DGAT1 (**C**), and adipose triglyceride lipase—ATGL (**D**) in H9c2 cardiomyocytes after 18 h incubation with palmitate (PA; 300 μM) and/or cannabigerol (CBG; 2.5 μM, 5 μM, 10 μM). The levels of the abovementioned proteins are presented as percentage differences compared to the control group, which was set as 100%. The data are expressed as mean values ± SD and are based on three independent determinations in each group; ^a^ *p* < 0.05 indicates a significant difference: the control group in comparison to the examined group; ^b^ *p* < 0.05 indicates a significant difference: the PA group in comparison to the examined group.

We also analyzed the levels of CPTI, CS, DGAT1, and ATGL, which are involved in maintaining energetic balance in cardiomyocytes, specifically the regulation of FA oxidation and their cellular turnover. As shown in Figure 3A, the level of CPTI was increased after treatment with 2.5 µM CBG (+37.69% *p* < 0.05 vs. the control group). In parallel, a higher CBG concentration (5 µM) significantly influenced CPTI and ATGL levels in the H9c2 cardiomyocytes (−47.87% and +17.16%, *p* < 0.05 vs. the control group, respectively; Figure 3A,D). In contrast, the highest examined concentration (10 µM) of this pCB had changed the content of CS (+82.05%, *p* < 0.05 vs. the control group; Figure 3B). The incubation of the cardiomyocytes with PA elevated the levels of both CPTI and CS (+58.23% and +73.75%, *p* < 0.05, respectively; Figure 3A,B), while simultaneous incubation with CBG and PA considerably influenced the content of CPTI (PA + CBG 2.5 µM: +43.71%, PA + CBG 10 µM: −47.15%, *p* < 0.05; Figure 3A), CS (PA + CBG 2.5 µM: +79.13%, PA + CBG 5 µM: +89.04%, PA + CBG 10 µM: +138.97%, *p* < 0.05; Figure 3B), and DGAT1 (PA + CBG 10 µM: −60.15%, *p* < 0.05; Figure 3C) compared to the control conditions. Furthermore, the levels of the same proteins, i.e., CPTI (PA + CBG 5 µM: −32.81%, PA + CBG 10 µM: −66.60%, *p* < 0.05; Figure 3A), CS (PA + CBG 10 µM: +37.54%, *p* < 0.05; Figure 3B), and DGAT1 (PA + CBG 10 µM: −61.08%, *p* < 0.05; Figure 3C) were altered by different concentrations of CBG in comparison to the PA group.

### 3.4. The Influence of 18 h PA and/or CBG Treatment on the Levels of Proteins Involved in the Insulin Signaling Pathway in H9c2 Cardiomyocytes

**Figure 4:**
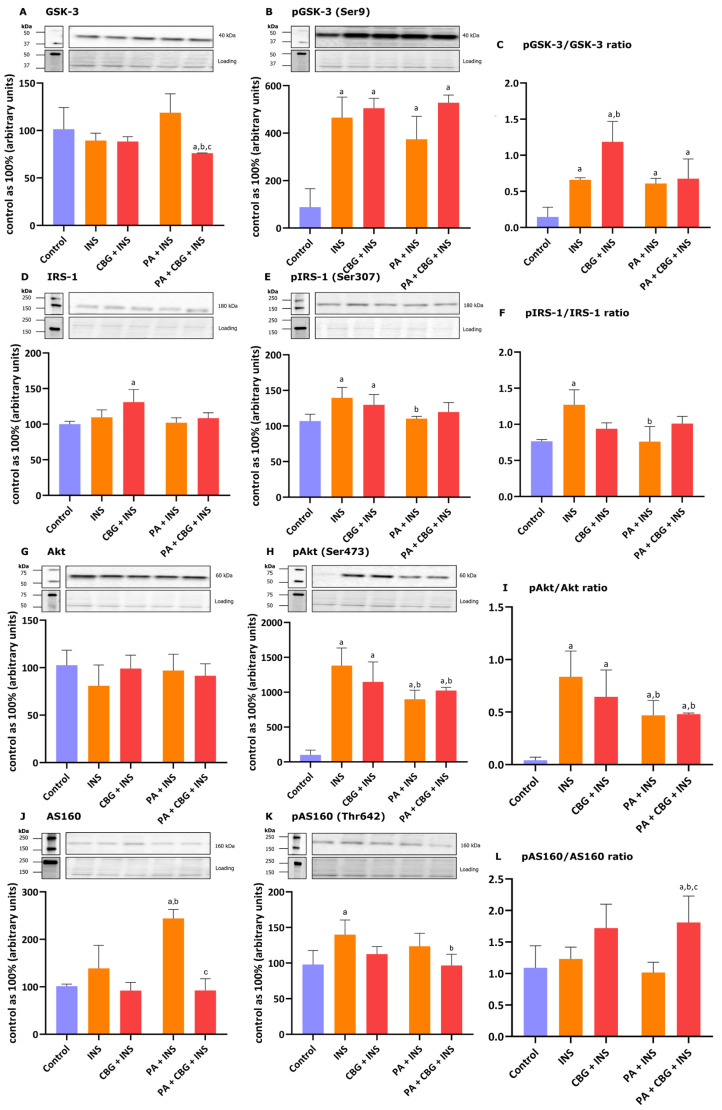
The levels of proteins involved in insulin singling pathway, including glycogen synthase kinase 3—GSK-3 (**A**), phosphorylated glycogen synthase kinase 3—pGSK3 (Ser9) (**B**), insulin receptor substrate 1—IRS-1 (**D**), phosphorylated insulin receptor substrate 1—pIRS1(Ser307) (**E**), protein kinase B—Akt (**G**), phosphorylated protein kinase B—pAkt (Ser473) (**H**), Akt substrate of 160 kDa—AS160 (**J**), and phosphorylated Akt substrate of 160 kDa—pAS160 (Thr642) (**K**) as well as pGSK-3/GSK-3 (**C**), pIRS-1/IRS-1 (**F**), pAkt/Akt (**I**), and pAS160/AS160 (**L**) ratios in H9c2 cardiomyocytes after 18 h incubation with palmitate (PA; 300 μM) and/or cannabigerol (CBG; 10 μM) and 10 min incubation with insulin (INS; 100 nM). The levels of the abovementioned proteins are presented as percentage differences compared to the control group which was set as 100%. The data are expressed as mean values ± SD and are based on four independent determinations in each group; ^a^ *p* < 0.05 indicates a significant difference: the control group in comparison to the examined group; ^b^ *p* < 0.05 indicates a significant difference: the INS group in comparison to the examined group; and ^c^ *p* < 0.05 indicates a significant difference: the PA group in comparison to the PA + CBG + INS group.

To further evaluate the role of CBG as a potential metabolic modulator of lipid metabolism and related signaling pathways in H9c2 cardiomyocytes, we studied the levels of selected proteins involved in insulin signaling after the incubation of the cells with insulin in different conditions. Figure 4A shows that the 10 µM CBG concentration in lipid overload conditions markedly diminished the level of GSK-3 (−27.84%, *p* < 0.05 vs. the control group; −14.97%, *p* < 0.05 vs. the INS group; −36.01%, *p* < 0.05 vs. the PA + INS group) in the H9c2 cardiomyocytes incubated with the insulin for 10 min. The level of pGSK-3 was elevated in each group (INS: +356.76%; CBG + INS: +375.83%; PA + INS: +273.76%; PA + CBG + INS: +421.84%, *p* < 0.05; Figure 4B) compared to the control H9c2 cardiomyocytes. Additionally, the pGSK/GSK ratio was found to be increased in the INS group (+270.70% *p* < 0.05 vs. the control group), CBG + INS group (+548.36%, *p* < 0.05 vs. the control group; +74.90%, *p* < 0.05 vs. the INS group), PA + INS group (+249.73%, *p* < 0.05 vs. the control group), as well as PA + CBG + INS group (+315.95%, *p* < 0.05 vs. the control group; Figure 4C). In regard to IRS-1 and pIRS-1, the levels of both compounds were considerably changed after incubation with CBG and insulin (+31.08% and +29.72%, *p* < 0.05, respectively; Figure 4D,E) compared to the control cells. The level of pIRS-1 was additionally changed in the INS (+39.47%, *p* < 0.05 vs. the control group) as well as PA + INS (−22.09%, *p* < 0.05 vs. the INS group; Figure 4E) groups. In parallel, the pIRS/IRS ratio was significantly changed in the INS group (+57.79%, *p* < 0.05 vs. the control group) and PA + INS group −13.19%, *p* < 0.05 vs. the INS group; Figure 4F). Moreover, the phosphorylation of Akt and the pAkt/Akt ratio were meaningfully raised in all experimental groups compared to the control group, namely: INS (+1282.33%, +1974.61%, *p* < 0.05, respectively), CBG + INS (+1107.96%, +1601.13%, *p* < 0.05, respectively), PA + INS (+799.22%, +1072.18%, *p* < 0.05, respectively), and PA + CBG + INS (+910.00%, +1108.70%, *p* < 0.05, respectively; Figure 4H,I). In addition, we found that the pAkt level and the pAkt/Akt ratio were decreased in the PA + INS (−34.95%, −43.50%, *p* < 0.05, respectively) as well as the PA + CBG + INS (−26.94%,−41.74%, *p* < 0.05, respectively; Figure 4H,I) groups compared to the INS group. The level of AS160 phosphorylation increased after insulin treatment (+39.97%, *p* < 0.05, vs. the control group; Figure 4K). Furthermore, the level of AS160 was meaningfully raised after the incubation of H9c2 cardiomyocytes with insulin in lipid overload conditions (+136.41%, *p* < 0.05 vs. the control group; +70.36%, *p* < 0.05 vs. the INS group; Figure 4J). The levels of AS160 and pAS160 were lower after simultaneous incubation of cells with PA, CBG, and insulin (AS160: −61.15%, vs. the PA + INS group; pAS160: −30.98%, vs. the INS group; Figure 4J, K). The pAS160/AS160 ratio was markedly increased in PA + CBG + INS (+70.96%, *p* < 0.05, vs. the control group; +64.82%, *p* < 0.05, vs. the INS group; +95.59%, vs. the PA + INS group; Figure 4L) group.

### 3.5. The Influence of 18 h PA and/or CBG Treatment on the 2-Deoxyglucose Uptake by H9c2 Cardiomyocytes

**Figure 5:**
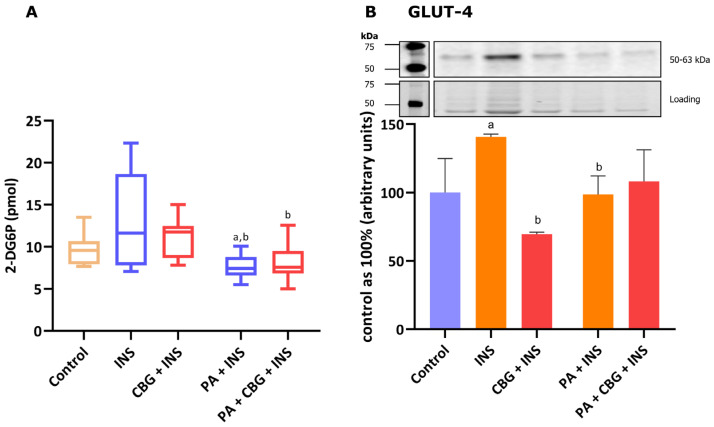
The level of 2-deoxyglucose uptake (**A**) and the levels of glucose transporter type 4—GLUT-4 (**B**) in H9c2 cardiomyocytes after 18 h incubation with palmitate (PA; 300 μM) and/or cannabigerol (CBG; 10 μM) and 10 min incubation with insulin (INS; 100 nM). The data are expressed as mean values ± SD and are based on sixteen (**A**) or four (**B**) independent determinations in each group; ^a^ *p* < 0.05 indicates a significant difference: the control group in comparison to the examined group; ^b^ *p* < 0.05 indicates a significant difference: the INS group in comparison to the examined group.

In this paragraph, we additionally explored if the impaired glucose uptake induced by incubation with PA in H9c2 cardiomyocytes can be affected by CBG. As presented in Figure 5A, we observed a meaningful alteration in the level of glucose uptake after PA and insulin treatment, both compared to the control group (−20.96%, *p* < 0.05) and insulin group (−41.83%, *p* < 0.05) of H9c2 cardiomyocytes. The incubation of the cells with CBG, insulin, and PA diminished the level of glucose uptake by H9c2 cardiomyocytes (−38.08%, *p* < 0.05; Figure 5A) compared to the cells incubated with insulin. However, the CBG treatment of H9c2 cardiomyocytes in the presence of PA and insulin has not influenced the glucose uptake that was already diminished after incubation with PA.

### 3.6. The Influence of 18 h PA and/or CBG Treatment on the Level of Glucose Transporter in H9c2 Cardiomyocytes

Figure 5B demonstrates that 10 min of incubation of H9c2 cells with insulin markedly increased the content of GLUT-4 (+40.82%, *p* < 0.05, vs. the control group). The level of the transporter was lower after the 18 h incubation of cells with insulin and CBG (−50.59%, *p* < 0.05), as well as insulin and PA (−33.45%, *p* < 0.05; Figure 5B) compared to the insulin group.

### 3.7. The Influence of 18 h PA and/or CBG Treatment on the Levels of Proteins Involved in the Metabolism of C20:4 in H9c2 Cardiomyocytes

**Figure 6:**
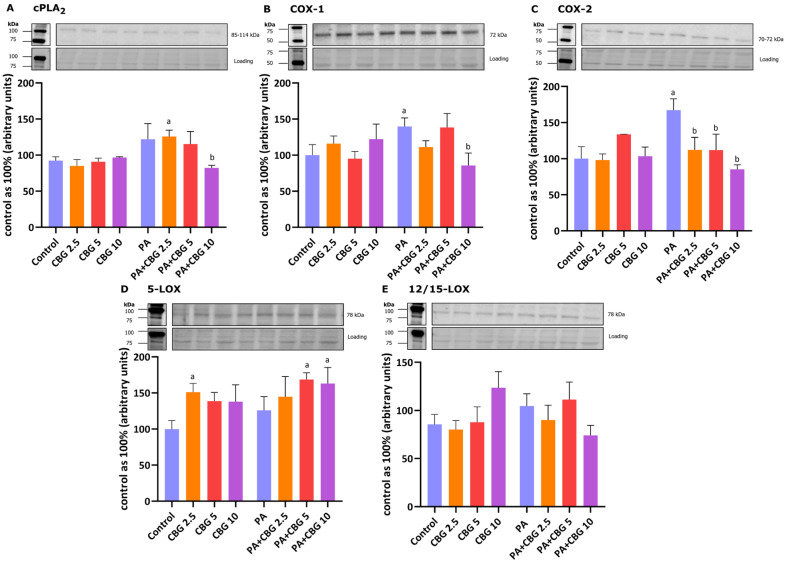
The levels of cytosolic phospholipase A_2_—cPLA_2_ (**A**), cyclooxygenase 1 and 2—COX-1 (**B**) and COX-2 (**C**), and 5-lipoxygenase—15-LOX (**D**) as well as 12/15-lipoxygenase—12/15-LOX (**E**) in H9c2 cardiomyocytes after 18 h incubation with palmitate (PA; 300 μM) and/or cannabigerol (CBG; 10 μM). The data are expressed as mean values ± SD and are based on three independent determinations in each group; ^a^ *p* < 0.05 indicates a significant difference: the control group in comparison to the examined group; ^b^ *p* < 0.05 indicates a significant difference: the PA group in comparison to the examined group.

To evaluate the anti-inflammatory effects of CBG, we examined the levels of proteins engaged in the metabolism of arachidonic acid in H9c2 cells. We observed an elevated level of 5-LOX after the incubation of cells with 2.5 µM CBG (+51.14%, *p* < 0.05, vs. the control group; Figure 6D). Figure 6B,C show that the levels of COX-1 and COX-2 were significantly raised by the incubation of cardiomyocytes with PA (+39.80% and +67.25%, *p* < 0.05, respectively) in comparison to the cardiomyocytes incubated in the control medium. The levels cPLA_2_, COX-1, and COX-2 were meaningfully decreased by the concomitant incubation with PA and CBG in the concentration of 10 µM (−32.33%, −38.66%, −49.08%, *p* < 0.05, respectively; Figure 6A–C) in comparison to the PA group. Moreover, the content of cPLA_2_ was increased in the PA + CBG 2.5 µM group (+36.01%, *p* < 0.05; Figure 6A), whereas the level of 5-LOX was elevated in the PA + CBG 5 µM and PA + CBG 10 µM groups (+68.63%, +63.12%, *p* < 0.05, respectively; Figure 6D) in comparison to the control group. Lastly, the level of COX-2 in H9c2 cardiomyocytes was lowered in the PA + CBG 2.5 µM and PA + CBG 5 µM groups (−32.87% and −33.11%, *p* < 0.05; respectively; Figure 6C) in comparison to the PA group.

### 3.8. The Influence of 18 h PA and/or CBG Treatment on the Components of the Endocannabinoid System in H9c2 Cardiomyocytes

**Figure 7:**
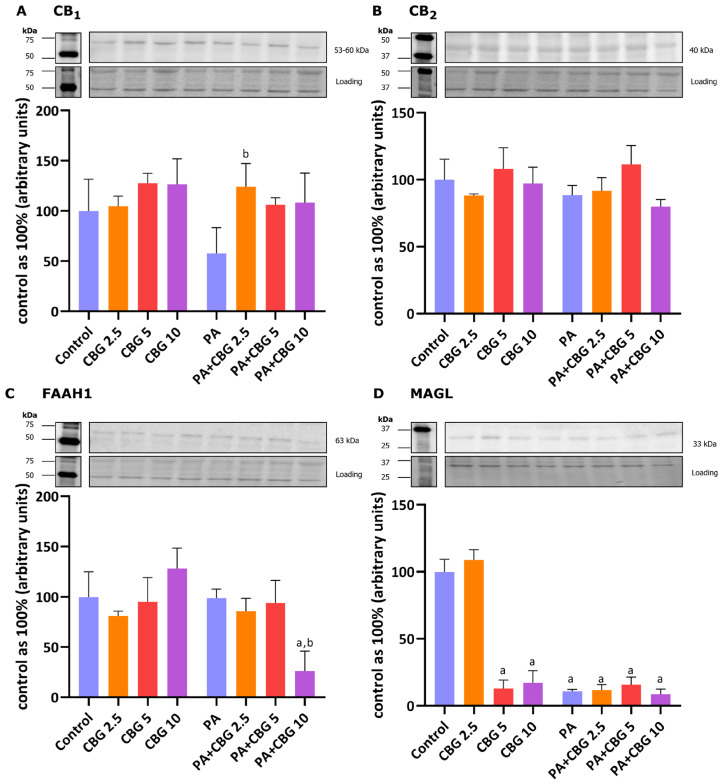
The levels of cannabinoid receptor 1—CB_1_ (**A**), cannabinoid receptor 2—CB_2_ (**B**), fatty-acid amide hydrolase 1—FAAH1 (**C**), and monoacylglycerol lipase—MAGL (**D**) in H9c2 cardiomyocytes after 18 h incubation with palmitate (PA; 300 μM) and/or cannabigerol (CBG; 10 μM). The data are expressed as mean values ± SD and are based on three independent determinations in each group; ^a^ *p* < 0.05 indicates a significant difference: the control group in comparison to the examined group; ^b^ *p* < 0.05 indicates a significant difference: the PA group in comparison to the examined group.

In our experiment, the incubation of H9c2 cardiomyocytes with PA and 2.5 µM CBG increased the level of CB_1_ (+115.47%, *p* < 0.05; Figure 7A) in comparison to the H9c2 cells incubated with PA. As demonstrated in Figure 7C,D, the levels of FAAH1 and MAGL were markedly lowered after the concomitant treatment of cardiomyocytes with PA and 10 µM CBG (FAAH1: −73.93%, *p* < 0.05, vs. the control group; −73.59%, *p* < 0.05, vs. the PA group; MAGL: −91.46% *p* < 0.05, vs. the control group). Additionally, the level of MAGL was reduced in the CBG 5 µM (−87.01%, *p* < 0.05), CBG 10 µM (−82.84%, *p* < 0.05), PA (−89.19%, *p* < 0.05), PA + CBG 2.5 µM (−88.31%, *p* < 0.05), and PA + CBG 5 µM (−84.26%, *p* < 0.05; Figure 7D) groups in comparison to the control group.

### 3.9. The Influence of 18 h PA and/or CBG Treatment on the Fatty Acid Composition of the Free Fatty Acid Fraction in H9c2 Cardiomyocytes

Moreover, we determined whether CBG application affects FA composition in all examined lipid fractions in H9c2 cardiomyocytes in control and lipid overload conditions. Table 1 demonstrates that the levels of C20:0 and C24:0 were lower in the FFA pool after incubation with CBG in the concentrations of 2.5 µM (−57.38%; −35.60%, *p* < 0.05, respectively) and 5 µM (−65.23%; −37.34%, *p* < 0.05, respectively; Table 1) compared to the control cells. CBG in the concentration of 10 µM raised the level of C22:0 (+85.01% and +104.42%, *p* < 0.05, Table 1) in comparison to CBG 2.5 and CBG 5 groups, respectively. The content of C20:0 was also markedly elevated after the treatment with 10 µM CBG (+91.27% and + 134.46%, *p* < 0.05; Table 1) compared to the CBG 2.5 and CBG 5 groups, respectively. H9c2 cardiomyocytes treated with PA had increased levels of C16:0, C16:1, C18:0, and C18:3 (+164.61%, +45.31%, +38.83%, +101.73%, *p* < 0.05, respectively; Table 1), compared to the control cardiomyocytes. Significant changes were also observed in C14:0 and C16:0 contents after incubation with PA and CBG in the concentration of 2.5 µM (C14:0: +116.09%; C16:0: +219.43%, *p* < 0.05 vs. the control group; C14:0: +120.87%; C16:0: +20.72%, *p* < 0.05 vs. the PA group), 5 µM (C14:0: +122.40%; C16:0: +318.50%, *p* < 0.05 vs. the control group; C14:0: +127.32%; C16:0: +58.16%, *p* < 0.05 vs. the PA group), and 10 µM (C14:0: +79.41%; C16:0: +262.34%, *p* < 0.05 vs. the control group; C14:0: +83.38%; C16:0: +36.93%, *p* < 0.05 vs. the PA group; Table 1). In regard to C22:0, C20:4, and C24:0, their contents were influenced by the simultaneous treatment with PA and CBG in the concentrations of 2.5 µM (+46.91%; +102.84%; −100%, *p* < 0.05 vs. the control group, respectively; +39.68%; +53.99%; −100%, *p* < 0.05 vs. the PA group, respectively; Table 1) and 5 µM (+99.37%; +208.91%; −100%, *p* < 0.05 vs. the control group, respectively; +89.57%; +134.52%; −100%, *p* < 0.05 vs. the PA group, respectively; Table 1), whereas the contents of C18:0 and C18:2 in H9c2 cardiomyocytes were influenced by the incubation with PA and CBG in the concentrations of 5 µM (+77.30%; +68.25%, *p* < 0.05 vs. the control group, respectively; +27.71%; +60.68%, *p* < 0.05 vs. the PA group, respectively) and 10 µM (+103.95%; +48.84%, *p* < 0.05 vs. the control group, respectively; +46.91%; +42.14%, *p* < 0.05 vs. the PA group, respectively); and the content of C18:3 was increased by incubation with PA and CBG in the concentration of 5 µM (+157.77%, *p* < 0.05 vs. the control group; +27.78%, *p* < 0.05 vs. the PA group; Table 1). As presented in Table 1, the content of C18:1 in the cardiomyocytes raised after incubation with PA and CBG in the concentration of 2.5 µM (+37.52%, *p* < 0.05), 5 µM (+54.71%, *p* < 0.05), and 10 µM (+50.58%, *p* < 0.05) compared to the cardiomyocytes treated with PA. Additionally, we observed an increase in the content of C16:1 (PA + 5 µM CBG: +67.33%, *p* < 0.05), C18:0 (PA + 2.5 µM CBG: +46.19%, *p* < 0.05), C20:4 (PA + 10 µM CBG: +99.12%, *p* < 0.05), and C18:3 (PA + 2.5 µM CBG: +83.05% and PA + 10 µM CBG: +107.34%, *p* < 0.05; Table 1) compared to the control cells. SFA content in the FFA fraction was significantly increased after incubation with PA (+49.07%, *p* < 0.05) as well as with PA and 2.5 µM CBG (+84.73%, *p* < 0.05), 5 µM (+69.84%, *p* < 0.05), and 10 µM CBG (+125.37%, *p* < 0.05; Table 1) compared to the control group. Additionally, after the concomitant treatment of cells with PA and 5 µM CBG we observed changed contents of C16:0, C16:1, C18:3, C22:0, and C20:4 (+31.02, +42.51%, +40.82%, +35.71%, +52.29%, *p* < 0.05, respectively; Table 1) compared to the PA + CBG 2.5 group. In the PA CBG 10 µM group, alterations were observed in the quantities of C18:0 and SFAs (+39.51%, +22.00%, *p* < 0.05, respectively) in comparison to the PA + CBG 2.5 group, whereas the quantities of C14:0, C16:0, C22:0, C20:4, SFAs, and MUFAs (−19.33%, −13.42%, −31.73%, −35.54%, +32.70%, +43.72%, *p* < 0.05, respectively; Table 1) were influenced compared to the PA + CBG 5 group. The content of SFAs was also increased after treatment with PA and 2.5 µM (+23.92%, *p* < 0.05) as well as 10 µM CBG (+51.19%, *p* < 0.05; Table 1) compared to the PA group. In contrast, the content of MUFA fraction decreased in the PA (−23.27%, *p* < 0.05) and PA + 5 µM CBG (−24.90%, *p* < 0.05) groups compared to the control cells, and simultaneously increased in the PA + 10 µM CBG (+40.68%, *p* < 0.05; Table 1) group compared to the PA group. Finally, the amount of PUFAs was significantly raised in the PA + 5 µM CBG group (+39.89%, *p* < 0.05 vs. the control group) and in the PA + 10 µM CBG group compared to both the control and PA group (+53.35% and +30.79%, *p* < 0.05, respectively; Table 1). The content of PUFAs was also markedly elevated after the treatment with 10 µM CBG (+62.84% and + 48.58%, *p* < 0.05; Table 1) compared to the CBG 2.5 and CBG 5 groups, respectively.

### 3.10. The Influence of 18 h PA and/or CBG Treatment on the Fatty Acid Composition of the Diacylglycerol Fraction in H9c2 Cardiomyocytes

The incubation of H9c2 cardiomyocytes with 5 µM CBG changed the content of C14:0 (−42.83%, *p* < 0.05) and C18:2 (+140.67%, *p* < 0.05; Table 2) in the DAG fraction compared to the cells incubated in the control medium. Cells incubated with 10 µM CBG displayed a markedly lowered content of C14:0 (−43.44% *p* < 0.05) compared to the CBG 2.5 group and a raised amount of C18:2 (+333.33%, *p* < 0.05; Table 2) compared to the CBG 5 group. Table 2 also shows that in the PA group, the amounts of C16:0 and C18:0 (+747.90%, +48.74%, *p* < 0.05, respectively) were increased compared to the control cells. The concomitant incubation of H9c2 cardiomyocytes with PA and CBG in all studied concentrations significantly changed the contents of C16:0, C20:0, C22:0, and C24:0 (PA + 2.5 µM CBG: +490.93%, +1310.26%, +1779.57%, +900.51%, PA + 5 µM CBG: +441.21%, +1648.25%, +2406.96%, +1025.58%, PA + 10 µM CBG: +407.22%, +963.52%, +1350.03%, +702.30%, *p* < 0.05, respectively) compared to the control H9c2 cardiomyocytes as well as the cells incubated with PA (PA + 2.5 µM CBG: −30.31%, +1114.85%, +847.51%, +585.41%; PA + 5 µM CBG: −36.17%, +1406.02%, +1163.79%, +671.09%; PA + 10 µM CBG: −40.18%, +816.16%, +630.98%, +449.62%, *p* < 0.05, respectively; Table 2). In the PA + 2.5 µM CBG group the changes were found in the amounts of C18:0, C18:1, and C18:2 (+56.35%, +161.50%, +145.93%, *p* < 0.05 vs. the control group, respectively), C14:0 and C20:4 (+54.46%, +168.78% vs. the control group, and +43.76%, +179.40% vs. the PA group, *p* < 0.05, respectively; Table 2) in H9c2 cardiomyocytes. Moreover, in the PA + 5 µM CBG group, there were changes observed in the amounts of C14:0, C16:1, C18:0, and C18:1 (+37.77%, +156.46%, +73.94%, +143.34%, *p* < 0.05, respectively; Table 2) compared to the control H9c2 cells. The concomitant treatment of cardiomyocytes with PA and 5 µM CBG influenced the quantities of C16:1, C20:0, and C22:0 (+88.67%, +23.97%, +33.38%, *p* < 0.05, respectively), whereas incubation with PA and 10 µM CBG changes the contents of C14:0, C16:0, C16:1, C20:0, C18:3, C22:0, and SFAs (−27.00, −14.17%, +139.72%, −24.59%, +112.84%, −22.85%, −22.29%, *p* < 0.05, respectively; Table 2) compared to the PA + CBG 2.5 group. In the PA + CBG 10 µM group the changes were also observed in the amount of C18:0, C20:0, C22:0, C24:0, and SFAs (−30.11%, −39.17%, −42.16%, −28.72%, −18.90%, *p* < 0.05, respectively; Table 2) compared to the PA + CBG 5 group. In the group with the highest concentration of CBG (PA + 10 µM CBG), we noticed that C18:3, C16:1, and C20:4 (+126.24%, +225.86%, +132.20%, *p* < 0.05, respectively) were raised compared to the control cells and C16:1 along with C20:4 (+75.33%, +141.38%, *p* < 0.05, respectively; Table 2) were increased compared to the PA group. The content of SFAs was raised significantly after PA (+360.73%, *p* < 0.05) treatment as well as PA + CBG incubation in the concentration of 2.5 µM (+330.13%, *p* < 0.05), 5 µM (+312.12%, *p* < 0.05), and 10 µM (+234.24%, *p* < 0.05; Table 2), compared to the control group. Additionally, as presented in Table 2, the highest CBG concentration (10 µM) considerably diminished the SFA level compared to the PA group (−27.45%, *p* < 0.05). The quantity of MUFAs in the DAG fraction was elevated after PA and CBG treatment in the concentrations of 2.5 µM (+139.67%, *p* < 0.05), 5 µM (+117.51%, *p* < 0.05), and 10 µM (+144.84%, *p* < 0.05), whereas only 2.5 µM (+193.33%, *p* < 0.05) and 5 µM (+134.98%, *p* < 0.05; Table 2) CBG influenced the content of PUFAs, all compared to the control group. The content of MUFAs was additionally increased after simultaneous incubation with PA and 10 µM CBG compared to the PA group (+56.66%, *p* < 0.05; Table 2).

### 3.11. The Influence of 18 h PA and/or CBG Treatment on the Fatty Acid Composition of the Triacylglycerol Fraction in H9c2 Cardiomyocytes

As demonstrated in Table 3, the contents of C16:1, C18:0, C18:1, C18:2, and C18:3 in the TAG fraction were influenced by CBG in the concentrations of 2.5 µM (−51.75%; +60.43%; +106.91%; +258.00%; −53.32%, *p* < 0.05, respectively) and 5 µM (−44.52%; +110.12%; +84.97%; +120.53%; −49.22%, *p* < 0.05, respectively) and the amount of C20:4 only by CBG in the concentration of 2.5 µM (−42.87%, *p* < 0.05; Table 3), all compared to the control group. Additionally, the quantities of C18:2 and C20:4 were altered after incubation with 5 µM CBG (−38.40% and −51.84%, *p* < 0.05; respectively; Table 3) in comparison to the CBG 2.5 group. H9c2 cardiomyocytes incubated with 10 µM CBG caused alternations in the levels of C16:1, C20:0, C18:3, C22:0, and C20:4 (−32.65%, +65.32%, −74.34%, −41.67%, −48.07%, *p* < 0.05, respectively; Table 3) compared to the control H9c2 cardiomyocytes and in the levels C18:1, C18:2, and C18:3 (−47.48%, −77.42%, −45.03%, *p* < 0.05; respectively) compared to the CBG 2.5 group, as well as C14:0, C18:0, C18:1, C18:2, C18:3, and C20:4 (−28.85%, −37.66%, −41.26%, −63.34%, −49.46%, −40.13%, *p* < 0.05; respectively; Table 3) in comparison to the CBG 5 group. The incubation of the examined cardiomyocytes for 18 h with PA changed the contents of C16:0, C16:1, C18:0, C20:0, C18:3, and C20:4 (+1875.27%, −35.75%, +59.22%, +116.65%, −72.15%, −40.28%, *p* < 0.05, respectively; Table 3) compared to the control cells. Moreover, the introduction of 2.5 µM CBG to the PA group caused changes in the contents of C14:0, C16:0, C16:1, C18:0, C18:2, C20:0, C18:3, C20:4 (+68.94%, +3314.54%, −40.14%, +114.06%, +100.55%, +77.99%, −68.06%, −44.14%, *p* < 0.05 vs. the control group; respectively), as well as C14:0, C16:0, C18:1, and C18:2 (+59.13%, +72.86%, +140.12%, +129.95%, *p* < 0.05 vs. the PA group, respectively; Table 3). Simultaneously, treatment with 5 µM and 10 µM CBG markedly diminished the content of C20:0 heightened by the incubation with PA (5 µM: −30.15%; 10 µM: −47.69%, *p* < 0.05 vs. the PA group; Table 3) in the H9c2 cardiomyocytes. The simultaneous incubation of the cells with PA and 5 µM CBG also influenced the amounts of C14:0, C16:0, C16:1, C18:0, and C18:3 (+92.77%, +3835.35%, +48.37%, +145.12%, −53.86%, *p* < 0.05, respectively) compared to the control H9c2 cardiomyocytes and (+81.59%, +99.23%, +130.92%, +53.95%, +65.69%, *p* < 0.05, respectively; Table 3) compared to the H9c2 cardiomyocytes treated with PA. Additionally, in the PA + CBG 5 µM group the amounts of C16:0 and C16:1 were changed (+147.84%, +14.40% *p* < 0.05; respectively), whereas in the PA + CBG 10 µM group the contents of C18:1, C18:2, C20:0, and PUFAs (−49.67%, −70.93%, −36.33%, −57.71%, *p* < 0.05; respectively; Table 3) were altered in comparison to the PA + CBG 2.5 group. The contents of C14:0, C16:0, C16:1, and SFAs were influenced by the treatment of cells with PA and 10 µM CBG (−21.91%, −14.01%, −43.88%, −14.69%, *p* < 0.05; respectively; Table 3) in comparison to the PA + CBG 5 group. Table 3 also presents that in the PA + 10 µM CBG group, the contents of C18:0, C18:3, and C20:4 (+104.52%, −66.97%, −39.41%, *p* < 0.05) were markedly altered compared to the control group, whereas the levels of C14:0 and C16:0 (+50.54% and +3284.17%, as well as +41.81% and +71.33%, *p* < 0.05; Table 3) were substantially changed compared to the control and PA groups, respectively. Furthermore, the level of SFAs within TAG fraction was considerably elevated in the PA (+818.35%, *p* < 0.05 vs. the control group), PA + 2.5 µM CBG (+1464.37%, *p* < 0.05 vs. the control group; +70.35%, *p* < 0.05 vs. the PA group), PA + 5 µM CBG (+1689.68%, *p* < 0.05 vs. the control group; +94.88%, *p* < 0.05 vs. the PA group), and PA + 10 µM CBG (+1426.83%, *p* < 0.05 vs. the control group; +66.26%, *p* < 0.05 vs. the PA group; Table 3) groups in H9c2 cardiomyocytes. The content of MUFAs in the TAG fraction was increased only after incubation with 2.5 µM (+65.43%, *p* < 0.05) and 5 µM (+52.09%, *p* < 0.05) CBG compared to the control group, and after incubation with 2.5µM CBG and PA (+75.45%, −29.86%, *p* < 0.05; Table 3) compared to the PA group. Finally, the amount of PUFAs substantially increased only after 2.5 µM CBG treatment (+104.76%, *p* < 0.05) compared to the control group and 2.5 µM CBG and PA treatment (+103.23%, *p* < 0.05; Table 3) compared to the PA group.

### 3.12. The Influence of 18 h PA and/or CBG Treatment on the Fatty Acid Composition of the Phospholipid Fraction in H9c2 Cardiomyocytes

In the PL pool, the contents of C16:1, C20:4, and C20:5 were significantly increased in the H9c2 cardiomyocytes in the groups incubated with 2.5 µM CBG (+293.42%; +22.68%; +85.91%, *p* < 0.05, respectively), 5 µM CBG (+286.55%; +34.87%; +94.02%, *p* < 0.05, respectively), and 10 µM CBG (+212.96%; +22.87%; +72.22%; *p* < 0.05, respectively; Table 4) compared to the cardiomyocytes incubated in the control medium. CBG in the concentration of 2.5 µM also increased the amounts of C18:1, C18:2, and C22:6 (+40.90%, +73.61%, +113.60%, *p* < 0.05, vs. the control cells, respectively). Meanwhile, CBG’s concentration of 5 µM elevated the content of C18:0 and C22:6 (+21.64%, +160.97%, *p* < 0.05, respectively), and the concentration of 10 µM increased the content of C20:0 (+142.75%, *p* < 0.05) in H9c2 cells compared to the control cells. Additionally, the contents of C16:1 and C18:1 were altered in the 10 µM CBG group (C16:1: −20.45%, −31.22%; C18:1: −19.04%, −26.14%, *p* < 0.05, respectively; Table 4) compared to the CBG 2.5 and CBG 5 groups, respectively. However, we noticed that PA increased the content of C16:0, C20:0, C22:0, C20:4, C20:5, and C22:6 (+496.66%, +179.87%, +186.80%, +24.32%, +100.63%, +133.13%, *p* < 0.05, respectively; Table 4) compared to the control cells. As demonstrated in Table 4, the concomitant treatment of the cells with PA and 2.5 µM CBG increased the levels of C14:0, C16:0, C16:1, C20:0, C18:3, C22:0, 20:5, and C22:6 (+214.62%, +588.52%, +142.15%, +279.51%, +224.92%, +288.77%, +85.44%, +119.03%, *p* < 0.05, respectively) compared to the control cells. It also changed the contents of C14:0 and C16:0 (+84.18% and +15.39%, *p* < 0.05, respectively) compared to the PA group. In regard to the PA + 5 µM CBG group, the content of almost each examined FA was influenced in H9c2 cardiomyocytes, namely: C14:0, C16:0, C16:1, C18:0, C18:2, C20:0, C18:3, C22:0, C20:4, C20:5, C22:6 (+268.24%, +639.66%, +288.41%, +21.46%, +108.66%, +343.01%, +418.15%, +561.15%, +24.76%, +132.64%, +296.30%, *p* < 0.05 vs. the control group, respectively), and C14:0, C16:0, C16:1, C18:0, C18:2, C20:0, C18:3, C22:0, and C22:6 (+115.57%, +23.97, +36.78%, +21.93%, +71.07%, +58.29%, +117.13%, +130.53%, +69.99%, *p* < 0.05 vs. the PA group, respectively; Table 4). We also observed changed contents of C16:1, C18:2, C18:3, C22:0, C22:6, and PUFAs in PA + CBG 2.5 µM (+60.40%, +67.18%, +59.47%, +70.06%, +80.93%, +47.04%, *p* < 0.05, respectively; Table 4) compared to the PA + CBG 2.5 group. Moreover, the simultaneous incubation of the cells with PA and 10 µM CBG changed the amounts of C14:0, C16:0, C16:1, C20:0, C18:3, C22:0, C20:5, and C22:6 (+135.23%, +578.44%, +152.37%, +244.35%, +169.79%, +185.22%, +79.76%, +258.94%, *p* < 0.05, respectively) compared to the control cardiomyocytes, as well as the level of C22:6 (+53.97%, *p* < 0.05; Table 4) compared to the PA group. In the PA + CBG 10 µM group we observed changed levels of C22:6 (+63.88%, *p* < 0.05) compared to the PA + CBG 2.5 group and of C16:1, C18:2, C18:3, C22:0, MUFAs, and PUFAs (−35.02%, −46.87%, −47.93%, −56.86%, −24.49%, −31.31%, *p* < 0.05, respectively; Table 4) compared to the PA + CBG 5 group. Additionally, the content of SFAs was markedly increased after incubation with PA (+257.40%, *p* < 0.05) compared to the control group, as well as in the PA + 2.5 µM CBG (+311.08%, *p* < 0.05 vs. the control group; +15.02%, *p* < 0.05 vs. the PA group), PA + 5 µM CBG (+333.99%, *p* < 0.05 vs. the control group; +21.43%, *p* < 0.05 vs. the PA group), and PA + 10 µM CBG (+308.13%, *p* < 0.05 vs. the control group; +14.20%, *p* < 0.05 vs. the PA group) groups. Moreover, the content of MUFAs was increased after incubation with all examined CBG concentrations (2.5 µM: +74.14%; 5 µM: +60.51%; 10 µM: +28.80%, *p* < 0.05) as well as in the group treated with 5 µM CBG and PA (+36.69%, *p* < 0.05; Table 4), all compared to the control group. The quantity of MUFAs was also changed after incubation with 10 µM CBG (−26.04%, *p* < 0.05 vs. the CBG 2.5 group; −19.76%, *p* < 0.05 vs. the CBG 5 group). Lastly, PUFAs were influenced by 2.5 µM CBG (+50.89%, *p* < 0.05), 5 µM CBG (+49.42%, *p* < 0.05), PA (+52.19%, *p* < 0.05), and PA + 5 µM CBG (+97.41%, *p* < 0.05) compared to the control group, and PA + 5 µM CBG (+29.71%, *p* < 0.05; Table 4) compared to the PA group. 

## 4. Discussion

In our research, H9c2 cardiac cells were exposed to control and lipid overload conditions, reflecting the harmful influence of overnutrition on the cardiac muscle, with the simultaneous introduction of an agent with potential cardioprotective properties, i.e., CBG. To the best of our knowledge, no published data, so far, have revealed the effects of CBG in the abovementioned conditions. In the current study, we mainly focused on the concentration-dependent effect of CBG on lipid profile and insulin signaling pathway in H9c2 cardiomyocytes. Importantly, we demonstrated that the metabolic response of the cardiomyocytes, in both control and lipid overload conditions, is different in the presence of lower (2.5 µM and 5 µM) and higher (10 µM) concentrations of CBG. The major finding of the study that we noticed was the fact that the CBG in the concentration of 10 μM, contrary to the lower concentrations, had diminished the accumulation of DAG as well as the content of SFAs and C16:0 within this lipid fraction in H9c2 cells in a lipid overload environment. In parallel, the same concentration of CBG was also found to decrease the level of FA-transporting proteins in cardiomyocytes, i.e., CD36 and FABPpm. Therefore, we may assume that the CBG in the concentration of 10 μM had induced a metabolic transition in H9c2 cardiomyocytes, compared to other examined concentrations, causing a protective reduction of intracellular DAG accumulation, potentially against lipid-induced cellular dysfunction.

**Figure 8:**
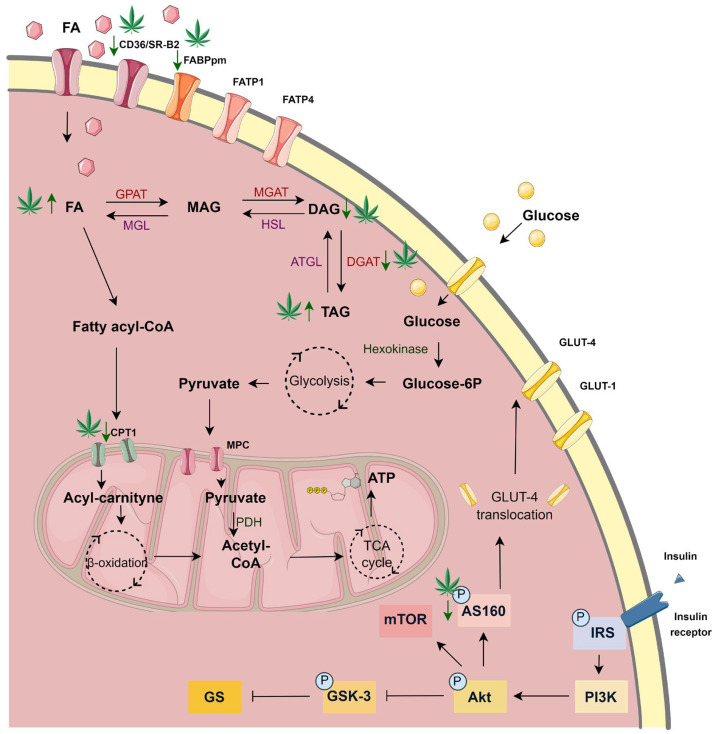
The significant changes in the metabolism of H9c2 cardiomyocytes after 18 h incubation with palmitate (PA) and 10 µM cannabigerol (CBG) in comparison to the PA group. ATGL—adipose triglyceride lipase; ATP—adenosine triphosphate; CD36/SR-B2—fatty acid translocase; CoA—coenzyme A; CPTI—carnitine palmitoyltransferase I; DAG—diacyloglycerol; DGAT—diacylglycerol O-acyltransferase; FA—fatty acid; FABPpm—fatty acid-binding protein; FATP-1—fatty acid transport protein 1; FATP-4—fatty acid transport protein 4; Glucose-6P—glucose 6-phosphate; GLUT-4—glucose transporter type 4; GPAT -glycerol-3-phosphate acyltransferase; HSL—hormone-sensitive lipase; MAG—monoacylglycerol; MGAT—monoacylglycerol O-acyltransferase; MGL—monoacylglycerol lipase; MPC—mitochondrial pyruvate carrier; PDH—pyruvate dehydrogenase; TAG -triacyloglycerol; TCA—tricarboxylic acid.

FAs can enter the cardiomyocytes via simple diffusion or protein-mediated transmembrane transport, and the latter accounts for more than 70% of the total FA uptake by the cardiac cells [32]. CD36 has been proposed as a pivotal regulator of this process, given that CD36-mediated FA uptake contributes to approximately 60% of FA oxidation by the cardiomyocytes, both in low- and high-palmitate conditions [33]. A significant role of this transporter was also confirmed in the study conducted on the CD36 knockout mice, which displayed lower FA uptake rates and altered FA metabolism compared to the wild-type mice [34]. As it was mentioned earlier, in our experiment, only the highest CBG concentration (10 μM) diminished the level of CD36 and consequently the accumulation of lipids in the PA overload conditions (Figure 8), which may be advantageous in the states of FA oversupply (e.g., in obesity) as it protects against the excessive FA uptake into the cardiomyocytes and further lipotoxicity development [35]. In line with these observations, there are many obesity model studies (e.g., isolated rat cardiomyocytes or high-fat diet-fed rodents), where lowering cellular CD36 level exhibits a protective effect against the development of insulin resistance, lipotoxicity, and cardiac dysfunction [36,37,38,39]. Moreover, the level of FABPpm, another FA transporter abundantly expressed in the cardiomyocytes, was also decreased in H9c2 cells as a response to the 18 h incubation with the highest CBG concentration (10 μM). It is not surprising since the researchers suggest that these two transporters interact with each other in FA uptake [40]. It was shown that FABPpm is permanently present at the plasma membrane and that, when there is certain stimulation (e.g., insulin or contractile activity), CD36 relocates to the lipid rafts in the sarcolemma and then binds to FABPpm to assemble the FA transport complex [41]. Nevertheless, decreasing the level of both CD36 and FABPpm in the cardiomyocytes in lipid overload conditions by CBG introduction seems to be an effective way of reducing FA transport into the cells along with their further accumulation. It should be underlined that in our study, lowered levels of CD36 and FABPpm were simultaneous with the reduced content of the DAG fraction in the H9c2 cardiomyocytes. Interestingly, levels of two other protein transporters present in H9c2 cardiomyocytes, i.e., FATP-1 and FATP-4, were increased after incubation with a 10 μM concentration of CBG compared to CD36 and FABPpm. The studies suggest that the role of FATPs in the working heart is less significant than CD36 and FABPpm, as it was demonstrated that the rise in FATP level was not followed by the altered FA uptake [35]. Moreover, FATPs are mainly responsible for the uptake of very long-chain FAs (C22 and more) and not the bulk transport of C16:0 and other abundant long-chain FAs [42]. Nevertheless, the FATP proteins possess enzymatic activity, namely exhibiting intrinsic acyl-CoA synthetase activity, the enzyme that catalyzes the activation of FAs to acyl-CoA esters; therefore, they are involved in FA metabolism [43,44]. In a prior study conducted by our team, it was observed that another phytocannabinoid with similar properties to those of CBG, namely CBD, decreased the level of FATP proteins in the heart and skeletal muscles of rats fed a high-fat diet [45,46]. A similar effect was observed regarding FATP-4 level in our study when the lower concentrations of CBG (2.5 μM and 5 μM) were applied. Noteworthy is the fact that we observed a substantial switch after the treatment of cells with 10 µM CBG and PA toward elevated levels of both FATP-1 and FATP-4. This may represent a compensatory effect to the simultaneously decreased levels of CD36 and FABPpm during lipid oversupply, since facilitated diffusion is not a dominant transport of FAs in H9c2 cardiomyocytes [47]. However, the present study does not explain the exact mechanism responsible for the regulation of the level of FA protein transporters after CBG treatment, which can be considered a limitation and needs further elucidation.

The Figure was partly generated using Servier Medical Art, provided by Servier, licensed under a Creative Commons Attribution 3.0 unported license.

It is known that the accumulation of FAs in the cardiac muscle is linked with the dysfunction of cardiomyocytes, as the FAs taken inside the cells are incorporated into various lipid subclasses, including DAG and ceramide. DAG fraction is considered detrimental when its intracellular level is augmented, and such an imbalance often causes cardiac lipotoxicity [48]. It has been established that the accumulation of DAG was associated with insulin resistance in various tissues, including the liver and skeletal or cardiac muscles, due to the activation of protein kinase C θ (PKCθ), which in turn blocks IRS, inhibits the activation of the Akt pathway and results in lowering the insulin-stimulated glucose uptake to the cells [49,50,51]. This produces an imbalance between FAs and glucose utilization in cardiomyocytes and, in turn, cardiac contractile dysfunction—a common feature of diabetic cardiomyopathy. Importantly, we observed in our study the ability of CBG in the concentration of 10 μM to decrease the content of DAG in H9c2 cardiomyocytes (Figure 8), which was previously increased by the 18 h incubation with PA. This alteration was parallel with the lowered level of DGAT1, an enzyme responsible for DAG conversion into TAG in the same experimental conditions [52], despite the lack of a concomitant decline in TAG content. This may be attributed to the persistent activity of alternative pathways, such as those involving DGAT2, which could have contributed to TAG synthesis [53]. Additionally, in the DAG fraction, we observed a decrease in the content of C16:0 in the cardiomyocytes incubated with PA after the introduction of CBG in all examined concentrations. It is known that the oxidation of SFAs decreases with increasing carbon length; thus, palmitic acid is one of the first FAs directed to the mitochondria for oxidation [54]. CBG in the concentration of 10 μM also decreased the total content of SFAs within the DAG fraction in lipid overload conditions. This reduction primarily resulted from significant changes in the major FA of this fraction, i.e., C16:0. Such modulation may suggest a potential benefit, e.g., by alleviating inflammation linked to toll-like receptor 4 (TLR4) activation induced by C16:0 overload [55]. Furthermore, it has been demonstrated that an increase in the content of C16:0 may impair β-oxidation and the citric acid cycle in primary neonatal cardiomyocytes [56]. Furthermore, DAG also serves as a precursor for endocannabinoids, particularly 2-arachidonoylglycerol (2-AG). 2-AG acts as an agonist of cannabinoid receptors CB1 and CB2 and is primarily metabolized by MAGL. In our experiment, we observed that CBG effectively decreased MAGL levels across all examined concentrations. Simultaneously, the level of another key enzyme that breaks down anandamide, FAAH1, was reduced by the highest CBG concentration. These findings align with a previous study demonstrating CBG’s ability to interact with the endocannabinoid system, including the inhibition of both MAGL and FAAH1 [19]. Inhibitors of MAGL and FAAH1 are considered promising therapeutic agents due to their potential in the modulation of inflammation and oxidative stress, processes frequently associated with lipid metabolism disorders [57,58].

The decreased content of C16:0 and SFAs within the DAG fraction after CBG treatment may indicate its involvement in the beneficial regulation of FAs oxidation. However, an 18 h incubation of the cells may be too short to affect other examined FAs. Rather, it may only impact those that are initially subjected to oxidation. In contrast, the content of MUFAs and PUFAs in the DAG fraction in H9c2 cardiomyocytes in lipid overload conditions was elevated by each examined CBG concentration (MUFAs) or by 2.5 µM and 5 µM CBG (PUFAs). This change occurred due to the increased buildup of certain FAs within those fractions, namely C18:1 and C16:1 for MUFAs as well as C20:4 for PUFAs. Both PUFAs and MUFAs, due to their anti-inflammatory and antioxidant properties, have beneficial effects on cardiovascular disorders by alleviating inflammatory status or inhibiting cardiac fibrosis [59,60]. Not surprisingly, we observed a significantly elevated content of C20:4 in the PL fraction following treatment with PA [61], which was reflected by the elevated levels of COX-1 and COX-2. Both enzymes are involved in the conversion of C20:4 into various bioactive inflammatory lipid mediators, i.e., eicosanoids, including prostaglandins associated with the inflammatory response [62]. Treatment with CBG at the highest concentration tested (10 μM) markedly reduced the levels of key enzymes involved in the arachidonic acid cascade, specifically cPLA2, COX-1, and COX-2, indicating its potential anti-inflammatory properties. This observation is consistent with previous findings demonstrating reduced levels of COX-1 and COX-2 in HT29 cells [62], as well as a decreased expression of COX-1, COX-2, and cPLA2 in rodent models [63,64] after CBG treatment.

As we assumed, the treatment of H9c2 cardiomyocytes with PA resulted in an elevation of all examined lipid fractions. Under these conditions, we observed increased levels of both CPTI and CS, suggesting that the upregulated FA uptake caused both lipid accumulation and an increase in β-oxidation and the Krebs cycle. Furthermore, concerning FFA, TAG, and PL fractions, incubation with different CBG concentrations and PA further augmented the accumulation of the mentioned lipid derivatives. However, at the highest concentration of CBG (10 μM), a shift towards decreased accumulation, in comparison to lower CBG concentrations, was observed across all examined lipid fractions. Moreover, the level of CS was meaningfully increased, while the level of CPTI was decreased by the 10 μM CBG concentration. These findings suggest that CBG may induce a metabolic shift from FA oxidation toward glucose metabolism to achieve energetic balance [65].

However, neither glucose uptake nor GLUT-4 content showed a significant elevation in our study. We speculate that the 18 h incubation period may have been insufficient to induce significant changes in glucose uptake. In the heart, the content of TAG under physiological conditions is low, and an increase in this lipid derivative storage occurs in certain conditions, e.g., FA overload [66], as was also confirmed in our study and observed as an elevation in the SFA pool. Another explanation of the increased intramyocardial content of FFA, TAG, and PL fractions after CBG application (2.5 µM and 5 µM) in lipid overload conditions can be attributed to the membrane content of FA transporters. Typically, lipid overload conditions stimulate the translocation of FA carrier proteins, primarily CD36, from intracellular pools to the cardiomyocyte membrane. This translocation facilitates an increased influx of FA into the cell, which can be subsequently oxidized or accumulated in different lipid pools [67,68]. Because we did not observe significant changes in the level of CD36 and FABPpm following the administration of 2.5 µM and 5 µM CBG, we hypothesize that the lipid overload conditions led to the permanent upregulation of membrane level of this transporter, which is an early and rapid process, thereby promoting the accumulation of FAs within the FFA, TAG, and PL pools at the onset of insulin resistance, as it was indicated in different obesity studies [36,69,70,71]. The mechanism responsible for keeping CD36 in intracellular depots is related to endosomal acidification [72]. Interestingly, it was shown that long-chain FAs (C16:0 and C18:0) in vitro or in vivo induce a loss of endosomal acidification by v-ATPase inhibition and consequently the translocation of CD36 to the cell surface, which in the long term leads to insulin resistance [36,73]. This is in line with our results, since we observed a significant increase in both palmitate and oleate in each examined lipid fraction after exposing cardiomyocytes to palmitate. Notably, at a higher CBG concentration of 10 µM, there was a marked reduction in the levels of the above-mentioned FA transporters, which corresponded with a trend toward decreased accumulation of the FFA, TAG, and PL fractions. The precise mechanism by which CBG influences these processes requires further investigation, particularly concerning its effects on factors that regulate the levels and translocation of CD36, such as 5′AMP-activated protein kinase (AMPK) or peroxisome proliferator-activated receptors (PPARs). Additionally, CBG has been demonstrated to lack dose-responsiveness in other recent studies, instead exhibiting variability in its effective concentrations [74,75]. Our finding of differential effects between low and high doses of CBG is consistent with the results reported by Aljobaily and colleagues. Their study demonstrated that a low dose of CBG reduced fibrosis and inflammation in liver tissue affected by non-alcoholic steatohepatitis in mice, while the high dose produced the opposite effect [76]. Furthermore, incubation with PA in the presence of all examined concentrations of CBG resulted in a further elevation of the SFA content within the TAG fraction. Nevertheless, researchers suggest that the accumulation of excessive FAs into the TAG pool may serve as a protective mechanism against lipotoxicity as it protects the cell from palmitate-induced oxidative stress and apoptosis [77,78]. Additionally, in H9c2 cells, we observed a rise in MUFA and PUFA content after simultaneous PA and CBG administration in all the above-mentioned lipid fractions. As mentioned above, both lipid fractions have beneficial effects on cardiovascular disorders. Furthermore, the observed rise may be attributed to the ability of CBG to influence the activity of enzymes responsible for PUFA metabolism, namely COX-1 and COX-2, which levels were decreased in our study after concomitant PA and CBG treatment [62,79] We assume that the highest CBG concentration used in our experiment modulated the metabolism of H9c2 cardiomyocytes, thereby influencing lipid storage and composition. This hypothesis is supported by observed reduction in the levels of CD36 and FABPpm as well as the content of lipid fractions, especially a decrease in the accumulation of DAG and C16:0 within this fraction. The lipophilic phytocannabinoids, including CBG, have already been shown to modulate cellular metabolism, especially in cancer cells [80,81]. Furthermore, another research conducted on melanocytes introduced the ability of CBG to influence phospholipid metabolism [82].

Regarding the second important energetic substrate of the heart, namely glucose, we confirmed that PA and insulin treatment caused a decrease in the rate of glucose uptake. However, in our experiment, we did not find a significant influence of the CBG on that transport. The insulin-sensitive GLUT-4 level was pronouncedly increased by the treatment with insulin alone and diminished by incubation with PA and insulin, as well as with CBG and insulin. In a recent study performed on rodents with an acute cachectic phenotype induced by cisplatin, CBG (120 mg/kg) partially normalized the metabolic alterations that were initially induced by cisplatin treatment, including changes in glucose and lipid metabolism. In the same study, CBG was also found to influence the phosphorylation and thereby the activation of Akt, previously inhibited by cisplatin [83]. Additionally, the research conducted on a rat model of insulin resistance induced by a high-fat and high-sucrose diet indicated that CBG (30 mg/kg) could improve liver insulin sensitivity by heightening the phosphorylation of Akt and lessening the phosphorylation of GSK-3B [84]. In our study, the phosphorylation of GSK-3, IRS, and Akt was increased after treatment with CBG and insulin. We also observed a trend towards increasing the phosphorylation of the above-mentioned proteins after concomitant treatment with PA, CBG, and insulin, compared to the PA and insulin group. We speculate that the observed results may be connected to the shorter treatment period utilized in our experiment, which lasted 18 h. This stands in contrast to the treatment durations of 3 days and 14 days reported in the aforementioned studies. Moreover, the pAS160/AS160 ratio was increased after concomitant treatment with PA, CBG, and insulin. Interestingly, our findings are supported by a previous study, which reported no statistically significant reduction in the phosphorylation of GSK-3, following the administration of a high-fat diet alone or with sucrose in rodent models [84].

### Study Limitations and Future Directions

It should be kept in mind that our study has several limitations. There is a need for validation of our findings in primary cardiomyocytes, as well as other cell lines (e.g., adipocytes and hepatocytes), and, importantly, in animal models of obesity. Moreover, the lysates of the H9c2 cardiomyocytes contain a relatively small amount of protein compared to tissue homogenates. Consequently, this may result in a reduced band intensity for certain proteins, even though a highly sensitive detection substrate was used. Therefore, it prompts the translation of the study design to an animal model and tissue homogenates. Moreover, for a precise determination of the mechanism of CBG action concerning FA and glucose transport, future studies should implement specific inhibitors (e.g., CD36 inhibitor) or gene silencing, as well as the evaluation of the translocation and recycling of FA and glucose transporters between plasma membranes and endosomes. The protein level assessed by Western blot analysis may be further supported by the assessment of the corresponding gene expression in future studies. Importantly, future studies should also aim to replicate these results in human-derived cells to assess the physiological significance of a potential therapy with CBG.

## 5. Conclusions

In our research, we presented evidence suggesting that CBG treatment, especially in higher concentrations (10 µM), may offer substantial benefits in the states associated with excessive lipid availability, which was demonstrated in the H9c2 cell model. The results obtained in our experiment suggest that CBG possesses the ability to alter the metabolism of H9c2 cells by influencing FA storage and utilization while also attenuating the inflammatory pathways activated in a high-lipid environment. These findings indicate that CBG may represent a promising therapeutic candidate for further investigation concerning lipotoxicity and insulin resistance development. Moreover, CBG is predisposed to be a metabolic modulator by altering the levels and cellular location of CD36, a major regulator of myocardial lipid metabolism and a therapeutic target for metabolic disturbances.

## Figures and Tables

**Table 1:** Individual fatty acid composition of the free fatty acid (FFA) fraction in H9c2 cardiomyocytes after 18 h incubation with palmitate (PA) and/or cannabigerol (CBG). The data are expressed in nmol/mg of protein as mean values ± SD and are based on six independent determinations in each group.

FFA	Control	CBG 2.5	CBG 5	CBG 10	PA	PA + CBG 2.5	PA + CBG 5	PA + CBG 10
C14:0	1.74 ± 0.41	1.72 ± 0.32	1.44 ± 0.18	1.51 ± 0.14	1.71 ± 0.35	3.77 ± 0.49 ^ab^	3.88 ± 0.49 ^ab^	3.13 ± 0.18 ^abf^
C16:0	11.16 ± 1.71	9.26 ± 1.04	8.76 ± 1.34	9.00 ± 1.15	29.54 ± 4.18 ^a^	35.66 ± 1.73 ^ab^	46.72 ± 2.88 ^abe^	40.45 ± 5.03 ^abf^
C16:1	0.68 ± 0.16	0.45 ± 0.09	0.40 ± 0.05	0.56 ± 0.11	0.99 ± 0.20 ^a^	0.80 ± 0.13	1.14 ± 0.23 ^ae^	0.94 ± 0.14
C18:0	8.97 ± 0.95	9.39 ± 0.61	10.45 ± 1.69	9.59 ± 1.39	12.45 ± 2.67 ^a^	13.11 ± 1.85	15.90 ± 0.99 ^ab^	18.29 ± 2.31 ^abe^
C18:1	1.92 ± 0.27	1.50 ± 0.14	1.58 ± 0.13	1.49 ± 0.13	1.45 ± 0.20	2.00 ± 0.44 ^b^	2.25 ± 0.46 ^b^	2.19 ± 0.24 ^b^
C18:2	0.77 ± 0.18	0.58 ± 0.06	0.54 ± 0.14	0.70 ± 0.12	0.81 ± 0.10	1.00 ± 0.28	1.30 ± 0.16 ^ab^	1.15 ± 0.18 ^ab^
C20:0	0.89 ± 0.24	0.38 ± 0.15 ^a^	0.31 ± 0.07 ^a^	0.73 ± 0.13 ^cd^	0.66 ± 0.13	0.76 ± 0.11	0.91 ± 0.11	0.89 ± 0.11
C18:3	0.54 ± 0.08	0.36 ± 0.11	0.51 ± 0.15	0.66 ± 0.17	1.08 ± 0.29 ^a^	0.98 ± 0.26 ^a^	1.38 ± 0.29 ^abe^	1.11 ± 0.11 ^a^
C22:0	0.57 ± 0.11	0.40 ± 0.10	0.37 ± 0.06	0.75 ± 0.09 ^cd^	0.60 ± 0.07	0.84 ± 0.19 ^ab^	1.14 ± 0.19 ^abe^	0.78 ± 0.09 ^f^
C20:4	0.54 ± 0.09	0.40 ± 0.10	0.45 ± 0.04	0.61 ± 0.19	0.71 ± 0.13	1.09 ± 0.29 ^ab^	1.66 ± 0.36 ^abe^	1.07 ± 0.20 ^af^
C24:0	0.57 ± 0.06	0.37 ± 0.11 ^a^	0.36 ± 0.06 ^a^	0.53 ± 0.14	0.64 ± 0.14	0 ± 0.00 ^ab^	0 ± 0.00 ^ab^	0.73 ± 0.13
SFAs	29.89 ± 2.83	27.81 ± 2.14	25.37 ± 1.82	27.84 ± 2.72	44.56 ± 5.04 ^a^	55.22 ± 3.75 ^ab^	50.77 ± 3.76 ^a^	67.37 ± 6.73 ^abef^
MUFAs	3.08 ± 0.48	2.45 ± 0.12	2.66 ± 0.42	2.53 ± 0.46	2.36 ± 0.32 ^a^	2.65 ± 0.13	2.31 ± 0.33 ^a^	3.32 ± 0.32 ^bf^
PUFAs	2.30 ± 0.17	1.65 ± 0.20	1.81 ± 0.42	2.69 ± 0.43 ^cd^	2.70 ± 0.28	2.94 ± 0.41	3.22 ± 0.41 ^a^	3.53 ± 0.34 ^ab^

^a^ *p*  <  0.05 indicates a significant difference: the control group in comparison to the examined group. ^b^ *p*  <  0.05 indicates a significant difference: the PA group in comparison to the examined group. ^c^ *p*  <  0.05 indicates a significant difference: the CBG 2.5 group in comparison to the examined group. ^d^ *p*  <  0.05 indicates a significant difference: the CBG 5 group in comparison to the examined group. ^e^ *p*  <  0.05 indicates a significant difference: the PA + CBG 2.5 group in comparison to the examined group. ^f^ *p*  <  0.05 indicates a significant difference: the PA + CBG 5 group in comparison to the examined group.

**Table 2:** Individual fatty acid composition of the diacyloglycerol (DAG) fraction in H9c2 cardiomyocytes after 18 h incubation with palmitate (PA) and/or cannabigerol (CBG). The data are expressed in nmol/mg of protein as mean values ± SD and are based on six independent determinations in each group.

DAG	Control	CBG 2.5	CBG 5	CBG 10	PA	PA + CBG 2.5	PA + CBG 5	PA + CBG 10
C14:0	2.82 ± 0.38	2.85 ± 0.55	2.51 ± 0.26	1.61 ± 0.47 ^ac^	3.03 ± 0.72	4.35 ± 0.58 ^ab^	3.88 ± 0.73 ^a^	3.18 ± 0.23 ^e^
C16:0	11.44 ± 0.59	10.04 ± 1.46	9.30 ± 0.89	11.64 ± 1.93	97.04 ± 5.94 ^a^	67.63 ± 9.69 ^ab^	61.94 ± 1.66 ^ab^	58.05 ± 4.72 ^abe^
C16:1	0.33 ± 0.06	0.46 ± 0.09	0.42 ± 0.05	0.61 ± 0.32	0.62 ± 0.18	0.45 ± 0.10	0.85 ± 0.29 ^ae^	1.08 ± 0.14 ^abe^
C18:0	8.53 ± 0.96	8.58 ± 1.30	7.09 ± 0.80	8.53 ± 1.23	12.69 ± 2.24 ^a^	13.34 ± 1.18 ^a^	14.84 ± 2.34 ^a^	10.37 ± 2.37 ^f^
C18:1	2.31 ± 0.42	2.76 ± 0.43	1.39 ± 0.27	4.22 ± 1.27	3.67 ± 0.65	6.05 ± 1.77 ^a^	5.63 ± 1.85 ^a^	4.99 ± 2.69
C18:2	2.09 ± 0.53	2.74 ± 0.80	1.16 ± 0.32	5.03 ± 1.50 ^ad^	4.77 ± 1.63	5.14 ± 1.68 ^a^	4.46 ± 1.95	3.01 ± 1.37
C20:0	0.34 ± 0.09	0.28 ± 0.04	0.32 ± 0.11	0.29 ± 0.04	0.40 ± 0.11	4.84 ± 0.88 ^ab^	6.00 ± 0.90 ^abe^	3.65 ± 0.50 ^abef^
C18:3	0.44 ± 0.10	0.60 ± 0.24	0.58 ± 0.39	0.59 ± 0.15	0.82 ± 0.27	0.47 ± 0.15	0.67 ± 0.14	0.99 ± 0.16 ^ae^
C22:0	0.38 ± 0.13	0.41 ± 0.11	0.41 ± 0.17	0.46 ± 0.07	0.76 ± 0.09	7.22 ± 1.22 ^ab^	9.63 ± 1.33 ^abe^	5.57 ± 0.67 ^abef^
C20:4	0.63 ± 0.13	0.64 ± 0.10	0.59 ± 0.10	0.46 ± 0.09	0.60 ± 0.14	1.69 ± 0.50 ^ab^	1.13 ± 0.20	1.46 ± 0.62 ^a^
C24:0	0.42 ± 0.13	0.42 ± 0.14	0.33 ± 0.11	0.41 ± 0.06	0.62 ± 0.13	4.24 ± 0.75 ^ab^	4.77 ± 1.03 ^ab^	3.40 ± 0.33 ^abf^
SFAs	24.87 ± 3.07	22.84 ± 4.11	19.86 ± 1.92	22.70 ± 3.43	114.59 ± 6.73 ^a^	106.98 ± 15.45 ^a^	102.50 ± 7.96 ^a^	83.13 ± 5.35 ^abef^
MUFAs	2.71 ± 0.31	3.21 ± 0.47	1.81 ± 0.25	4.83 ± 1.46	4.23 ± 0.55	6.49 ± 1.71 ^a^	5.89 ± 2.10 ^a^	6.63 ± 2.68 ^a^
PUFAs	3.26 ± 0.46	4.11 ± 0.98	2.33 ± 0.44	5.99 ± 1.41	6.25 ± 1.75	9.55 ± 3.85 ^a^	7.65 ± 2.36 ^a^	5.90 ± 1.86

^a^ *p*  <  0.05 indicates a significant difference: the control group in comparison to the examined group. ^b^ *p*  <  0.05 indicates a significant difference: the PA group in comparison to the examined group. ^c^ *p*  <  0.05 indicates a significant difference: the CBG 2.5 group in comparison to the examined group. ^d^ *p*  <  0.05 indicates a significant difference: the CBG 5 group in comparison to the examined group. ^e^ *p*  <  0.05 indicates a significant difference: the PA + CBG 2.5 group in comparison to the examined group. ^f^ *p*  <  0.05 indicates a significant difference: the PA + CBG 5 group in comparison to the examined group.

**Table 3:** Individual fatty acid composition of the triacyloglycerol (TAG) fraction in H9c2 cardiomyocytes after 18 h incubation with palmitate (PA) and/or cannabigerol (CBG). The data are expressed in nmol/mg of protein as mean values ± SD and are based on six independent determinations in each group.

TAG	Control	CBG 2.5	CBG 5	CBG 10	PA	PA + CBG 2.5	PA + CBG 5	PA + CBG 10
C14:0	3.86 ± 0.67	3.88 ± 0.45	4.58 ± 0.31	3.26 ± 0.38 ^d^	4.10 ± 0.76	6.52 ± 0.56 ^ab^	7.44 ± 0.41 ^ab^	5.81 ± 0.61 ^abf^
C16:0	10.54 ± 1.72	19.97 ± 2.87	26.25 ± 2.58	20.50 ± 2.98	208.14 ± 12.25 ^a^	359.80 ± 32.72 ^ab^	414.68 ± 30.16 ^abe^	356.60 ± 26.73 ^abf^
C16:1	3.88 ± 0.58	1.87 ± 0.35 ^a^	2.15 ± 0.37 ^a^	2.61 ± 0.35 ^a^	2.49 ± 0.65 ^a^	2.32 ± 0.23 ^a^	5.75 ± 1.01 ^abe^	3.23 ± 0.35 ^f^
C18:0	6.60 ± 2.44	10.59 ± 1.31 ^a^	13.87 ± 2.00 ^a^	8.65 ± 0.73 ^d^	10.51 ± 1.66 ^a^	14.13 ± 1.68 ^a^	16.18 ± 2.76 ^ab^	13.50 ± 1.90 ^a^
C18:1	9.62 ± 2.76	19.90 ± 4.23 ^a^	17.79 ± 3.26 ^a^	10.45 ± 2.11 ^cd^	6.19 ± 1.64	14.86 ± 3.10 ^b^	10.19 ± 1.56	7.48 ± 2.20 ^e^
C18:2	6.36 ± 1.68	22.76 ± 3.66 ^a^	14.02 ± 5.12 ^ac^	5.14 ± 1.38 ^cd^	5.54 ± 2.07	12.75 ± 3.83 ^ab^	7.49 ± 1.76	3.71 ± 1.71 ^e^
C20:0	1.50 ± 0.33	1.96 ± 0.34	1.98 ± 0.34	2.48 ± 0.37 ^a^	3.25 ± 0.57 ^a^	2.67 ± 0.50 ^a^	2.27 ± 0.41 ^b^	1.70 ± 0.47 ^be^
C18:3	3.66 ± 0.36	1.71 ± 0.40 ^a^	1.86 ± 0.24 ^a^	0.94 ± 0.28 ^acd^	1.02 ± 0.25 ^a^	1.17 ± 0.39 ^a^	1.69 ± 0.38 ^ab^	1.21 ± 0.32 ^a^
C22:0	2.43 ± 0.09	1.70 ± 0.28	1.66 ± 0.50	1.42 ± 0.31 ^a^	2.06 ± 0.38	2.19 ± 0.60	1.90 ± 0.66	2.22 ± 0.31
C20:4	2.31 ± 0.18	1.32 ± 0.33 ^a^	2.00 ± 0.51 ^c^	1.20 ± 0.30 ^ad^	1.38 ± 0.38 ^a^	1.29 ± 0.38 ^a^	1.74 ± 0.39	1.40 ± 0.33 ^a^
SFAs	24.91 ± 3.93	37.89 ± 4.44	52.09 ± 7.71	35.99 ± 5.80	228.74 ± 12.62 ^a^	389.65 ± 35.07 ^ab^	445.77 ± 25.81 ^abe^	380.30 ± 28.66 ^abf^
MUFAs	13.12 ± 3.09	21.74 ± 4.59 ^a^	19.95 ± 3.22 ^a^	12.91 ± 2.51 ^cd^	8.67 ± 2.28	15.22 ± 3.20	15.31 ± 2.52	11.46 ± 2.86
PUFAs	12.39 ± 1.66	25.37 ± 3.90 ^a^	17.97 ± 5.12	7.20 ± 1.73 ^cd^	8.11 ± 2.71	16.49 ± 5.20 ^b^	12.89 ± 5.11	6.97 ± 2.62 ^e^

^a^ *p*  <  0.05 indicates a significant difference: the control group in comparison to the examined group. ^b^ *p*  <  0.05 indicates a significant difference: the PA group in comparison to the examined group. ^c^ *p*  <  0.05 indicates a significant difference: the CBG 2.5 group in comparison to the examined group. ^d^ *p*  <  0.05 indicates a significant difference: the CBG 5 group in comparison to the examined group. ^e^ *p*  <  0.05 indicates a significant difference: the PA + CBG 2.5 group in comparison to the examined group. ^f^ *p*  <  0.05 indicates a significant difference: the PA + CBG 5 group in comparison to the examined group.

**Table 4:** Individual fatty acid composition of the phospholipid (PL) fraction in H9c2 cardiomyocytes after 18 h incubation with palmitate (PA) and/or cannabigerol (CBG). The data are expressed in nmol/mg of protein as mean values ± SD and are based on six independent determinations in each group.

PL	Control	CBG 2.5	CBG 5	CBG 10	PA	PA + CBG 2.5	PA + CBG 5	PA + CBG 10
C14:0	9.59 ± 0.60	14.79 ± 3.39	16.18 ± 3.15	13.04 ± 1.87	16.38 ± 3.47	30.16 ± 6.34 ^ab^	35.30 ± 6.80 ^ab^	22.55 ± 4.89 ^a^
C16:0	77.18 ± 5.36	87.24 ± 7.70	108.67 ± 11.54	90.00 ± 5.27	460.48 ± 31.51 ^a^	531.37 ± 72.55 ^ab^	570.84 ± 27.45 ^ab^	523.59 ± 9.43 ^a^
C16:1	5.38 ± 1.43	21.17 ± 2.45 ^a^	20.80 ± 2.12 ^a^	16.84 ± 1.80 ^acd^	15.28 ± 2.48 ^a^	13.03 ± 1.79 ^a^	20.90 ± 1.80 ^abe^	13.58 ± 1.63 ^af^
C18:0	75.41 ± 6.03	76.85 ± 5.78	91.73 ± 12.17 ^a^	85.38 ± 5.87	75.12 ± 3.46	87.32 ± 12.72	91.59 ± 7.01 ^ab^	76.72 ± 6.47
C18:1	37.27 ± 2.21	52.51 ± 9.62 ^a^	48.90 ± 4.52	36.12 ± 4.09 ^cd^	35.01 ± 2.52	36.80 ± 8.47	39.05 ± 5.54	31.70 ± 3.96
C18:2	16.86 ± 2.33	29.27 ± 5.48 ^a^	23.97 ± 1.27	20.27 ± 3.76	20.56 ± 6.35	21.04 ± 5.35	35.18 ± 8.79 ^abe^	18.69 ± 6.65 ^f^
C20:0	2.05 ± 0.24	4.48 ± 1.49	3.98 ± 0.48	4.97 ± 0.45 ^a^	5.73 ± 1.90 ^a^	7.77 ± 1.81 ^a^	9.07 ± 2.13 ^ab^	7.05 ± 1.63 ^a^
C18:3	3.59 ± 1.60	8.61 ± 1.98	4.97 ± 0.41	8.80 ± 2.65	8.57 ± 2.20	11.67 ± 1.37 ^a^	18.61 ± 4.92 ^abe^	9.69 ± 2.25 ^af^
C22:0	3.81 ± 0.35	7.85 ± 0.99	8.79 ± 1.13	8.37 ± 1.92	10.94 ± 2.28 ^a^	14.83 ± 3.29 ^a^	25.22 ± 7.02 ^abe^	10.88 ± 2.31 ^af^
C20:4	52.88 ± 5.02	64.87 ± 4.89 ^a^	71.32 ± 5.83 ^a^	64.97 ± 5.09 ^a^	65.74 ± 6.02 ^a^	56.66 ± 9.56	65.97 ± 5.16 ^a^	55.70 ± 3.31
C20:5	8.62 ± 0.74	16.03 ± 1.47 ^a^	16.73 ± 2.68 ^a^	14.85 ± 2.15 ^a^	17.30 ± 1.97 ^a^	15.99 a ± 2.63	20.06 ± 2.04 ^a^	15.50 ± 4.33 ^a^
C22:6	5.89 ± 1.05	12.58 ± 2.15 ^a^	15.37 ± 3.02 ^a^	10.07 ± 2.05	13.73 ± 1.60 ^a^	12.90 ± 3.98 ^a^	23.34 ± 1.60 ^abe^	21.14 ± 5.01 ^abe^
SFAs	168.03 ± 0.94	202.34 ± 21.58	219.44 ± 15.43	207.84 ± 19.20	600.55 ± 47.92 ^a^	690.76 ± 83.47 ^ab^	729.25 ± 28.41 ^ab^	685.80 ± 47.30 ^ab^
MUFAs	43.87 ± 3.63	76.39 ± 9.69 ^a^	70.41 ± 5.41 ^a^	56.50 ± 8.85 ^acd^	51.47 ± 5.22	49.83 ± 9.24	59.96 ± 5.42 ^a^	45.28 ± 3.55 ^f^
PUFAs	85.91 ± 5.39	129.63 ± 15.81 ^a^	128.37 ± 13.01 ^a^	116.05 ± 15.82	130.7 ± 12.57 ^a^	115.34 ± 20.71	169.60 ± 14.57 ^abe^	116.50 ± 19.23 ^f^

^a^ *p*  <  0.05 indicates a significant difference: the control group in comparison to the examined group. ^b^ *p*  <  0.05 indicates a significant difference: the PA group in comparison to the examined group. ^c^ *p*  <  0.05 indicates a significant difference: the CBG 2.5 group in comparison to the examined group. ^d^ *p*  <  0.05 indicates a significant difference: the CBG 5 group in comparison to the examined group. ^e^ *p*  <  0.05 indicates a significant difference: the PA + CBG 2.5 group in comparison to the examined group. ^f^ *p*  <  0.05 indicates a significant difference: the PA + CBG 5 group in comparison to the examined group.

## Data Availability

The original contributions presented in this study are included in the article/Supplementary Materials. Further inquiries can be directed to the corresponding author.

## Supplementary Materials

The following supporting information can be downloaded at: https://www.mdpi.com/article/10.3390/cells14130998/s1: Supplementary Material contains representative images of uncropped, untouched, full original Western blot images. Moreover, Supplementary Materials contains results from the preliminary studies with insulin (Figure S1) and PA (Figure S2) as well as cell count reports.
