# Supplementary material for: Short-Term Incubation of H9c2 Cardiomyocytes with Cannabigerol Attenuates Diacylglycerol Accumulation in Lipid Overload Conditions
Source: Cells. 2025 Jun 30;14(13):998. doi: 10.3390/cells14130998 (PMC12249120; doi:10.3390/cells14130998)

Cell Count Report

• File name

b

• Date

03 Jun., 2023 11:12

• Cell count results

Total cell concentration: 6.95 x 10^6 cells/mL

Live cell concentration: 4.48 x 10^6 cells/mL

Dead cell concentration: 2.47 x 10^6 cells/mL

Viability: 64.5 %

Average cell size: 15.3 μm

Total cell number: 1474

Live cell number: 950

Dead cell number: 524

• Protocol

Protocol name: DEFAULT

Dilution factor: 2

Min. cell size: 3 μm

Max. cell size: 60 μm

Size gating: 3 ~ 60 μm

Noise reduction: 5

Live cell sensitivity: 1

Roundness: 60 %

Declustering level: Medium

Focusing method: Autofocus

Staining option: With TB

Counting option: Auto exposure(0x0251)

Cell Images (Average intensity: 157)

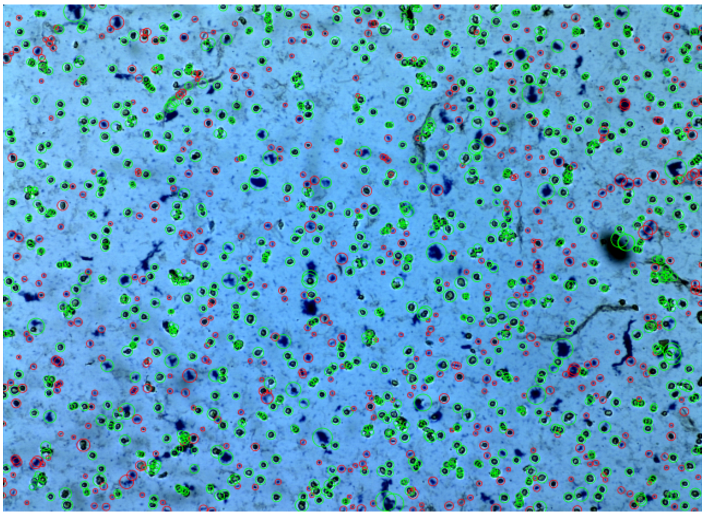

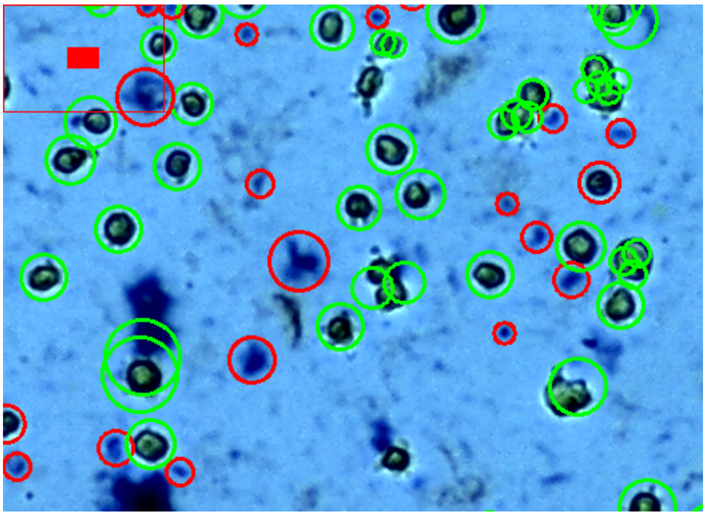

Cell Count Report

• Cell size distribution by cell number

Total Cells	Live Cells	Dead Cells

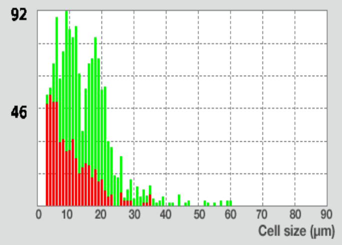

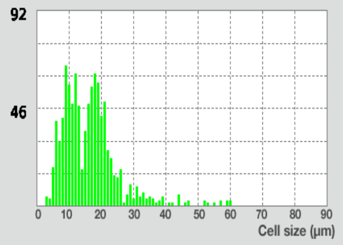

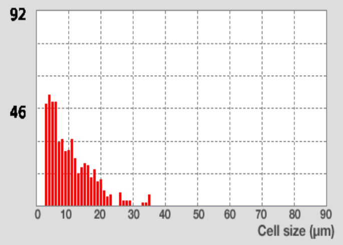

• Cell size distribution by cell concentration

Total Cells	Live Cells	Dead Cells

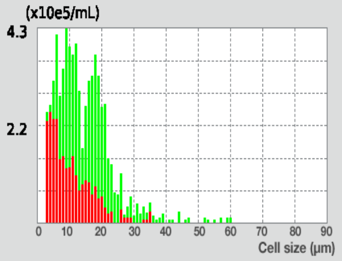

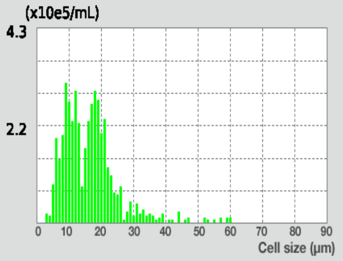

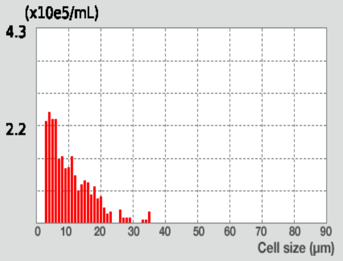

• Cell cluster map

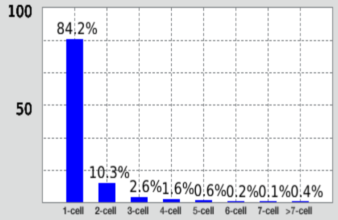

Supplement: Supplementary file 1 [file cells-14-00998-s001.zip › cells-3659124-supplementary/cells count reports/2. CBG 2.5.pdf]
