# Supplementary material for: Short-Term Incubation of H9c2 Cardiomyocytes with Cannabigerol Attenuates Diacylglycerol Accumulation in Lipid Overload Conditions
Source: Cells. 2025 Jun 30;14(13):998. doi: 10.3390/cells14130998 (PMC12249120; doi:10.3390/cells14130998)

# Cell Count Report



## File name

f

## Date

03 Jun., 2023 12:22

## Cell count results

Total cell concentration: 7.00 x $10^6$ cells/mL

Live cell concentration: 5.72 x $10^6$ cells/mL

Dead cell concentration: 1.29 x $10^6$ cells/mL

Viability: 81.6 %

Average cell size: 15.7 μm

Total cell number: 1485

Live cell number: 1212

Dead cell number: 273

## Protocol

Protocol name: DEFAULT

Dilution factor: 2

Min. cell size: 3 μm

Max. cell size: 60 μm

Size gating: 3 ~ 60 μm

Noise reduction: 5

Live cell sensitivity: 1

Roundness: 60 %

Declustering level: Medium

Focusing method: Autofocus

Staining option: With TB

Counting option: Auto exposure(0x022F)

## Cell Images (Average intensity: 153)

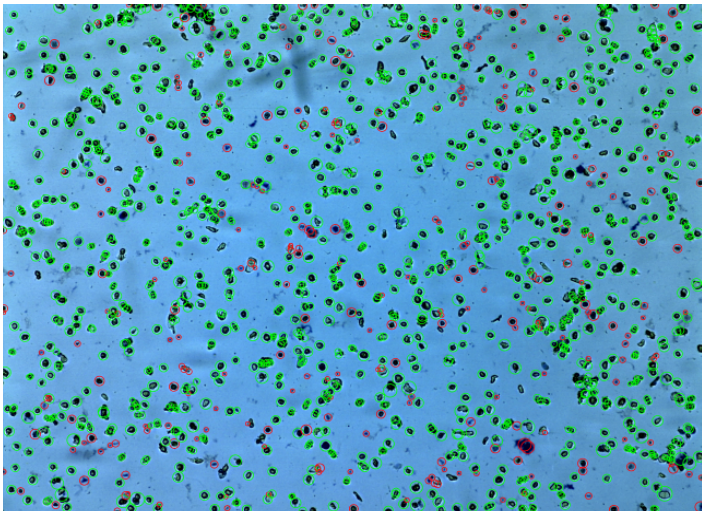

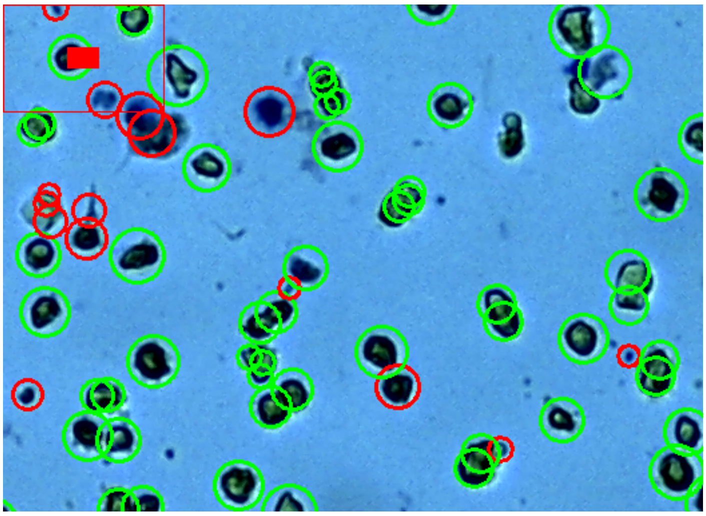

# Cell Count Report



## • Cell size distribution by cell number

| Total Cells | Live Cells | Dead Cells |
| --- | --- | --- |

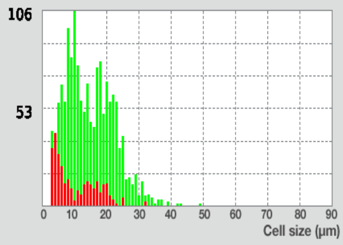


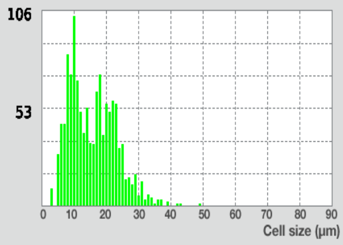


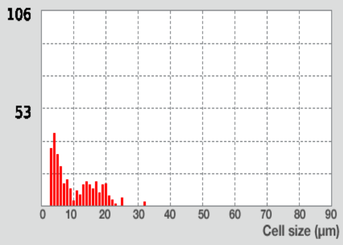


## • Cell size distribution by cell concentration

| Total Cells | Live Cells | Dead Cells |
| --- | --- | --- |

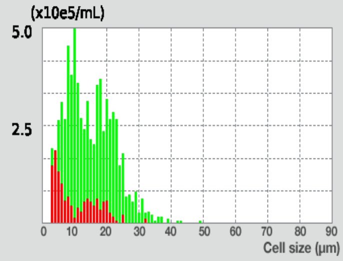


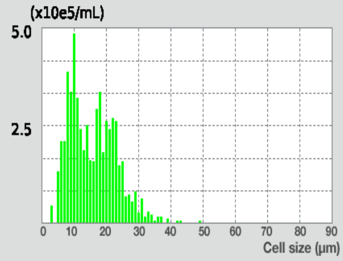


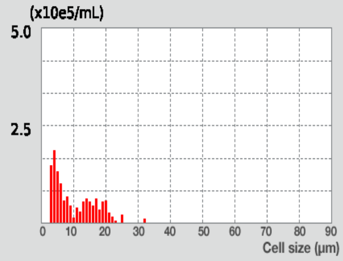


## • Cell cluster map

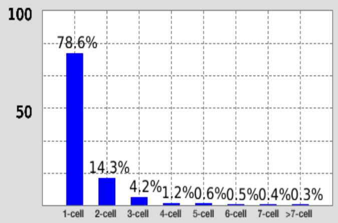

Supplement: Supplementary file 1 [file cells-14-00998-s001.zip › cells-3659124-supplementary/cells count reports/6. PA+CBG 2.5.pdf]
