# Supplementary material for: Short-Term Incubation of H9c2 Cardiomyocytes with Cannabigerol Attenuates Diacylglycerol Accumulation in Lipid Overload Conditions
Source: Cells. 2025 Jun 30;14(13):998. doi: 10.3390/cells14130998 (PMC12249120; doi:10.3390/cells14130998)
Supplement: Supplementary file 1 [file cells-14-00998-s001.zip › cells-3659124-supplementary/preliminary studies-insulin/Figure 1S.pdf]

Figure 1S. The levels of phosphorylated protein kinase B - pAkt (Ser473) (A) and phosphorylated insulin receptor substrate 1 - pIRS1(Ser307) (B) in H9c2 cardiomyocytes after incubation with insulin (100 nM) for 10, 20, 30, and 45 minutes. The levels of the abovementioned proteins are presented as percentage differences compared to the control group which was set as 100%. The data are expressed as mean values ± SD and are based on three independent determinations in each group; $^{a}p < 0.05$ indicates a significant difference: the control group in comparison to the examined group.
