# Supplementary material for: Short-Term Incubation of H9c2 Cardiomyocytes with Cannabigerol Attenuates Diacylglycerol Accumulation in Lipid Overload Conditions
Source: Cells. 2025 Jun 30;14(13):998. doi: 10.3390/cells14130998 (PMC12249120; doi:10.3390/cells14130998)
Supplement: Supplementary file 1 [file cells-14-00998-s001.zip › cells-3659124-supplementary/preliminary studies-insulin/Western Blott.pdf]

pAkt (Ser473)

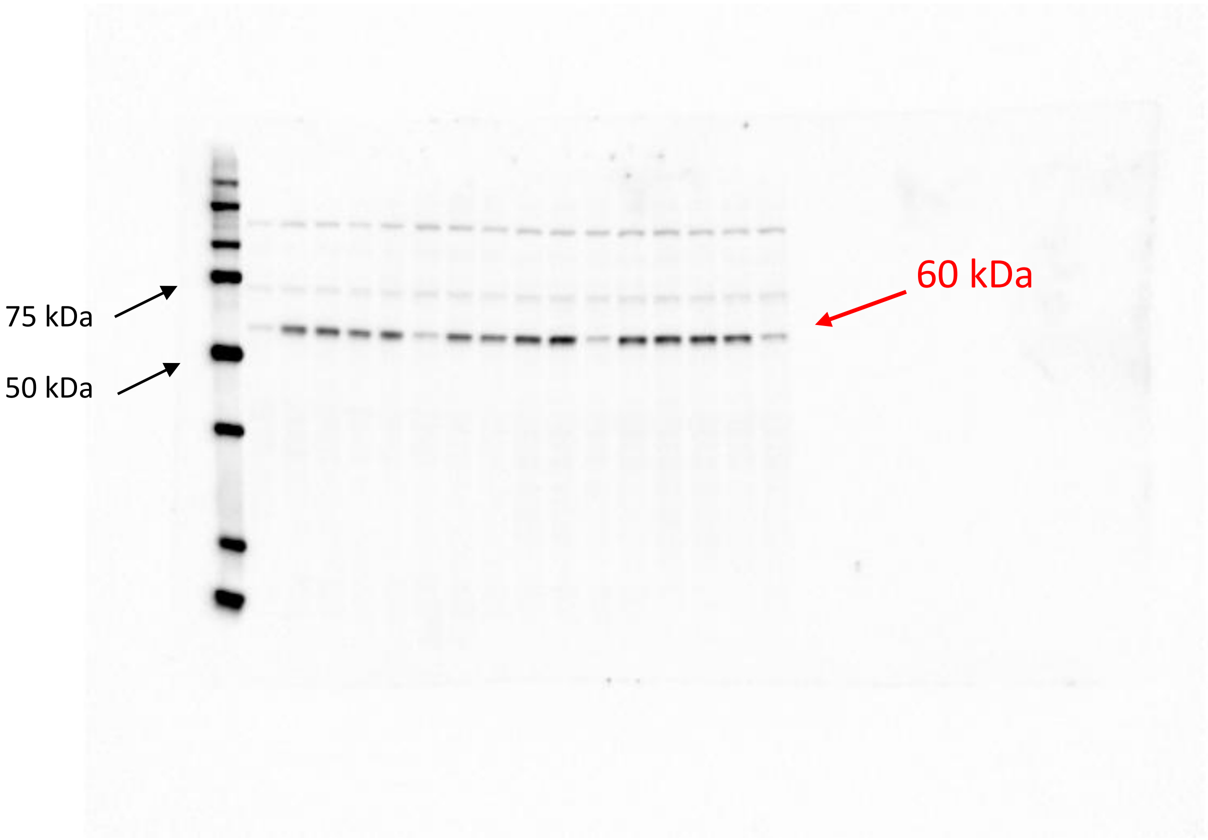

Total protein

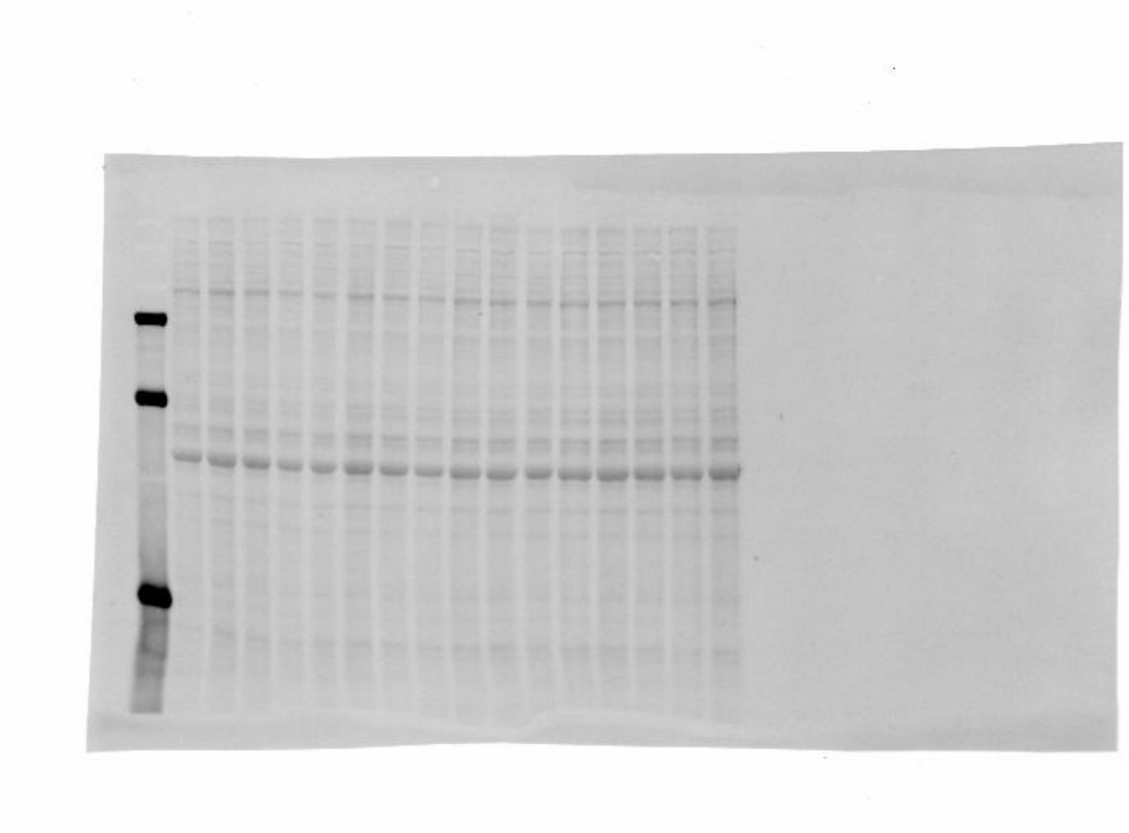

Loading order in all proteins: Control, 10min, 20min, 30min, 45min

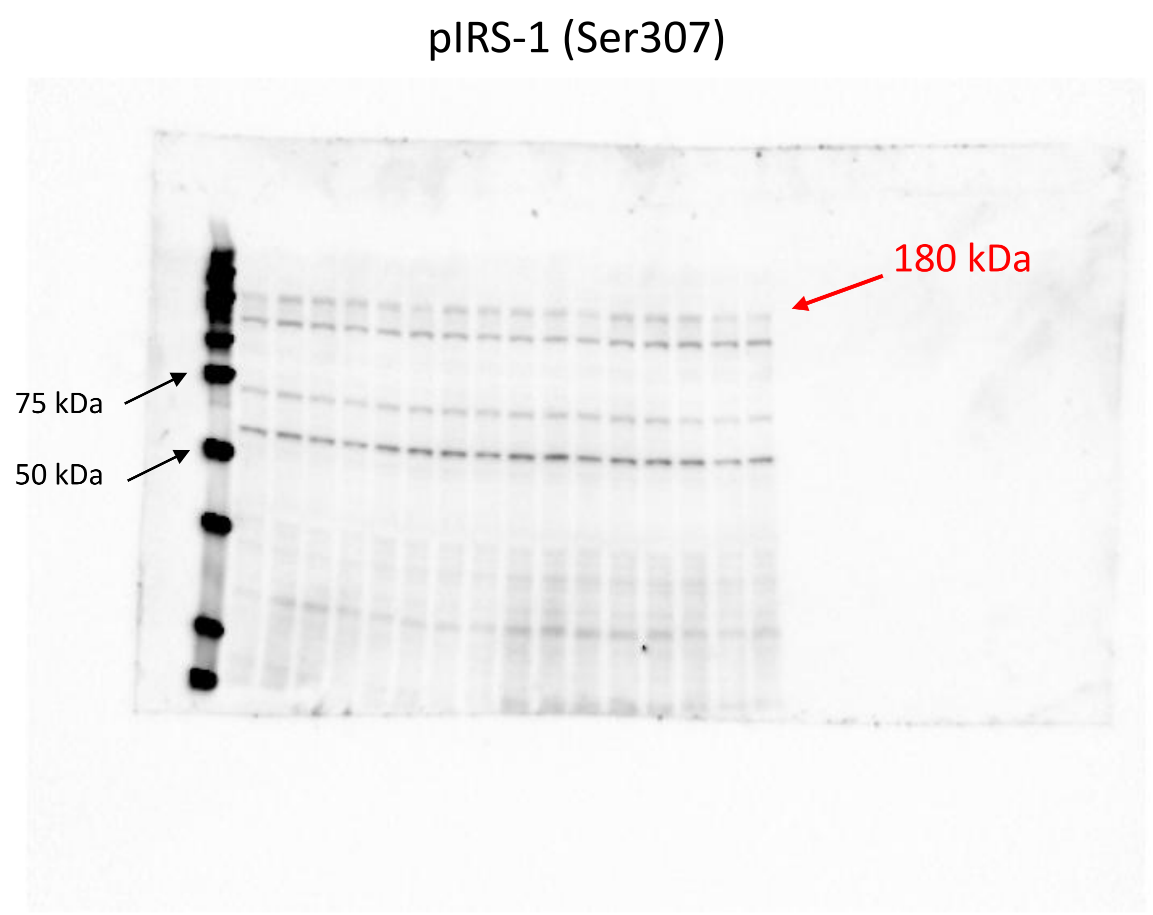

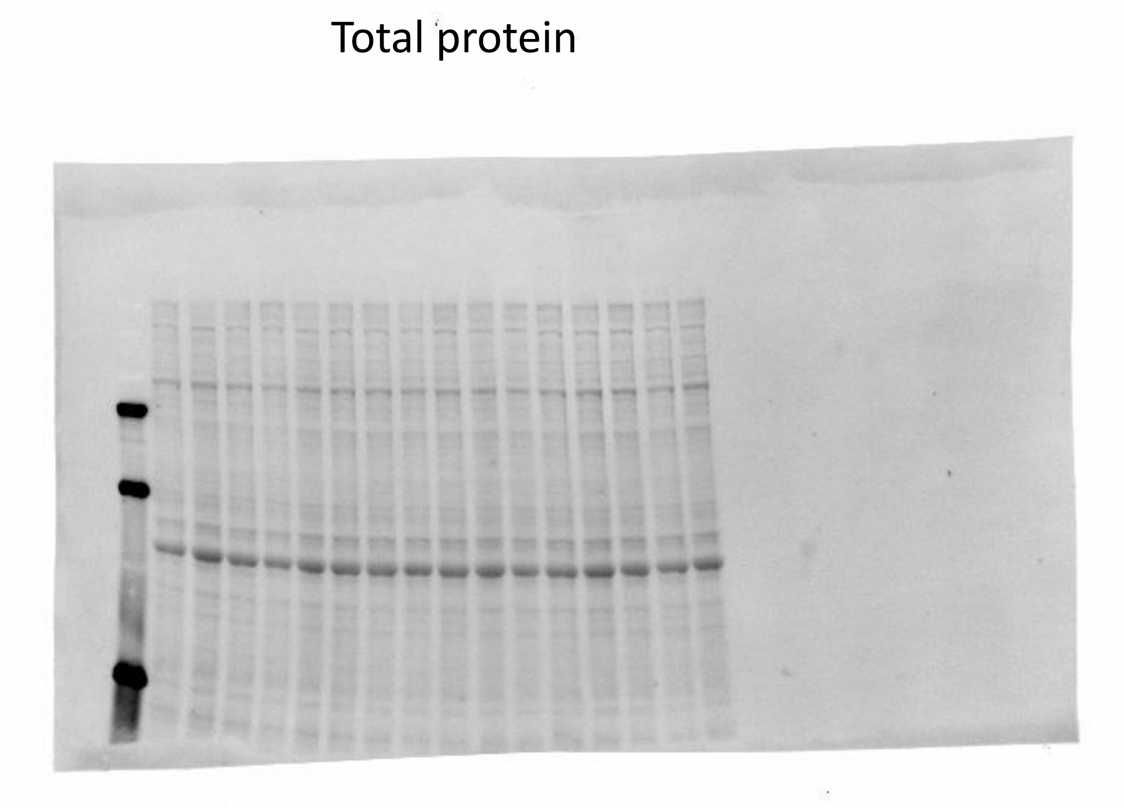

Loading order in all proteins: Control, 10min, 20min, 30min, 45min
