# Supplementary material for: Short-Term Incubation of H9c2 Cardiomyocytes with Cannabigerol Attenuates Diacylglycerol Accumulation in Lipid Overload Conditions
Source: Cells. 2025 Jun 30;14(13):998. doi: 10.3390/cells14130998 (PMC12249120; doi:10.3390/cells14130998)
Supplement: Supplementary file 1 [file cells-14-00998-s001.zip › cells-3659124-supplementary/preliminary studies-PA/Figure 2S.pdf]

Figure 2S. The content of free fatty acid - FFA (A), diacyloglycerol - DAG (B), triacyloglycerol - TAG (C), and phospholipid - PL (D), fractions in H9c2 cardiomyocytes after 18h incubation with palmitate (PA) in the concentration of 200 μM 300 μM. The data are expressed in nmol/mg of protein as mean values ± SD and are based on six independent determinations in each group. $^{a}p < 0.05$ indicates a significant difference between: the control group in comparison to the examined group.
