# Supplementary material for: Short-Term Incubation of H9c2 Cardiomyocytes with Cannabigerol Attenuates Diacylglycerol Accumulation in Lipid Overload Conditions
Source: Cells. 2025 Jun 30;14(13):998. doi: 10.3390/cells14130998 (PMC12249120; doi:10.3390/cells14130998)
Supplement: Supplementary file 1 [file cells-14-00998-s001.zip › cells-3659124-supplementary/Supplementary Materials-Western Blott.pdf]

Analysis was repeated and it was not used in the manuscript

CD36

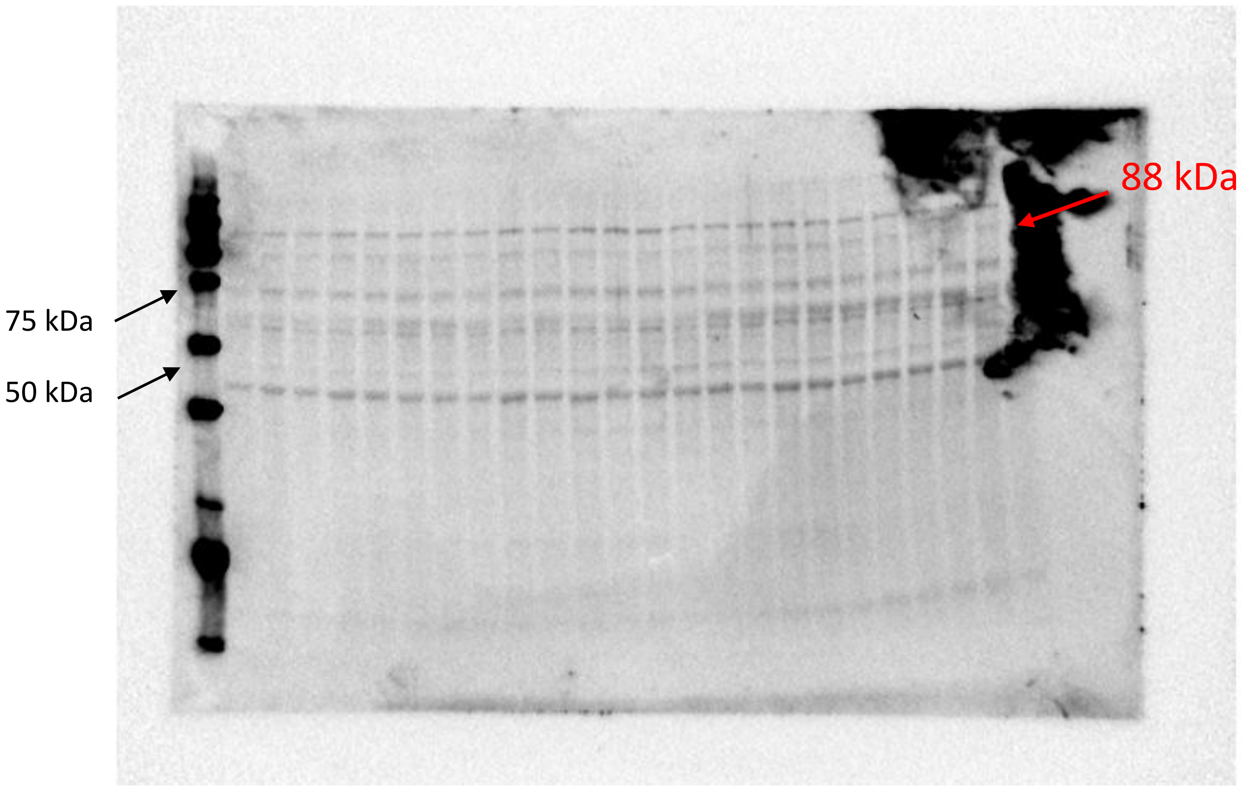

Total protein

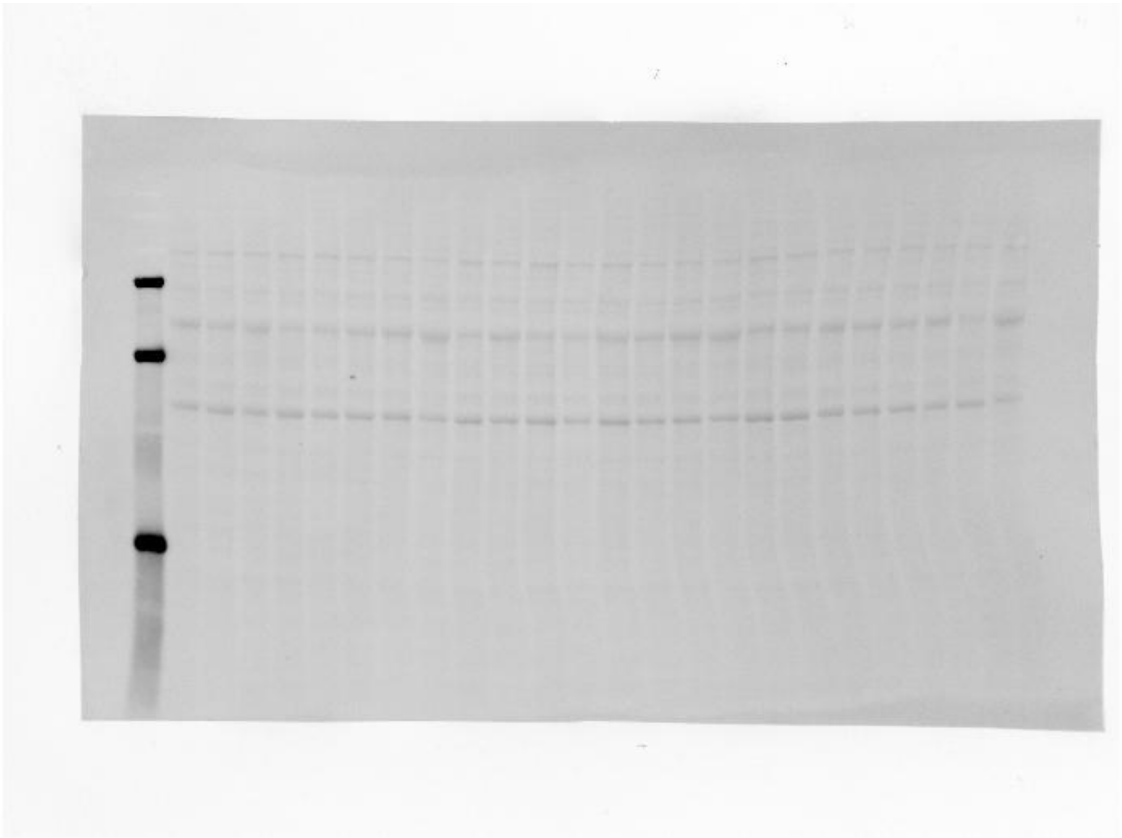

Loading order in all proteins: Control, CBG 2.5, CBG 5, CBG 10, PA, PA+CBG 2.5, PA+CBG 5, PA+CBG 10

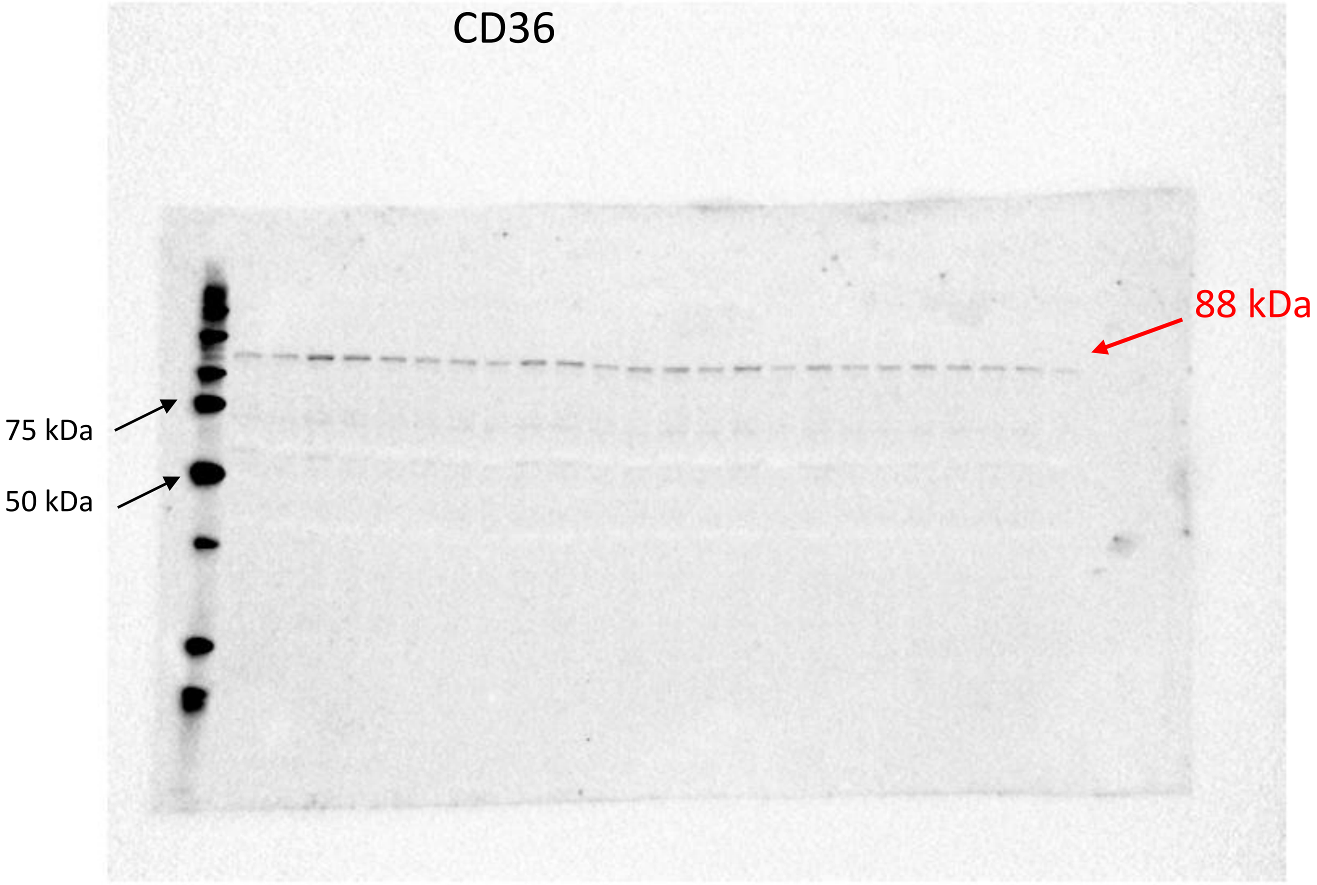

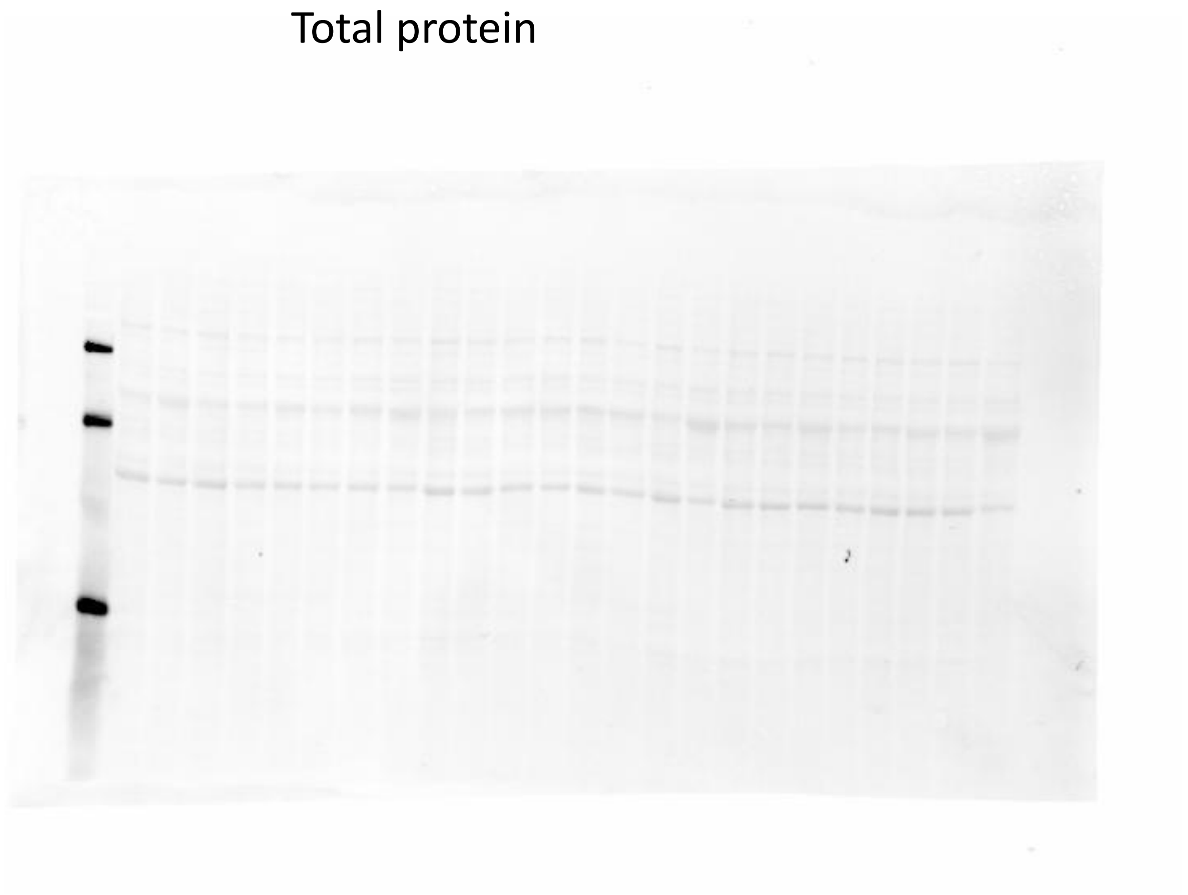

Loading order in all proteins: Control, CBG 2.5, CBG 5, CBG 10, PA, PA+CBG 2.5, PA+CBG 5, PA+CBG 10

Analysis was repeated and it was not used in the manuscript

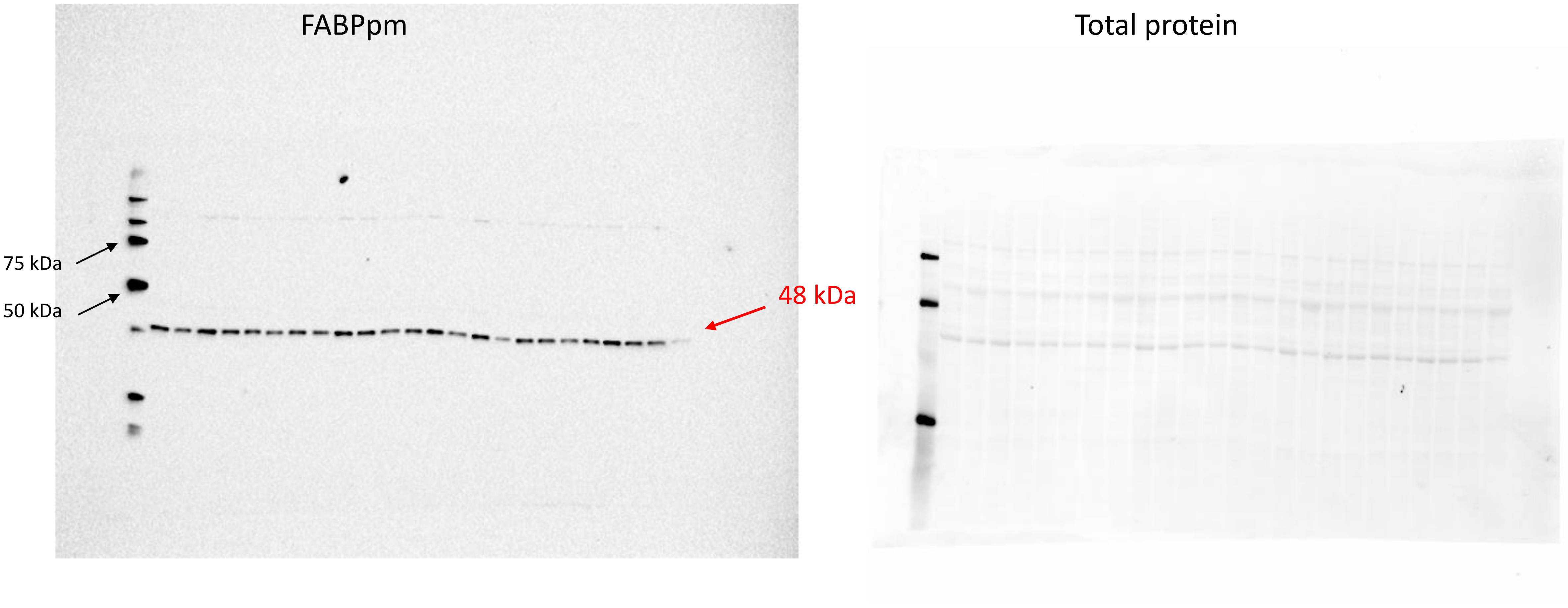

Loading order in all proteins: Control, CBG 2.5, CBG 5, CBG 10, PA, PA+CBG 2.5, PA+CBG 5, PA+CBG 10

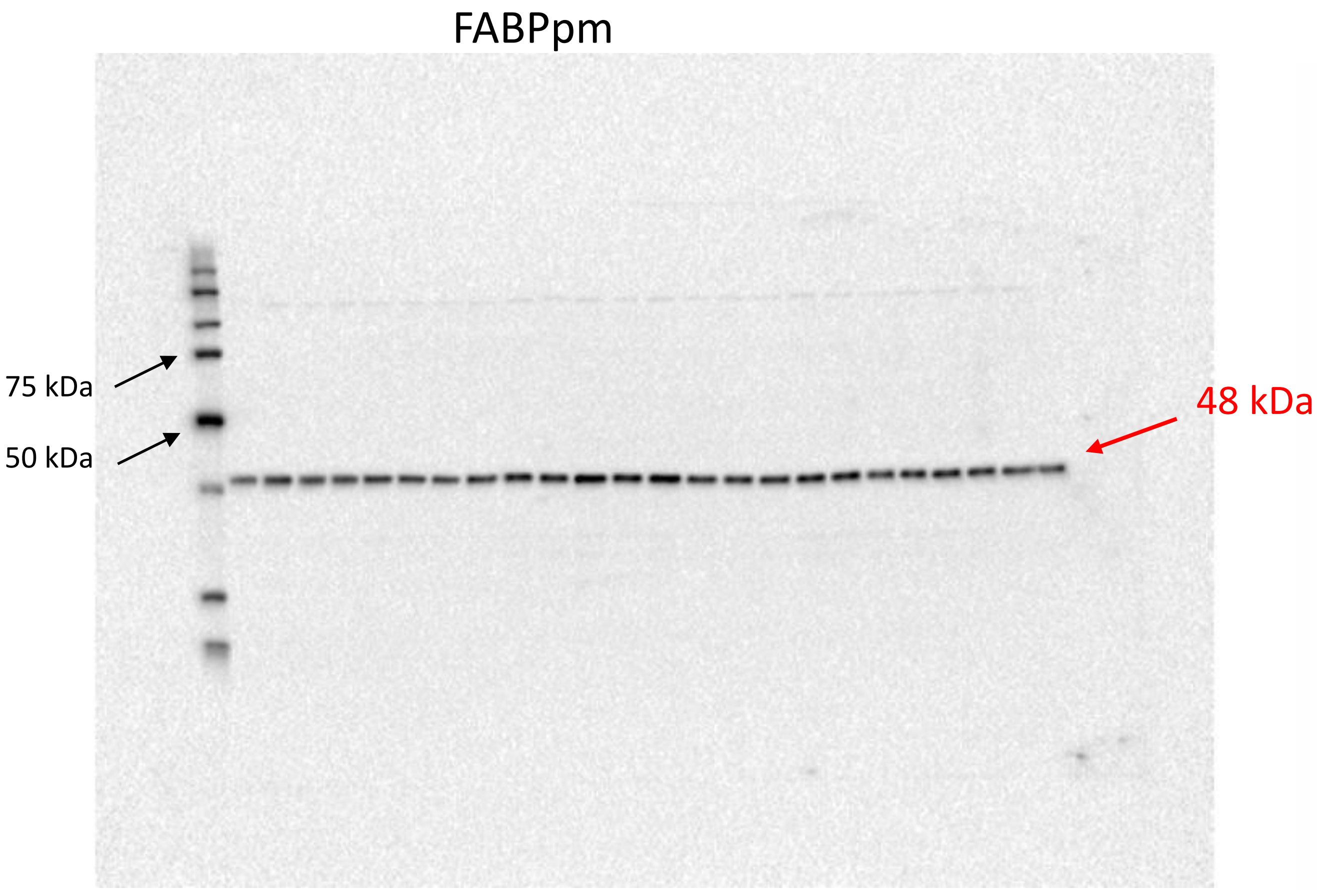

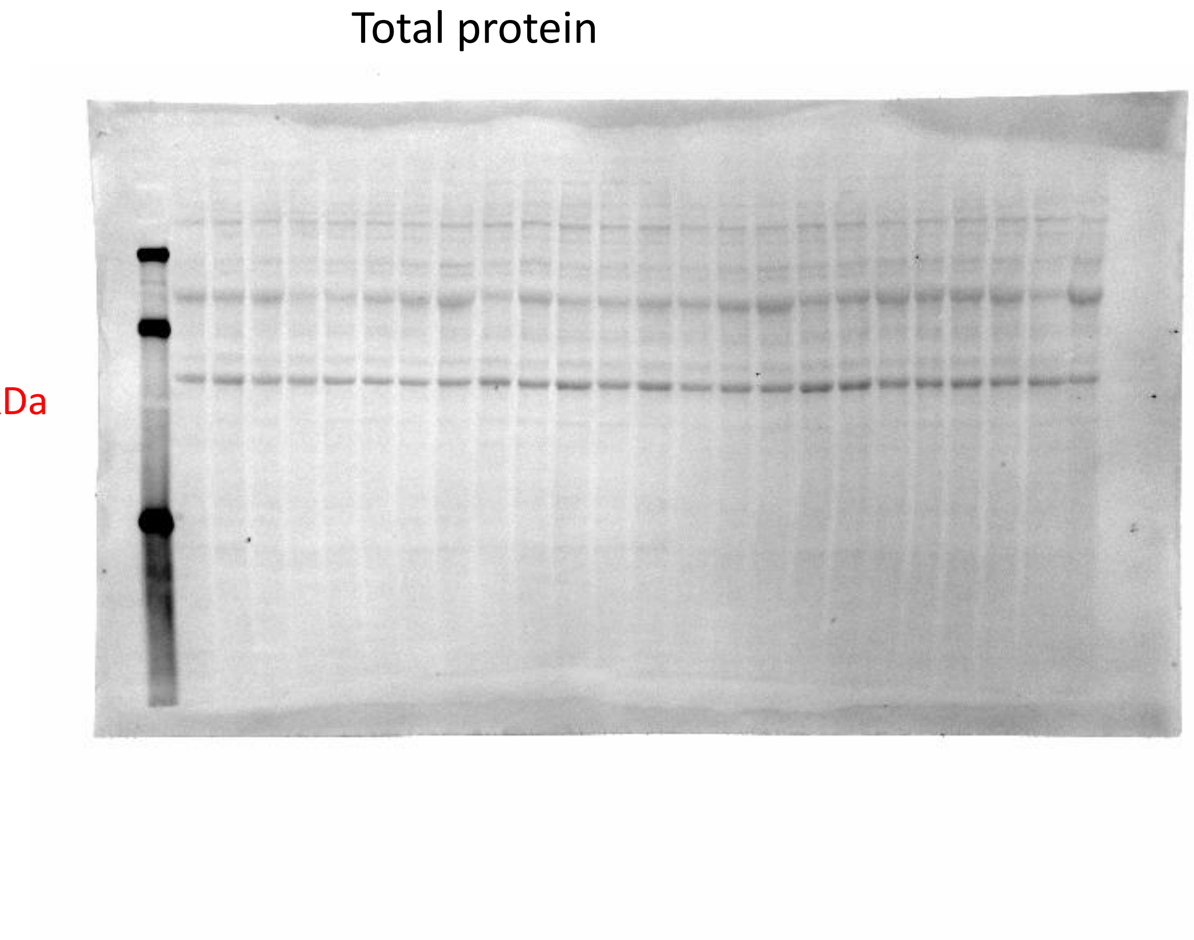

Loading order in all proteins: Control, CBG 2.5, CBG 5, CBG 10, PA, PA+CBG 2.5, PA+CBG 5, PA+CBG 10

FATP-1

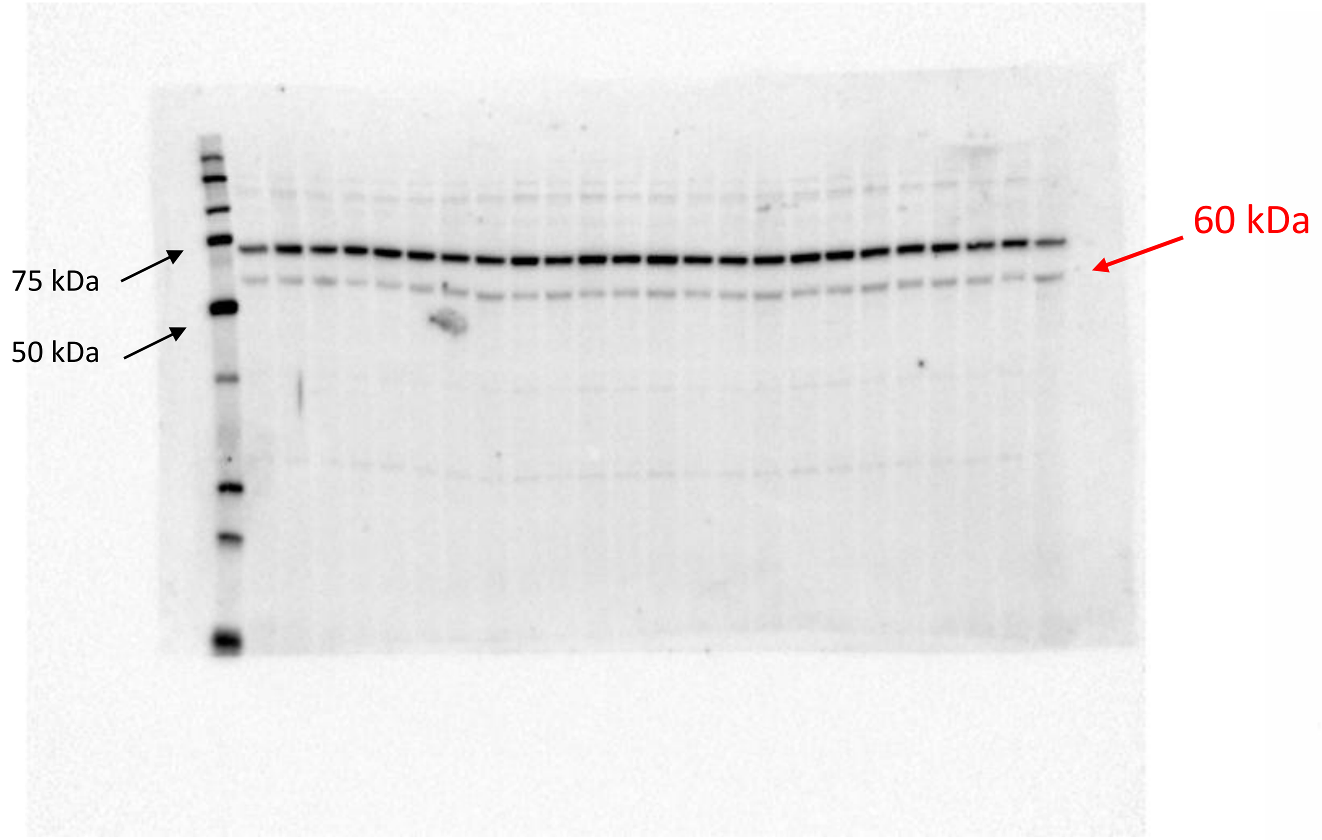

Total protein

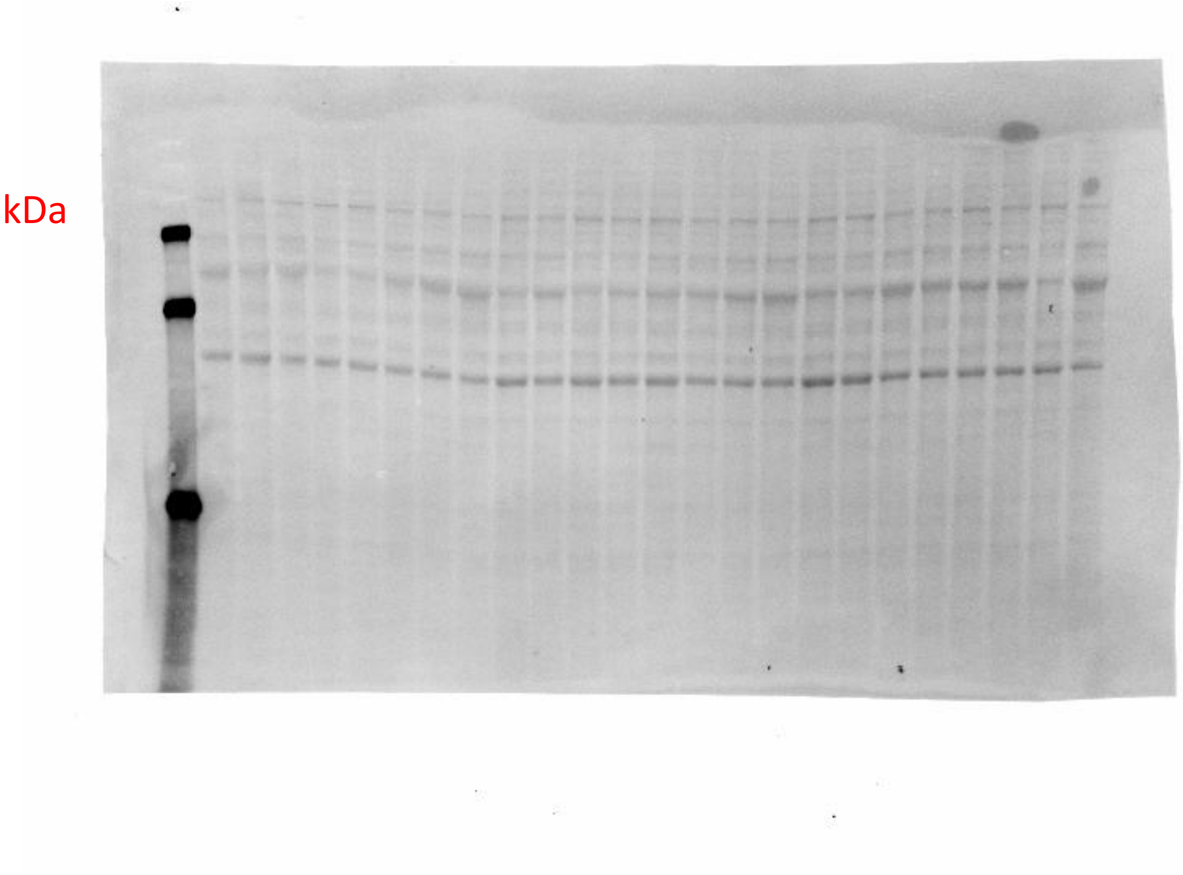

Loading order in all proteins: Control, CBG 2.5, CBG 5, CBG 10, PA, PA+CBG 2.5, PA+CBG 5, PA+CBG 10

Analysis was repeated and it was not used in the manuscript

FATP-4

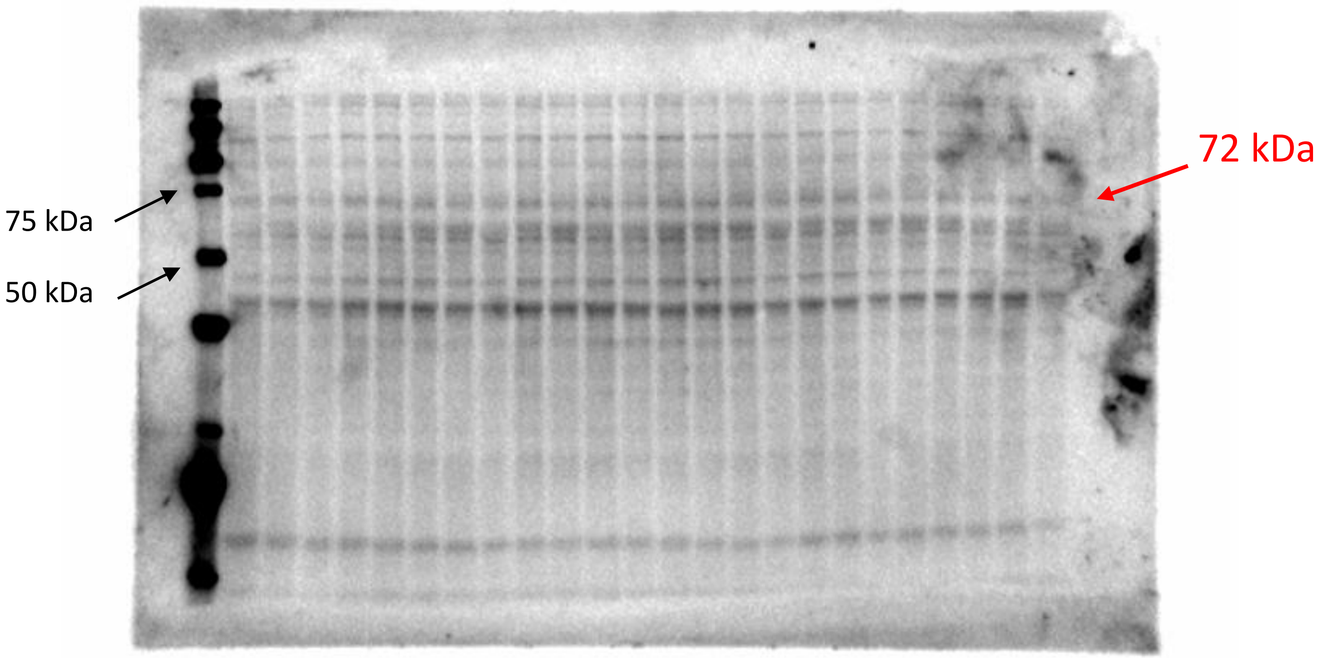

Total protein

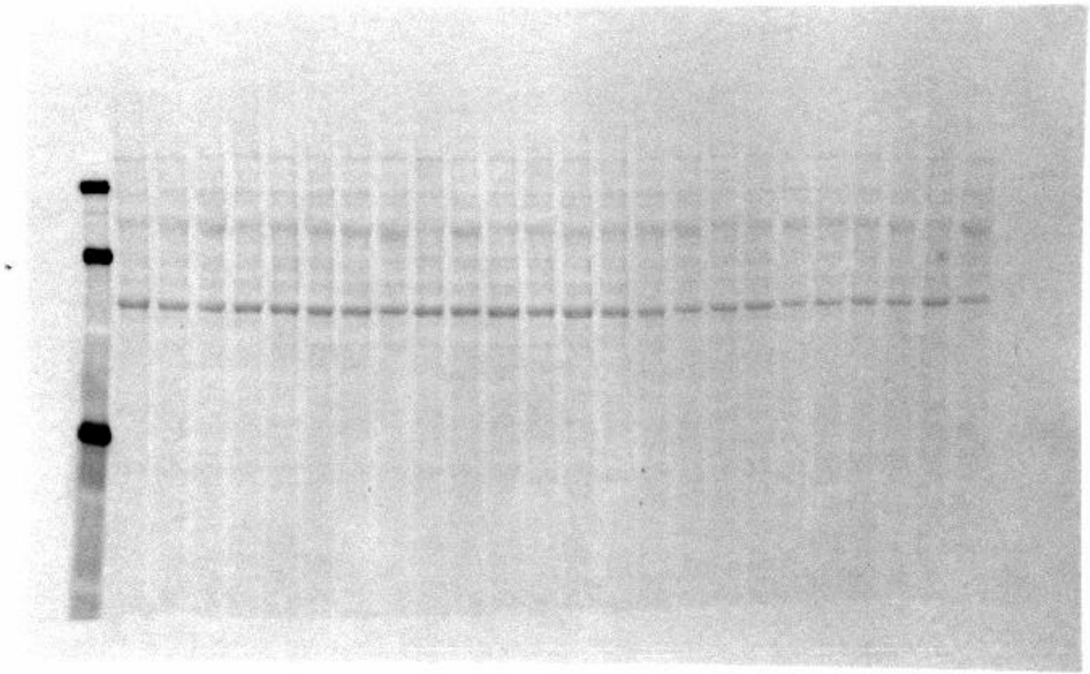

Loading order in all proteins: Control, CBG 2.5, CBG 5, CBG 10, PA, PA+CBG 2.5, PA+CBG 5, PA+CBG 10

FATP-4

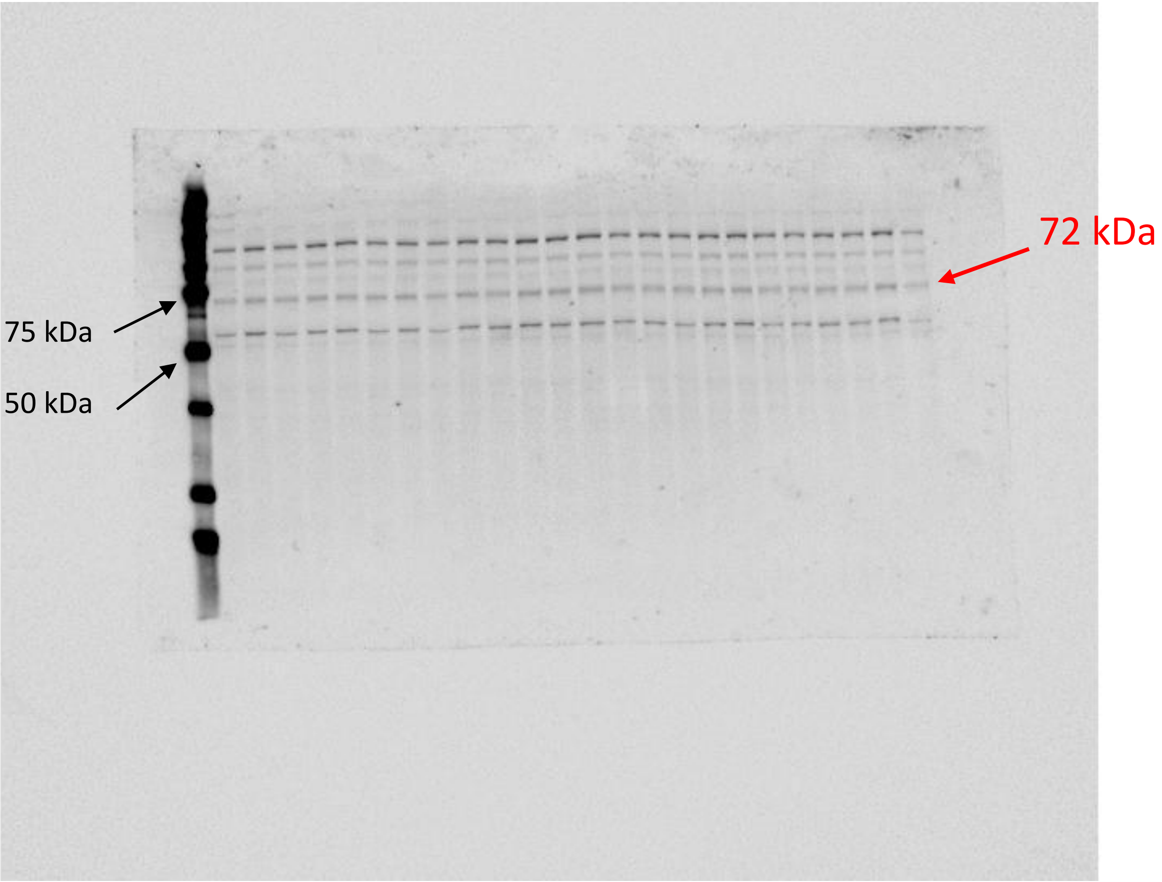

Total protein

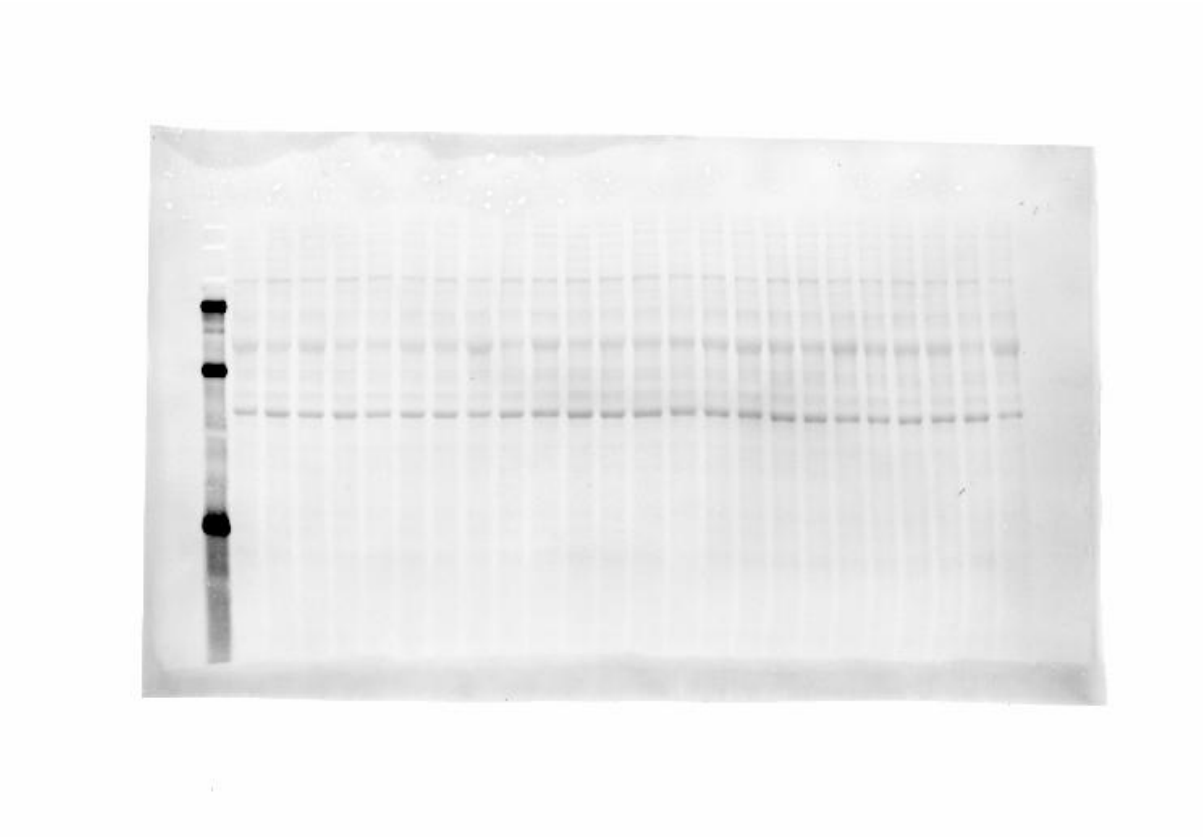

Loading order in all proteins: Control, CBG 2.5, CBG 5, CBG 10, PA, PA+CBG 2.5, PA+CBG 5, PA+CBG 10

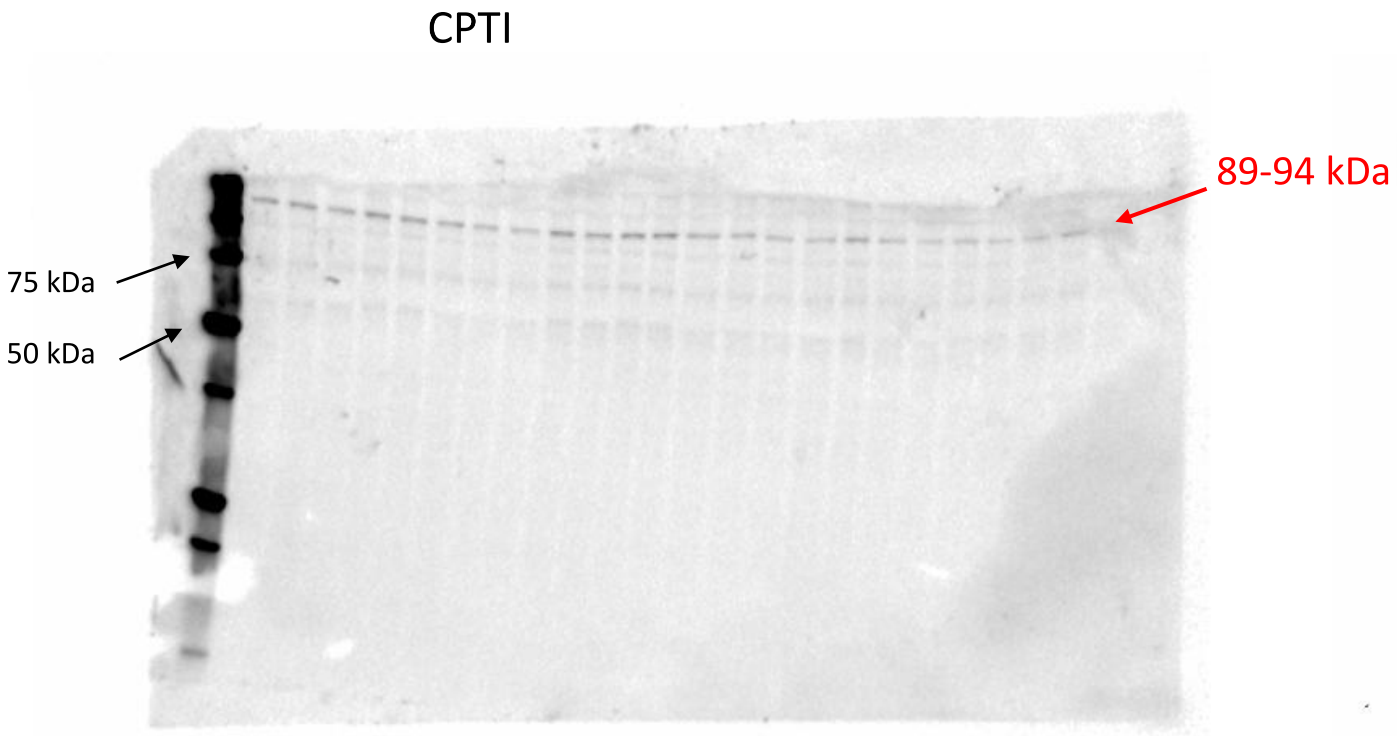

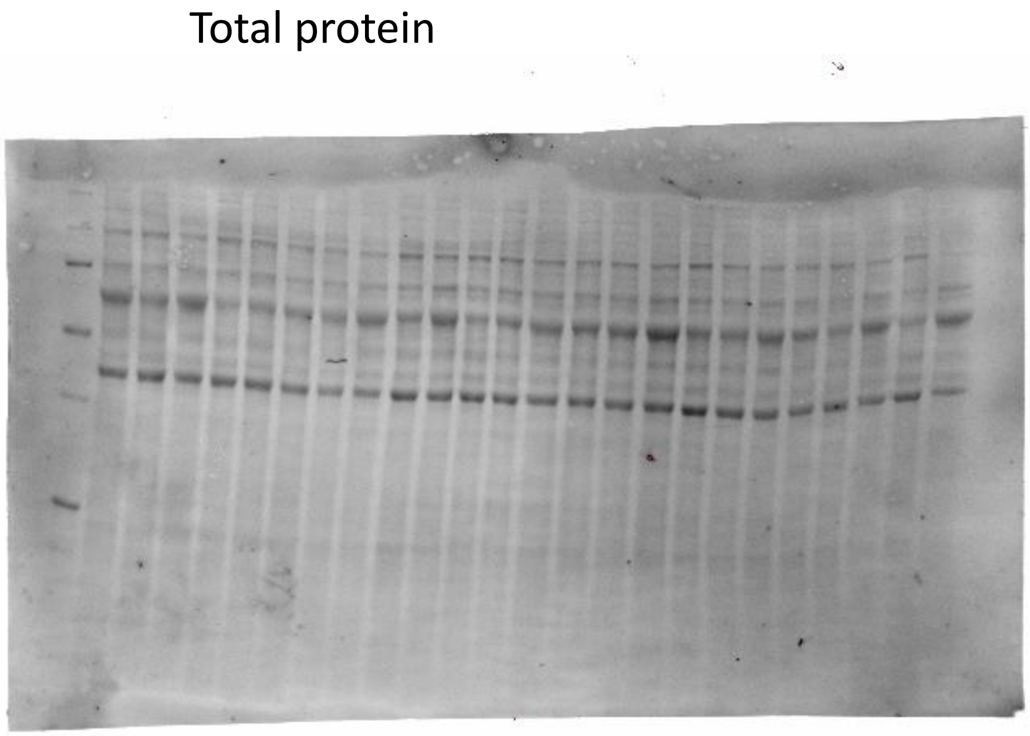

Loading order in all proteins: Control, CBG 2.5, CBG 5, CBG 10, PA, PA+CBG 2.5, PA+CBG 5, PA+CBG 10

CS

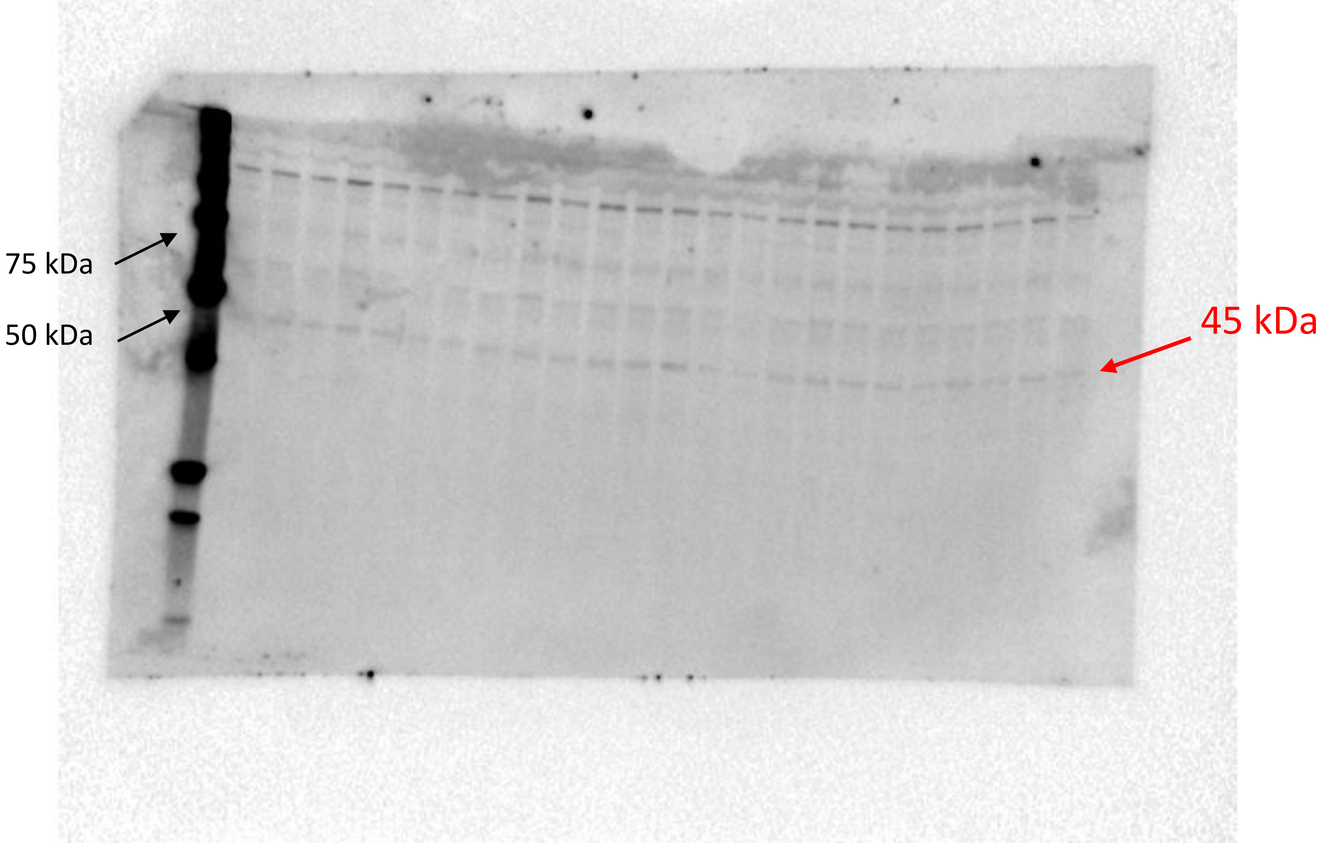

Total protein

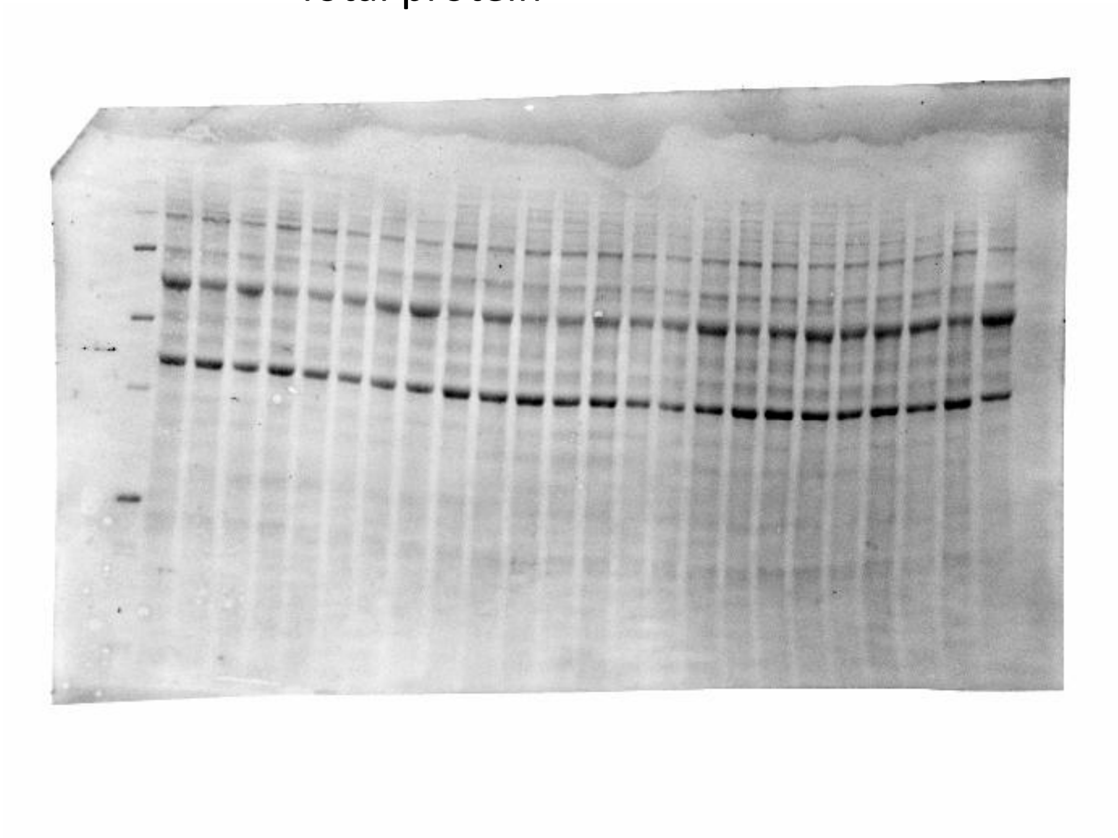

Loading order in all proteins: Control, CBG 2.5, CBG 5, CBG 10, PA, PA+CBG 2.5, PA+CBG 5, PA+CBG 10

Analysis was repeated and it was not used in the manuscript

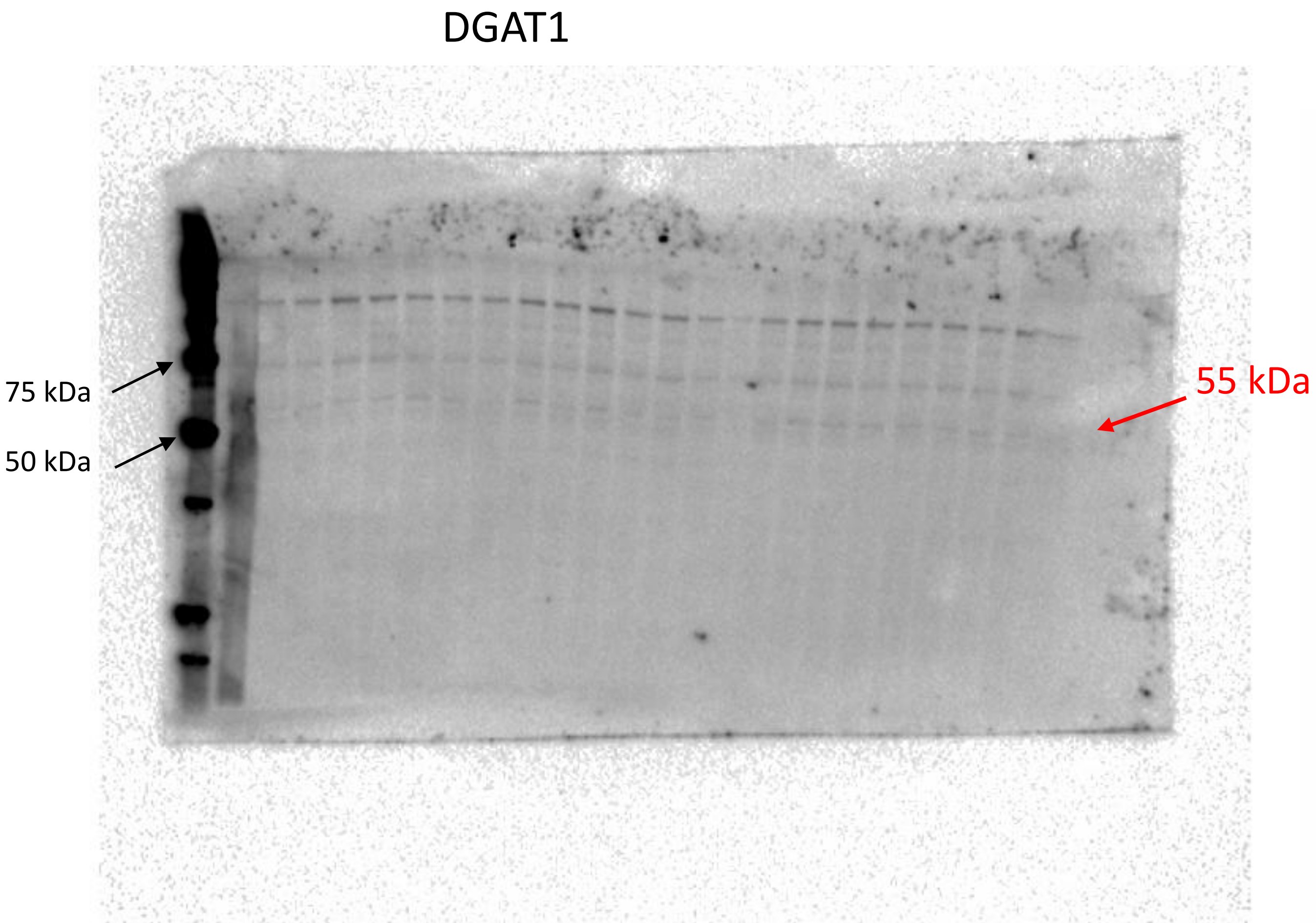

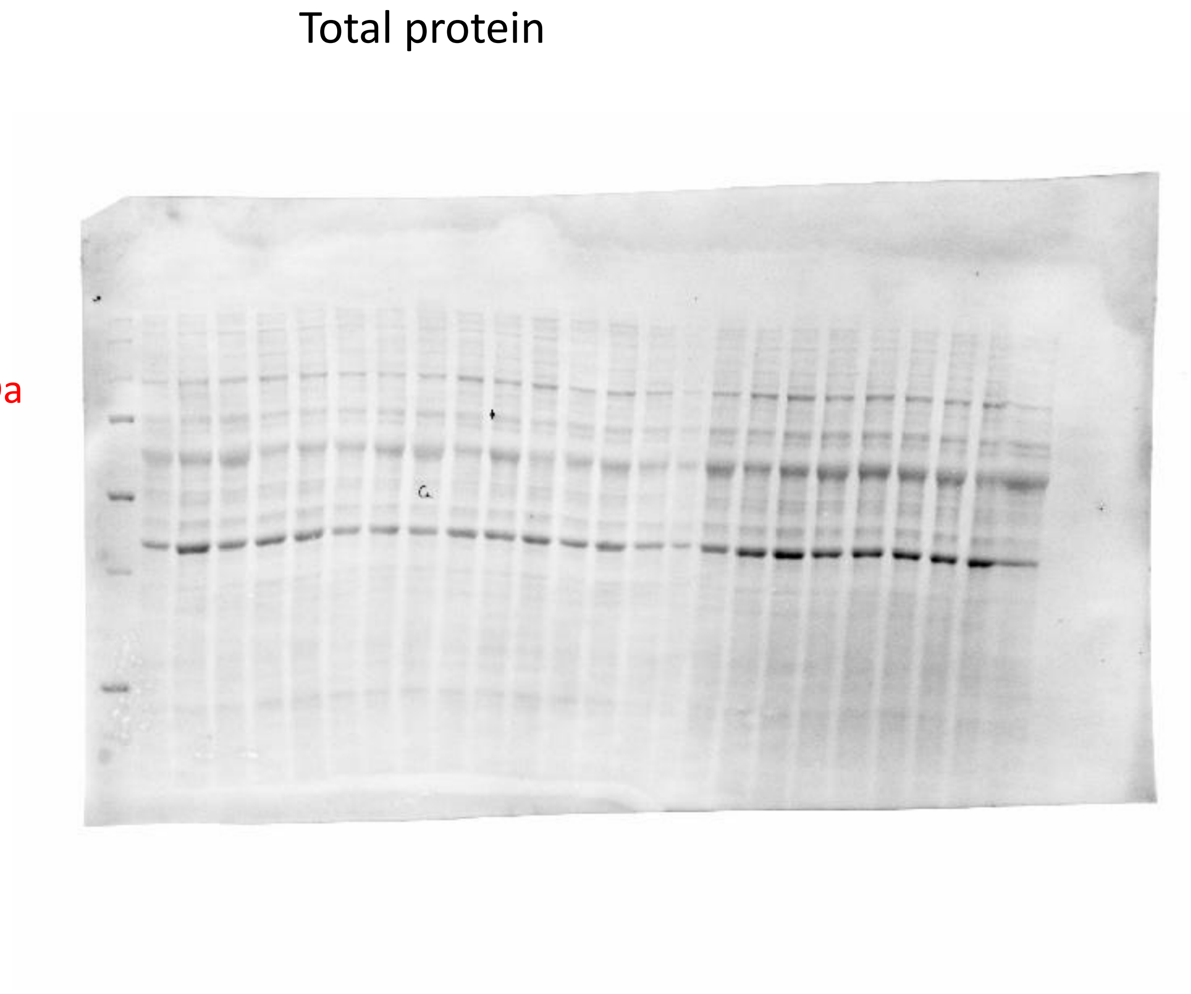

Loading order in all proteins: Control, CBG 2.5, CBG 5, CBG 10, PA, PA+CBG 2.5, PA+CBG 5, PA+CBG 10

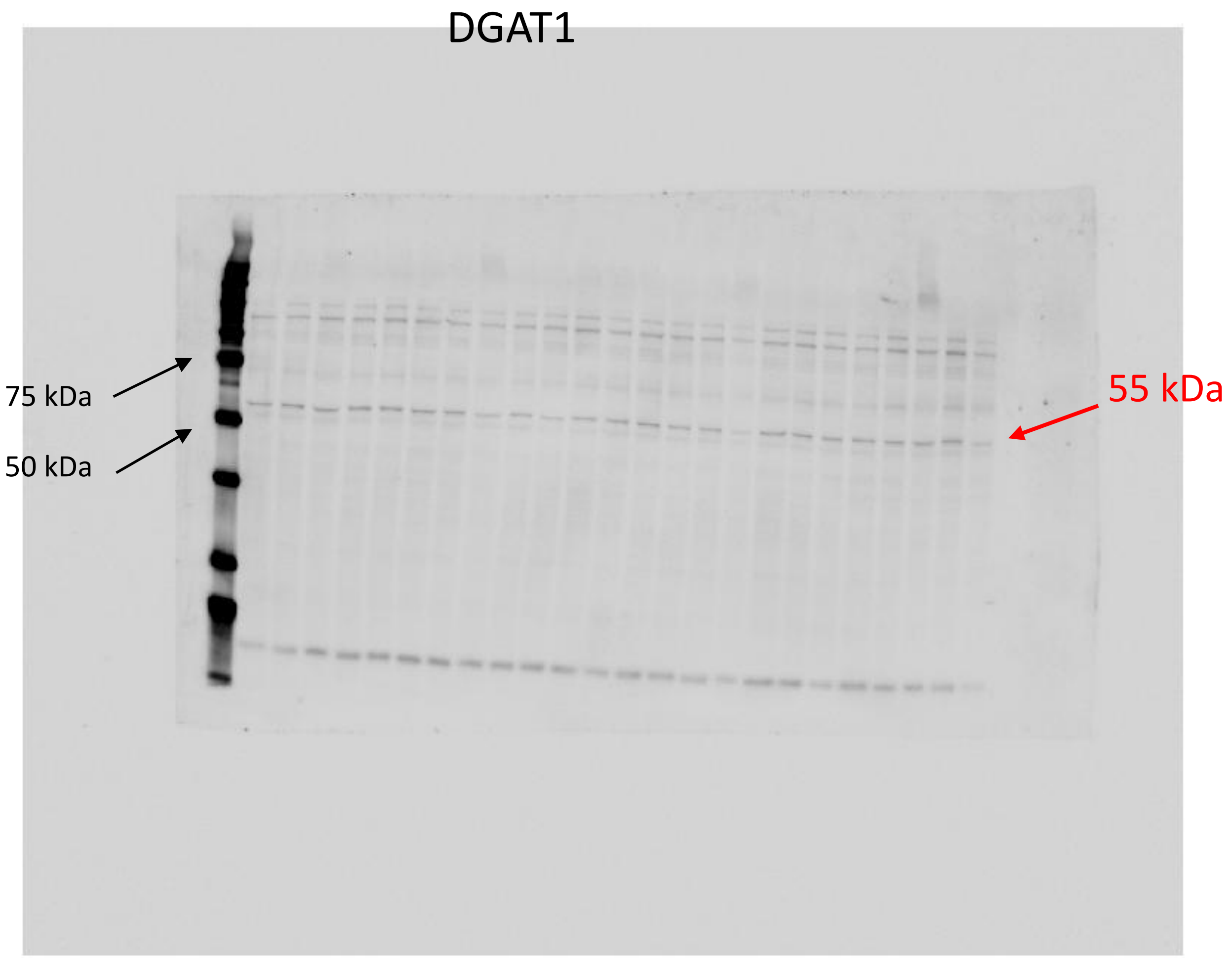

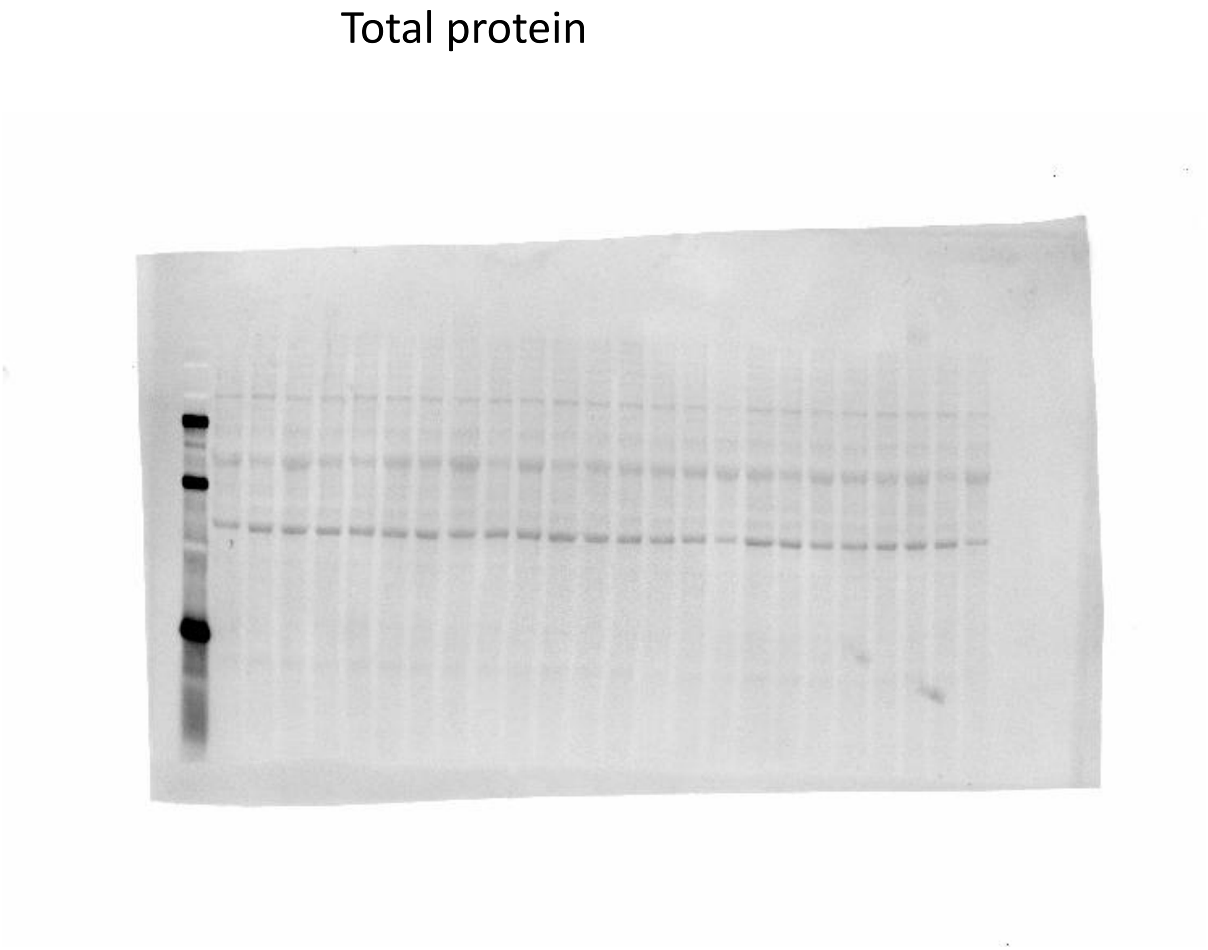

Loading order in all proteins: Control, CBG 2.5, CBG 5, CBG 10, PA, PA+CBG 2.5, PA+CBG 5, PA+CBG 10

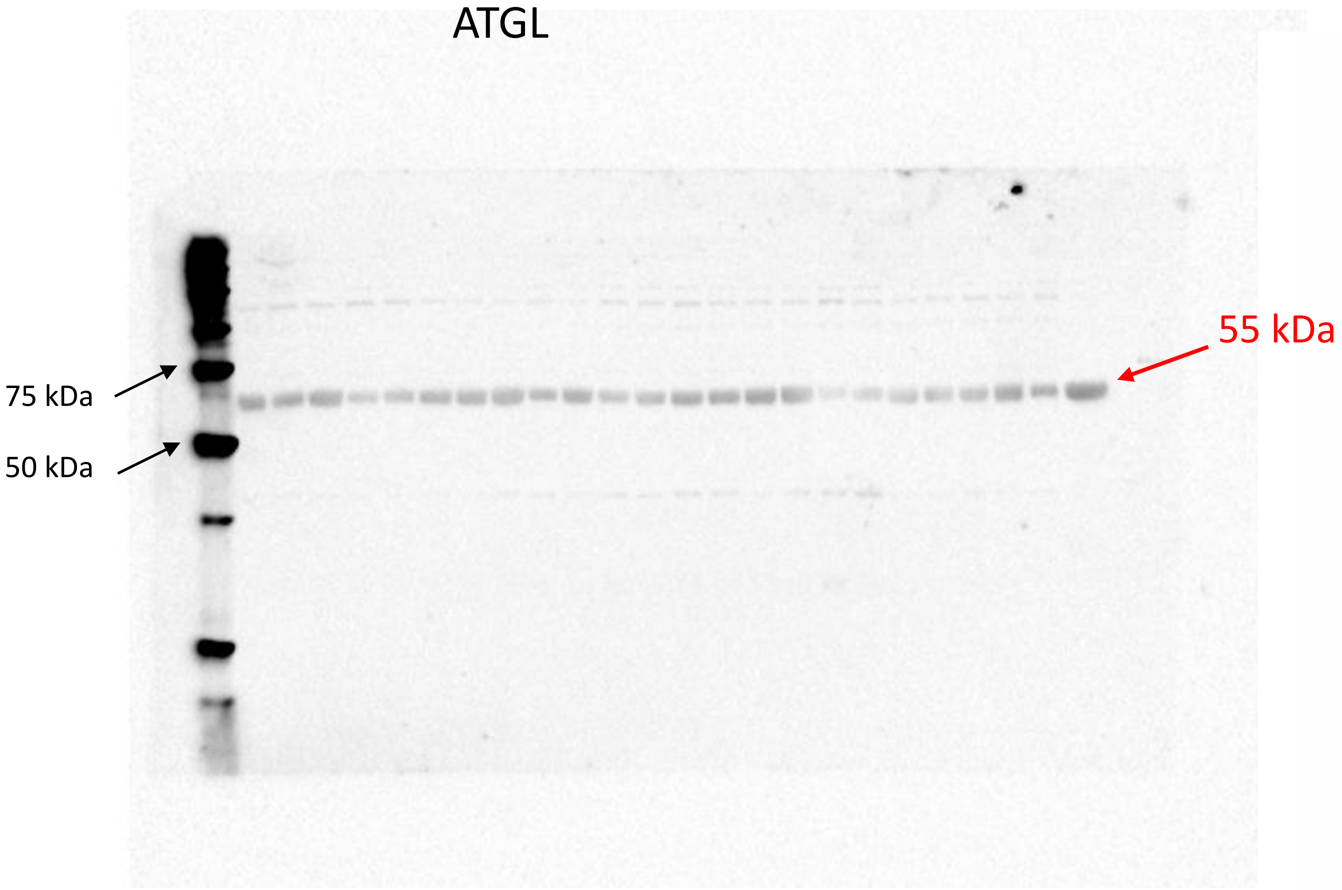

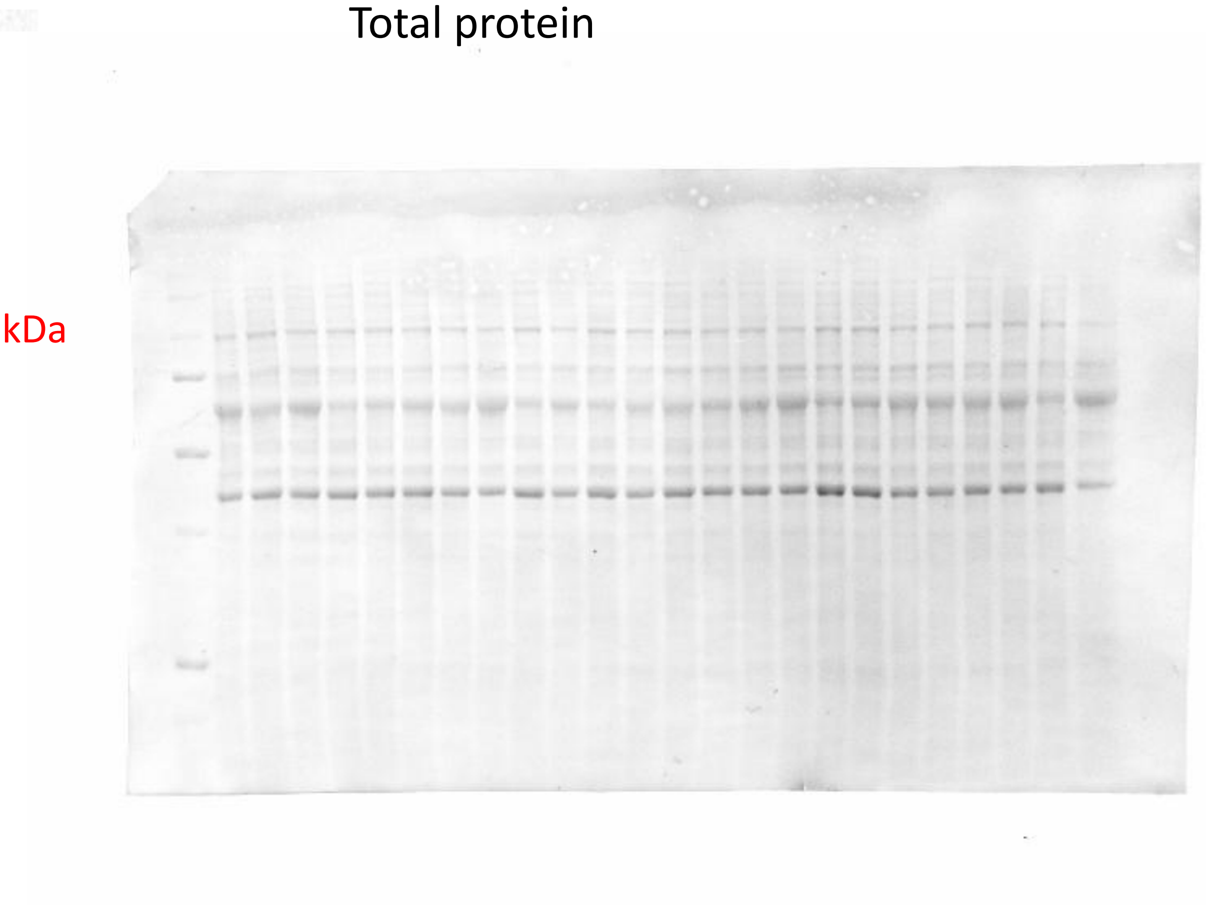

Loading order in all proteins: Control, CBG 2.5, CBG 5, CBG 10, PA, PA+CBG 2.5, PA+CBG 5, PA+CBG 10

Analysis was repeated and it was not used in the manuscript

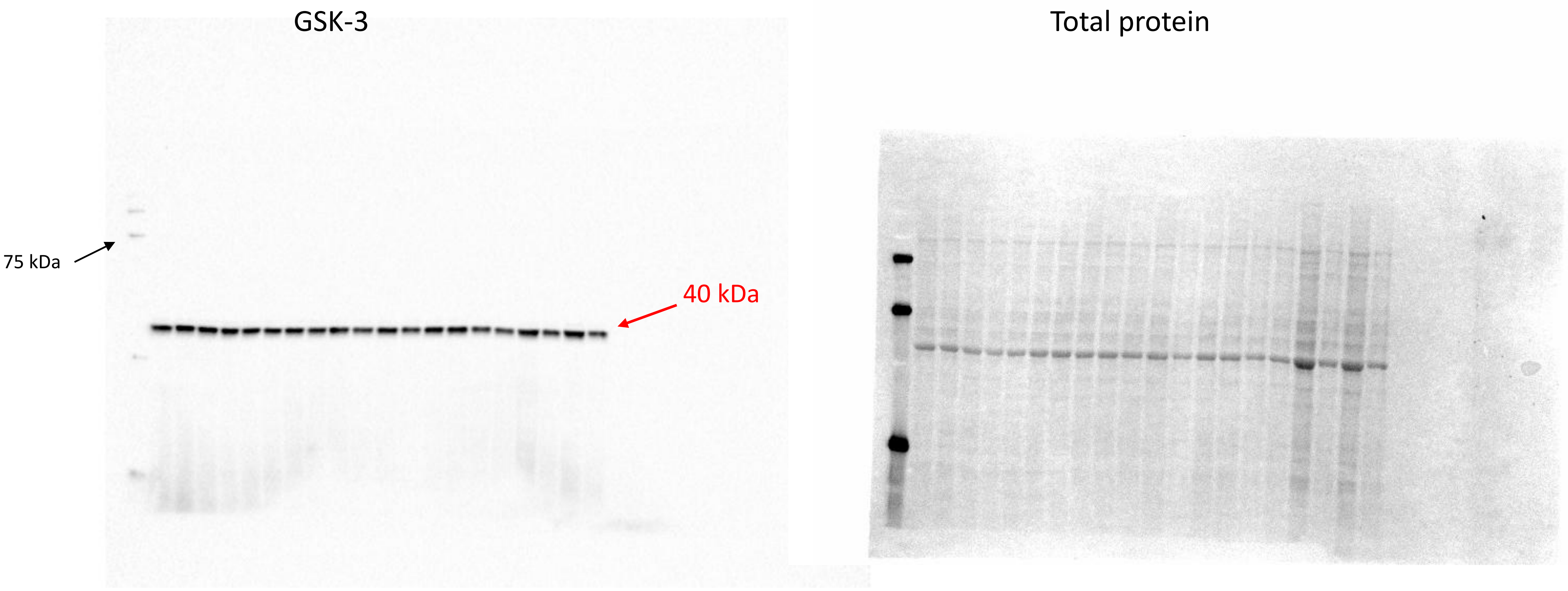

Loading order in all proteins: Control, INS, CBG+INS, PA+INS, PA+CBG+INS

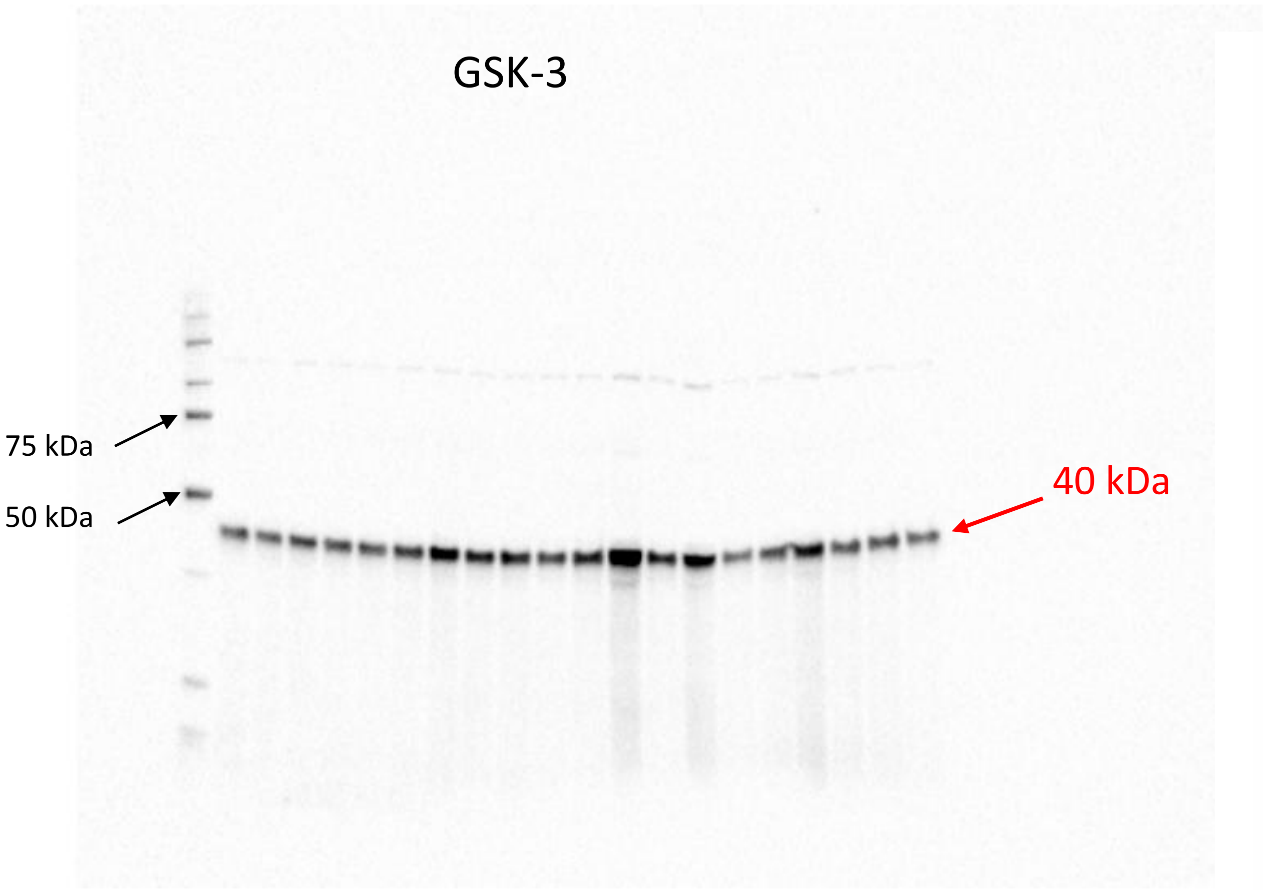

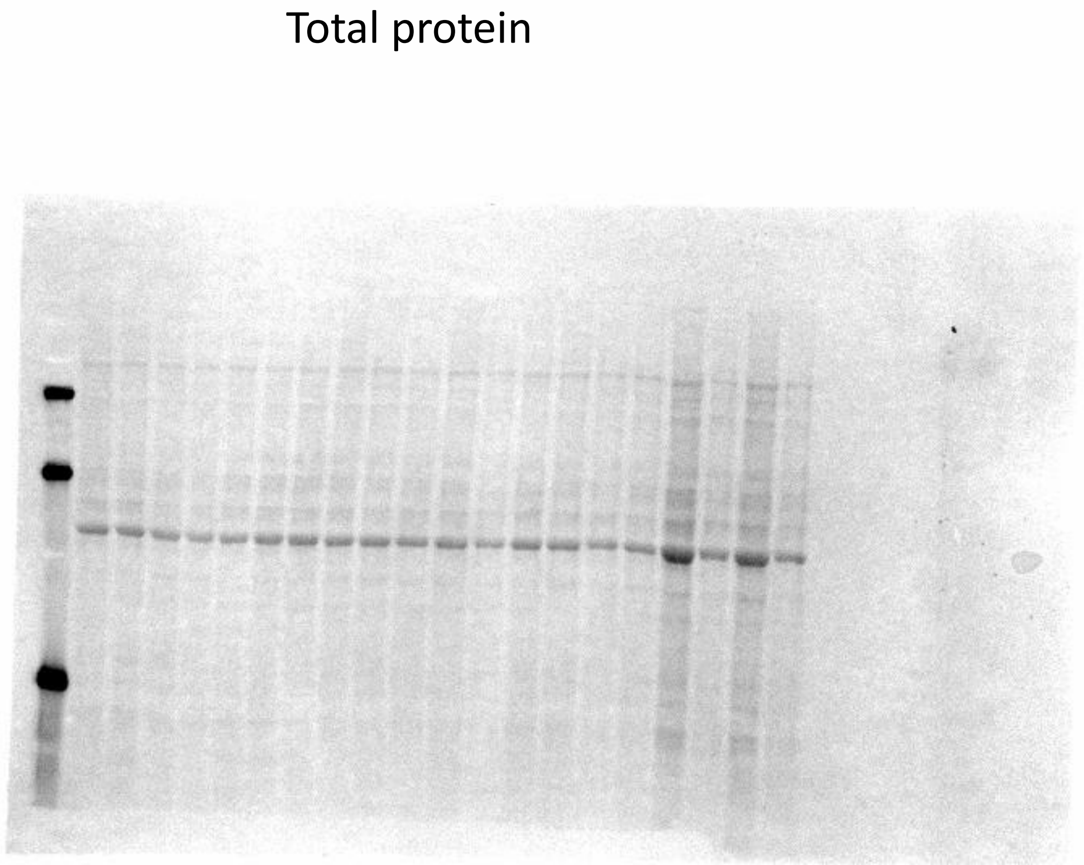

Loading order in all proteins: Control, INS, CBG+INS, PA+INS, PA+CBG+INS

Analysis was repeated and it was not used in the manuscript

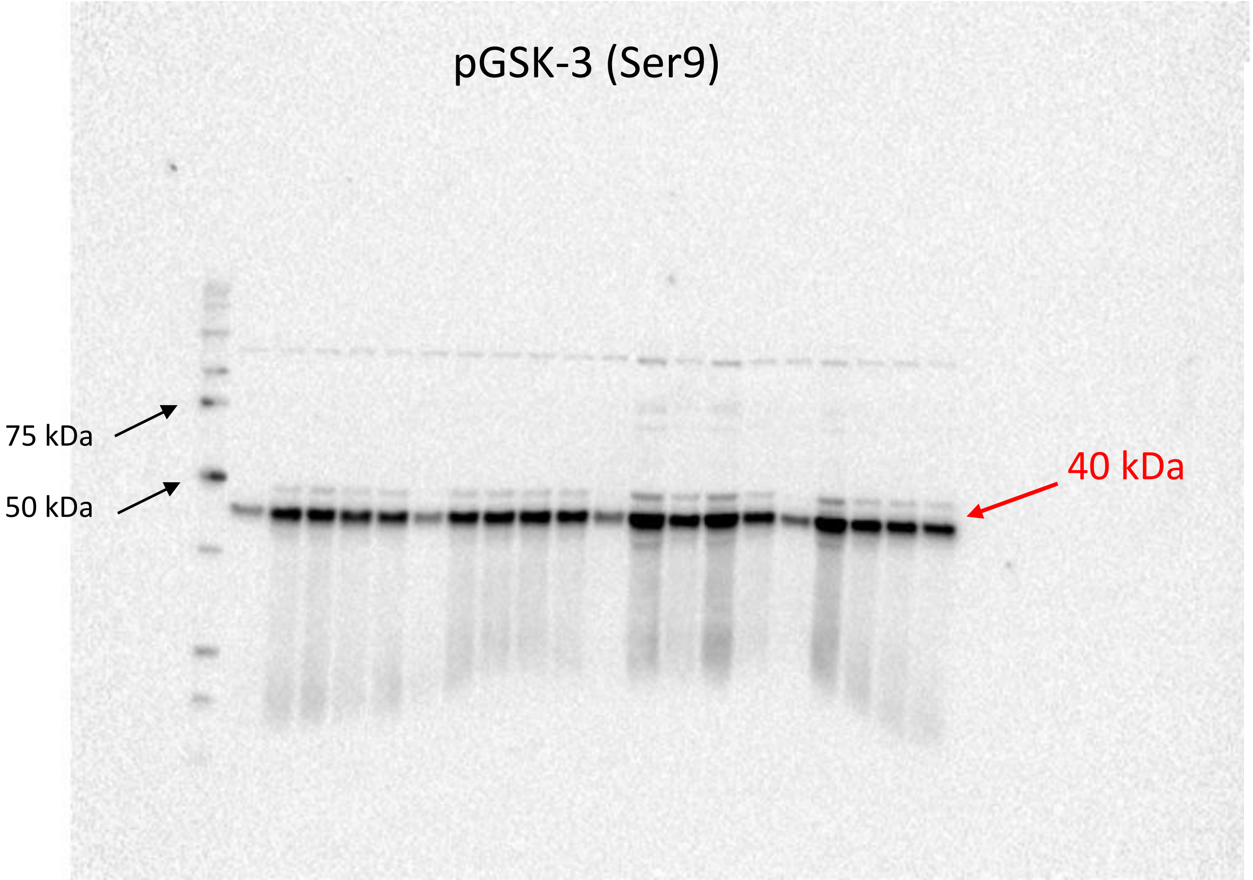

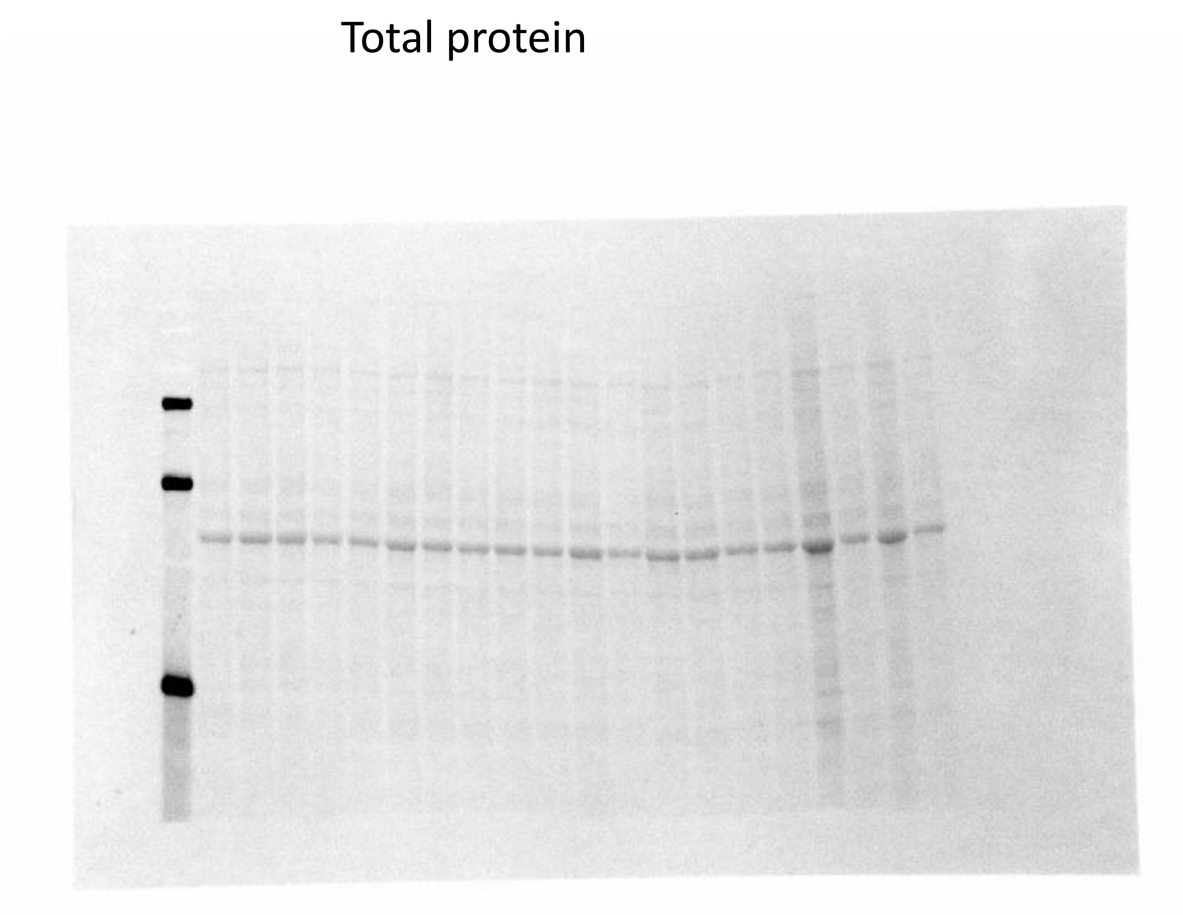

Loading order in all proteins: Control, INS, CBG+INS, PA+INS, PA+CBG+INS

pGSK-3 (Ser9)

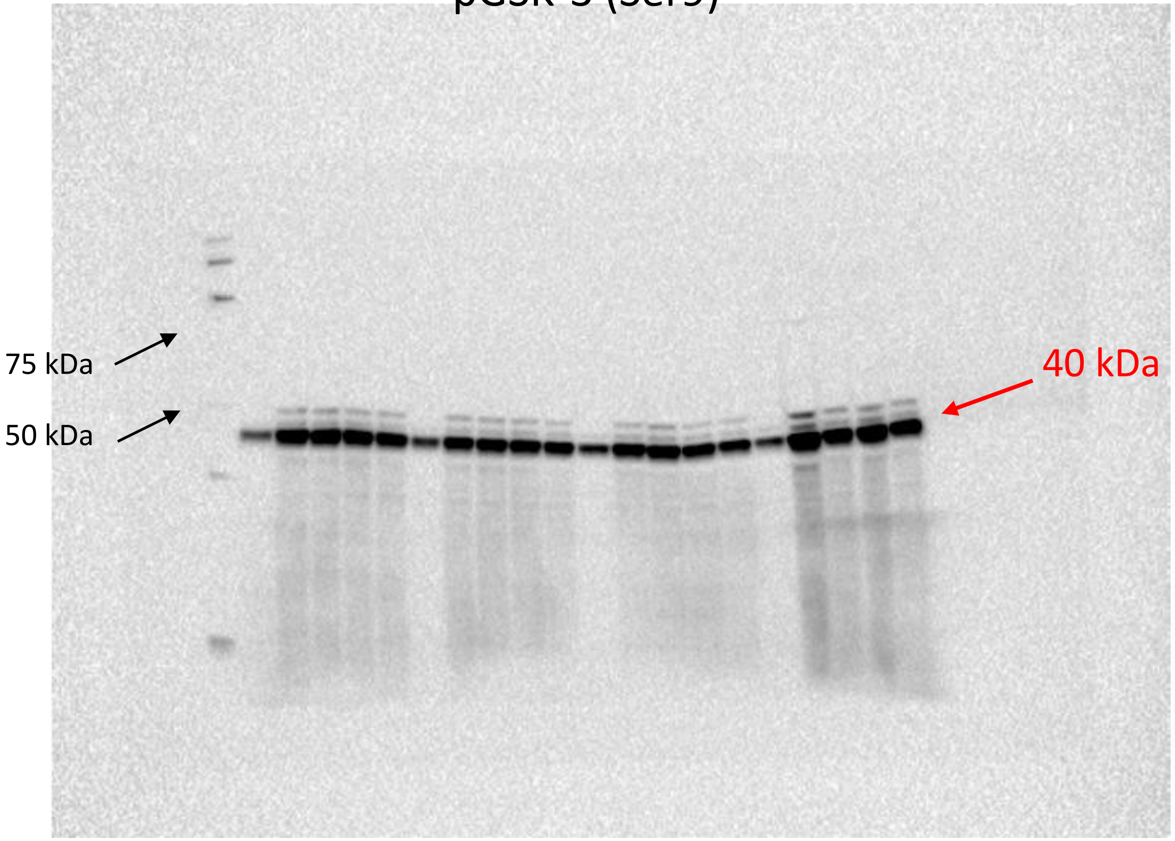

Total protein

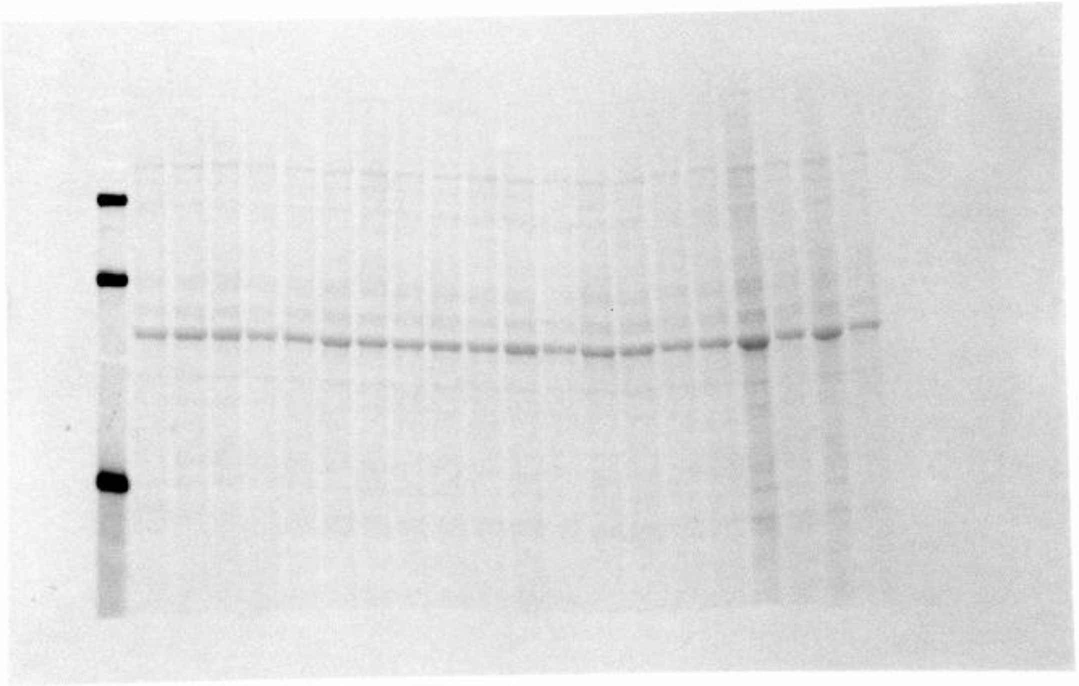

Loading order in all proteins: Control, INS, CBG+INS, PA+INS, PA+CBG+INS

Analysis was repeated and it was not used in the manuscript

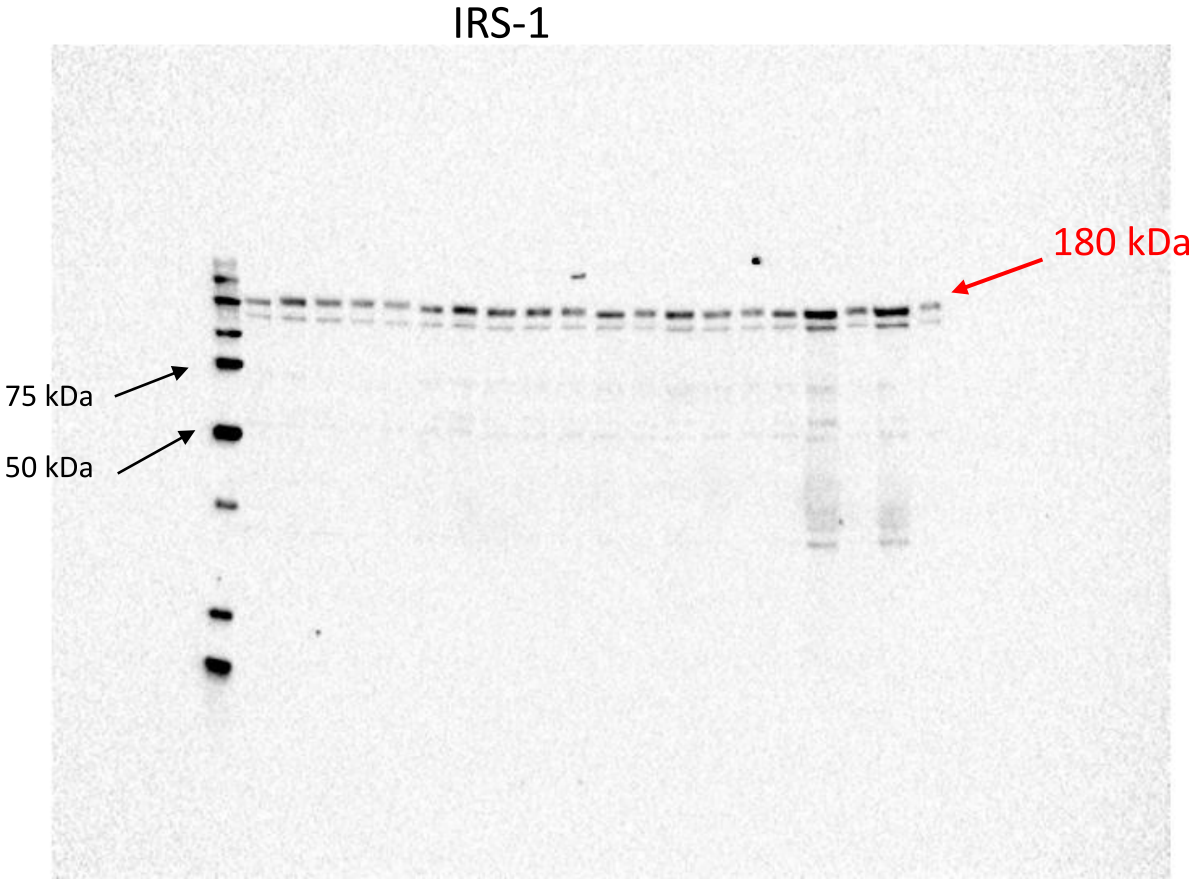

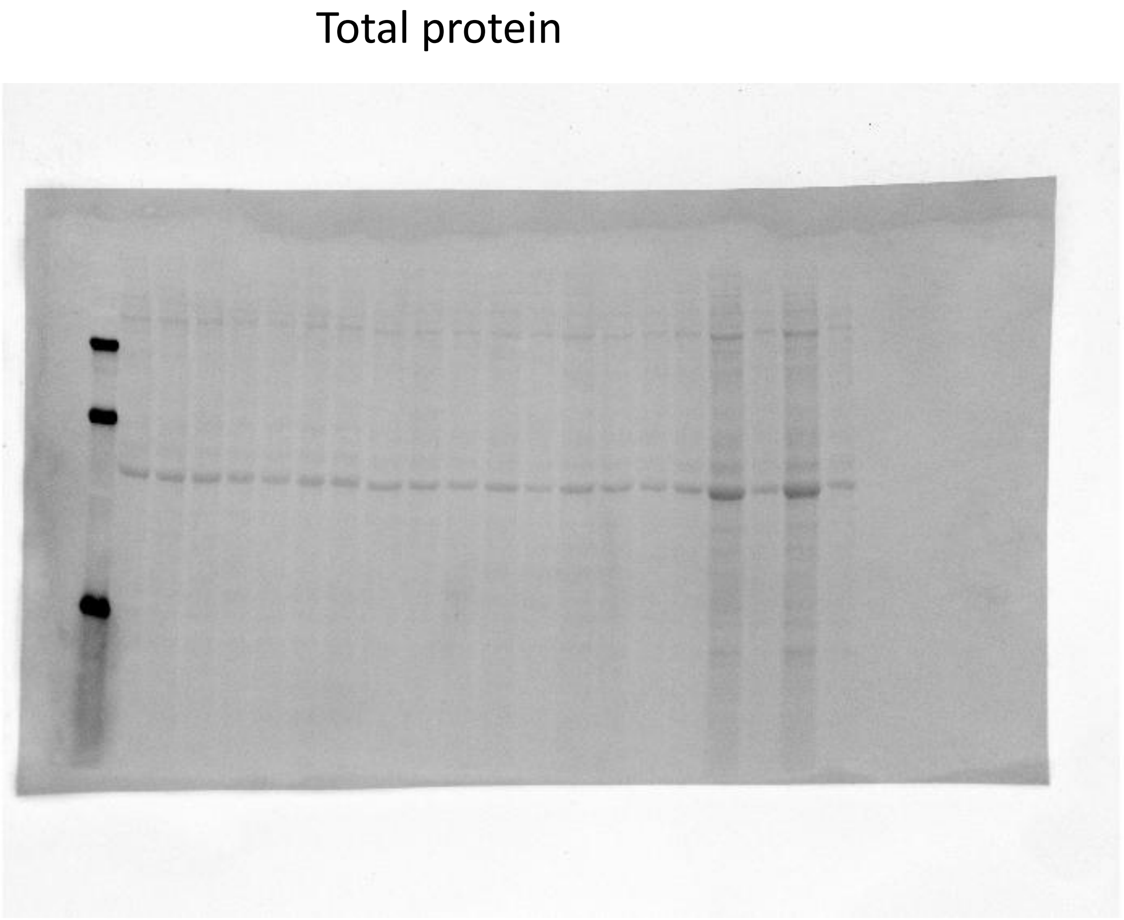

Loading order in all proteins: Control, INS, CBG+INS, PA+INS, PA+CBG+INS

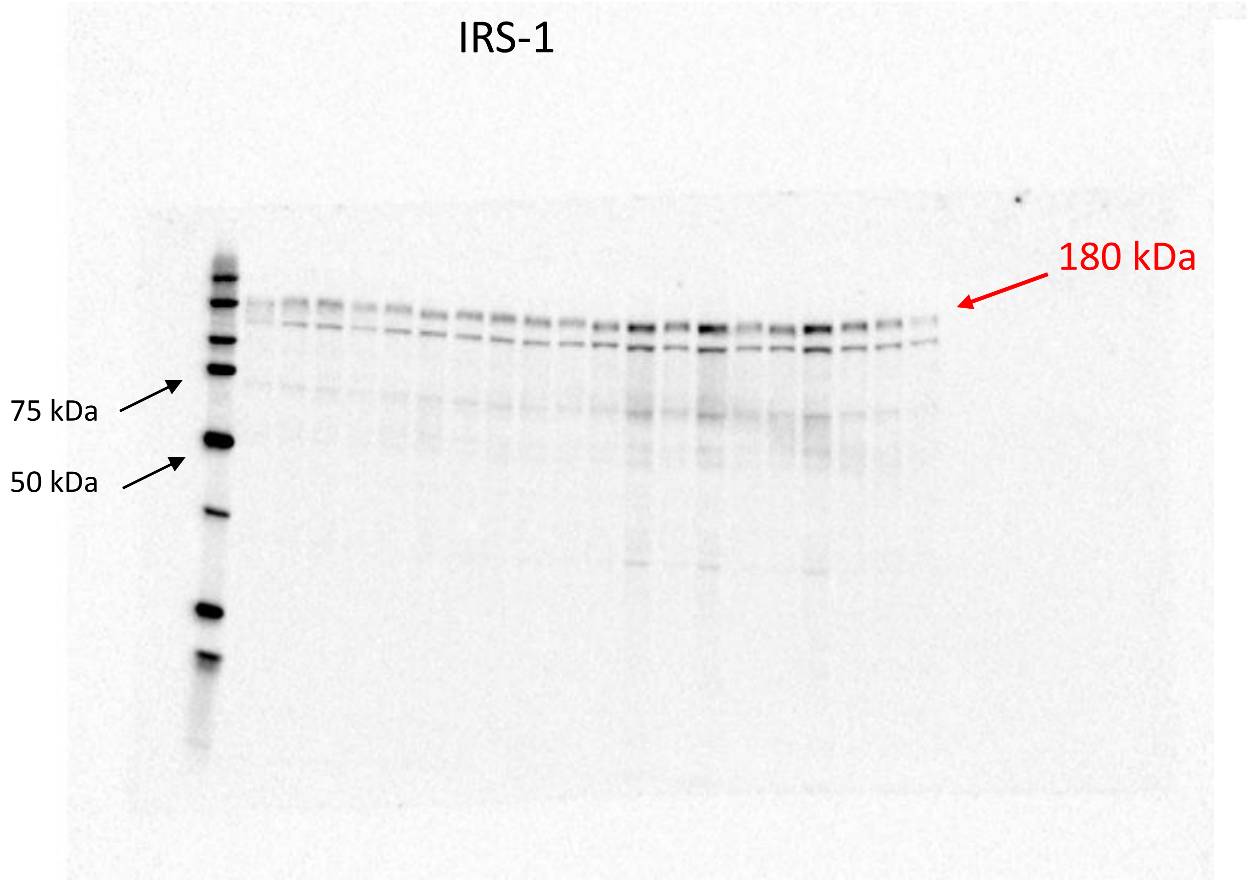

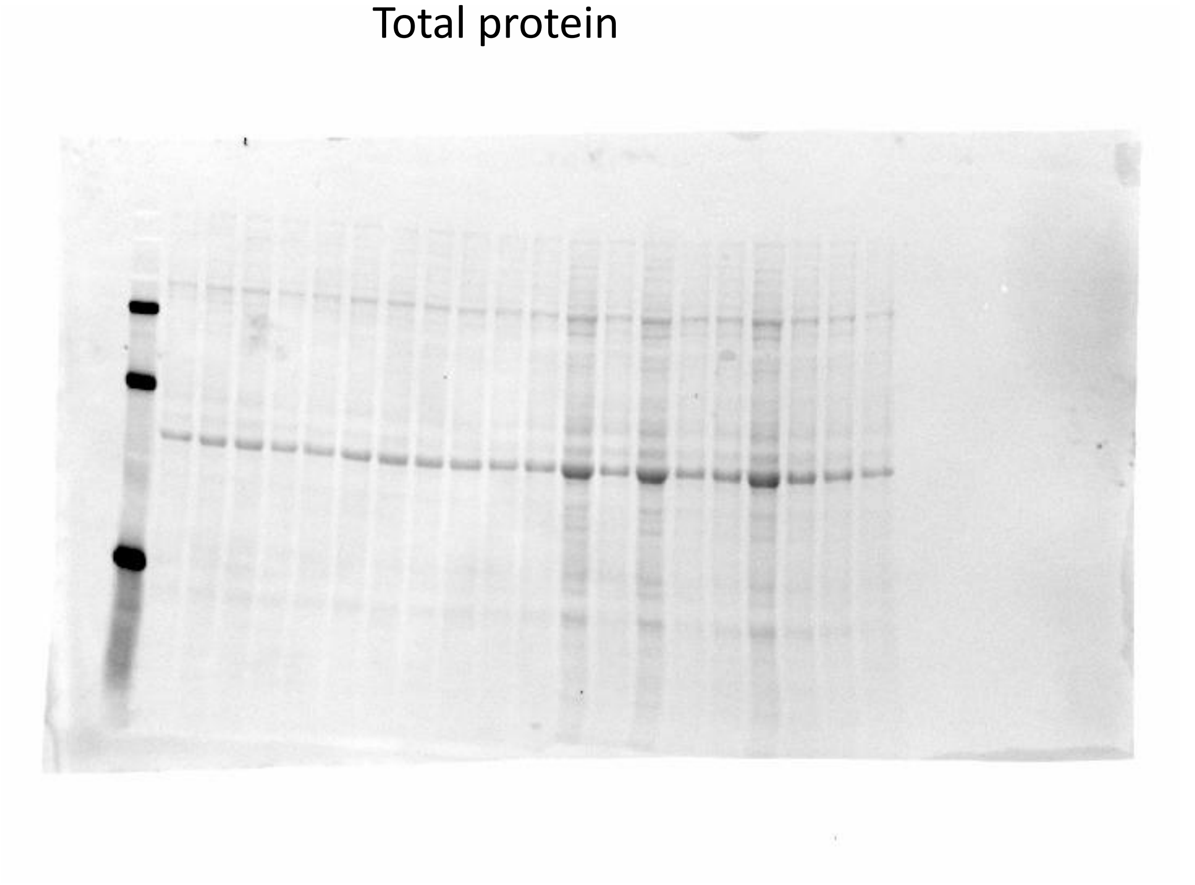

Loading order in all proteins: Control, INS, CBG+INS, PA+INS, PA+CBG+INS

Analysis was repeated and it was not used in the manuscript

pIRS-1 (Ser307)

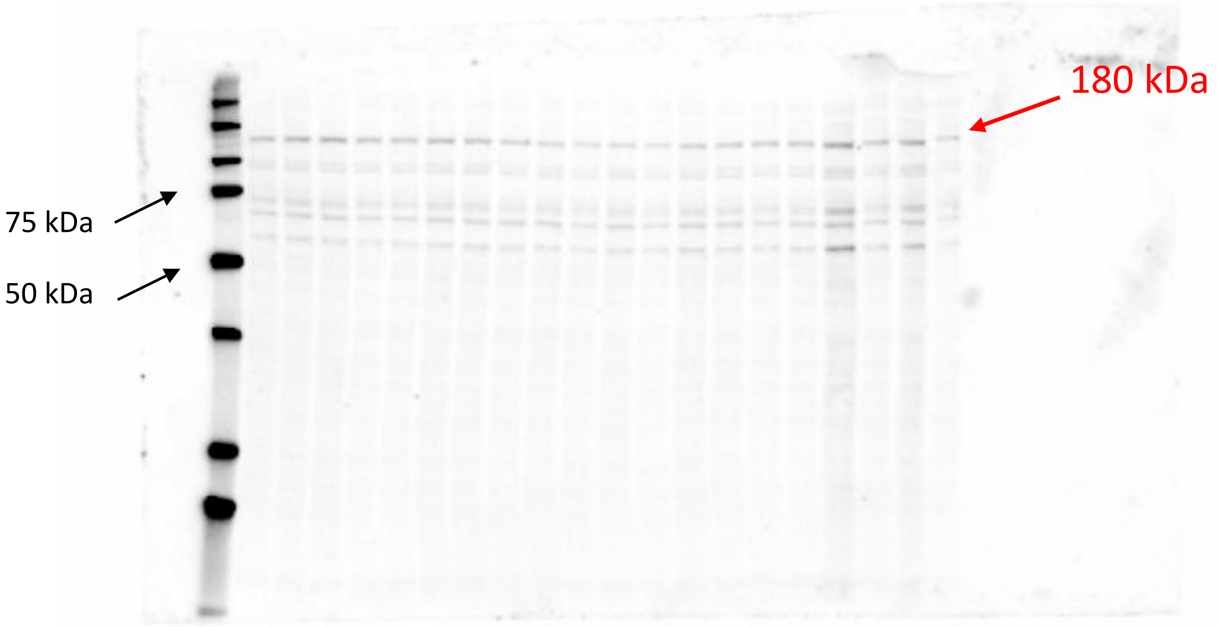

Total protein

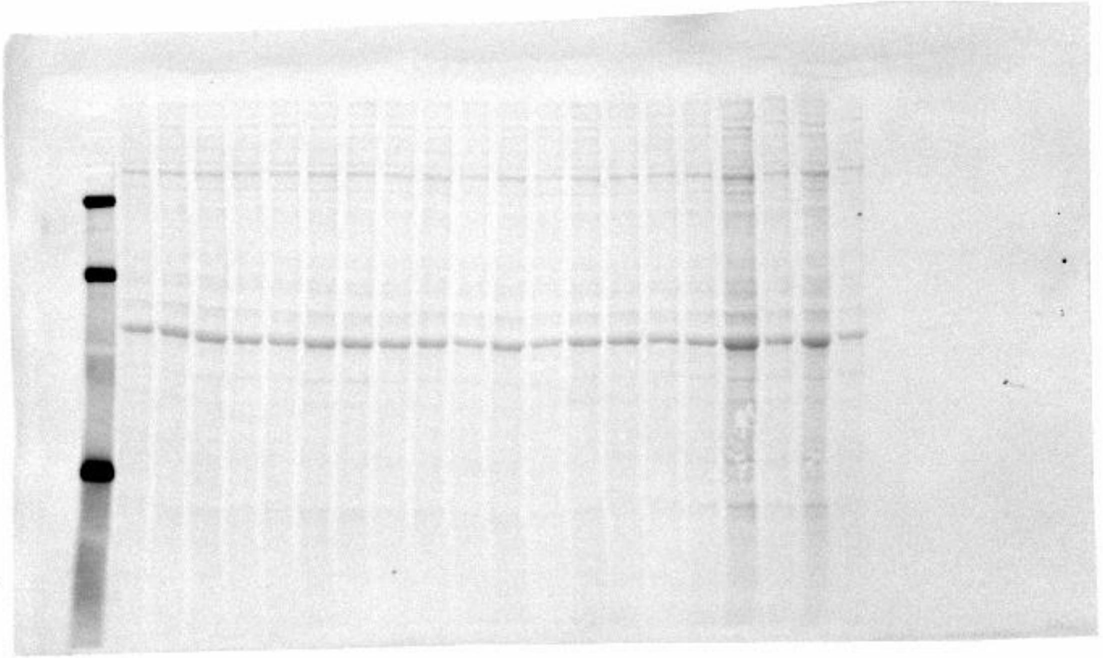

Loading order in all proteins: Control, INS, CBG+INS, PA+INS, PA+CBG+INS

pIRS-1 (Ser307)

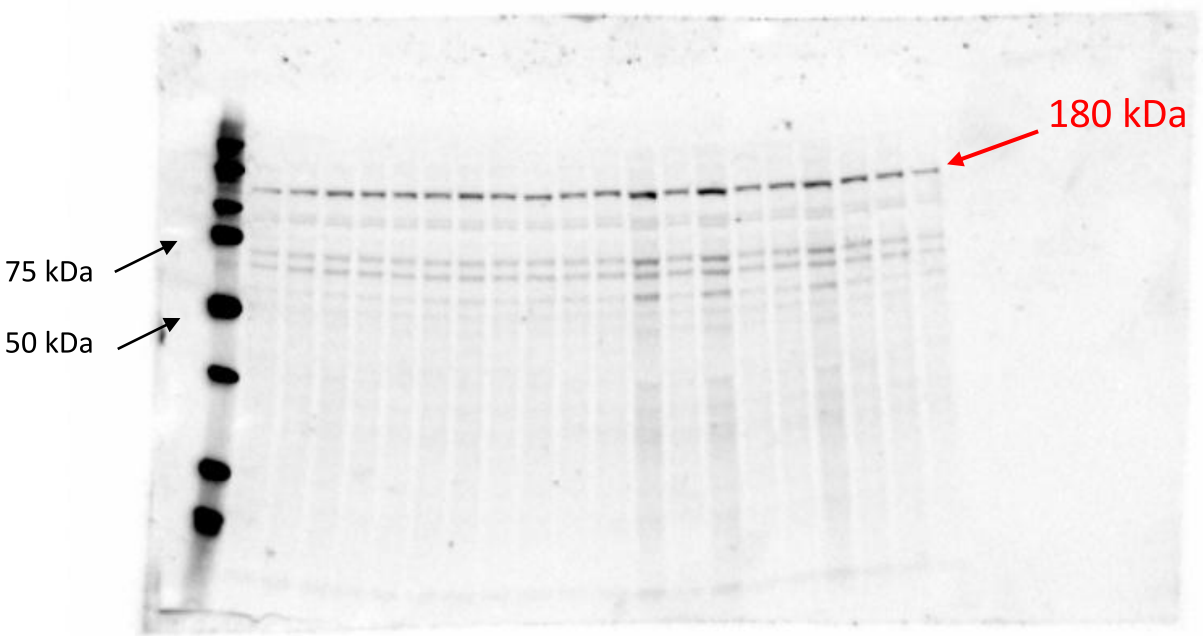

Total protein

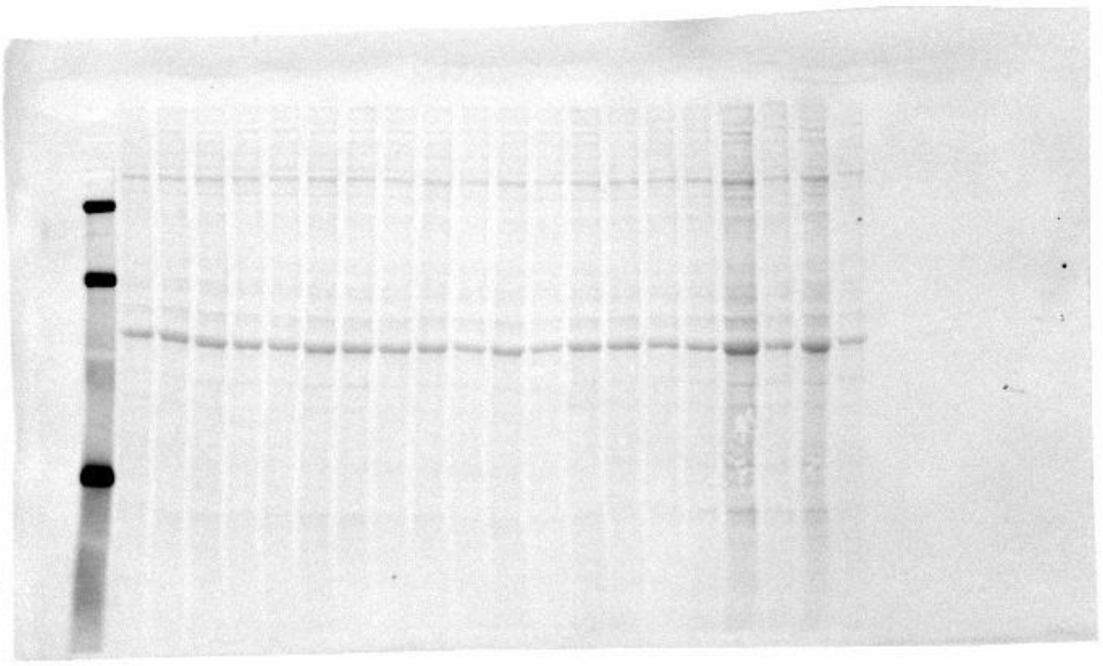

Loading order in all proteins: Control, INS, CBG+INS, PA+INS, PA+CBG+INS

Analysis was repeated and it was not used in the manuscript

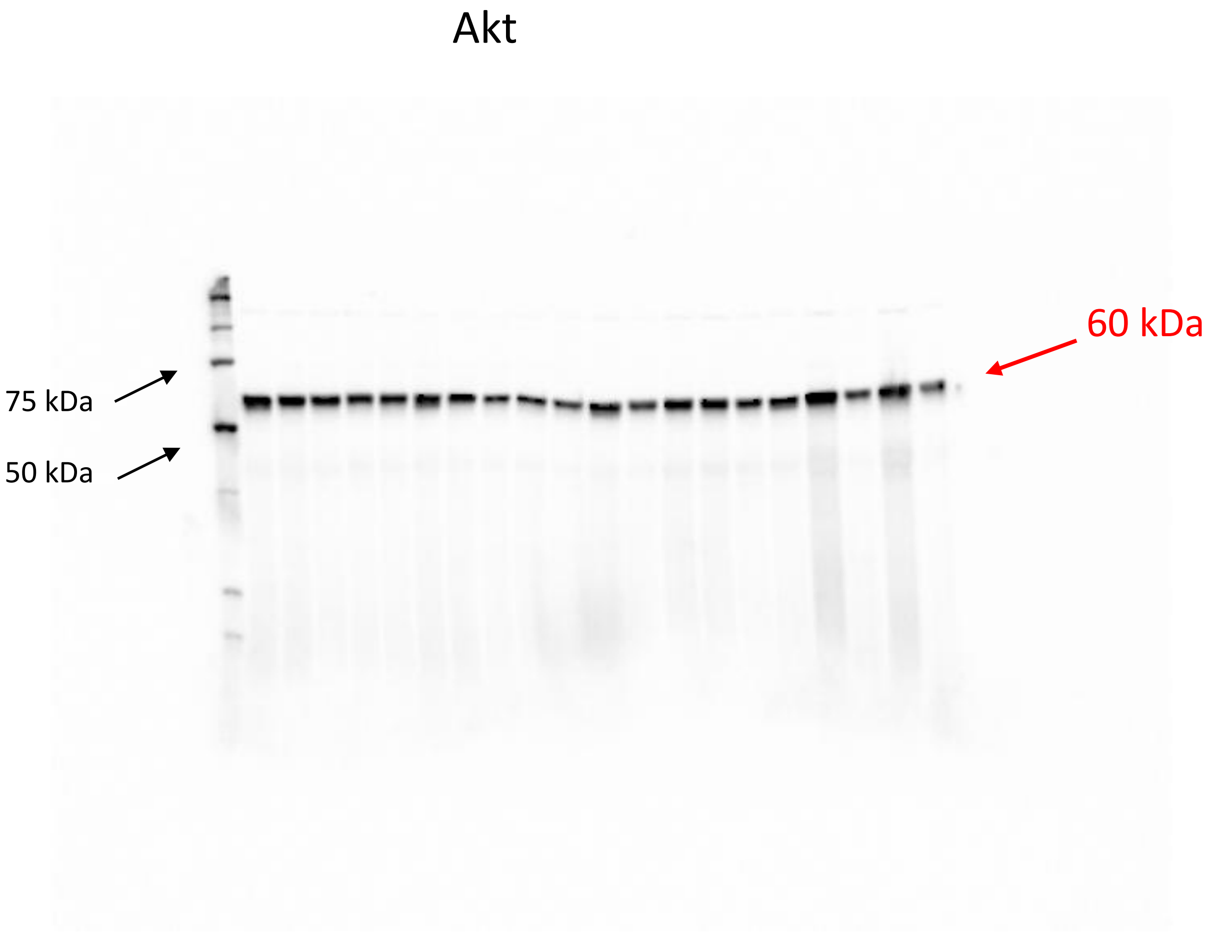

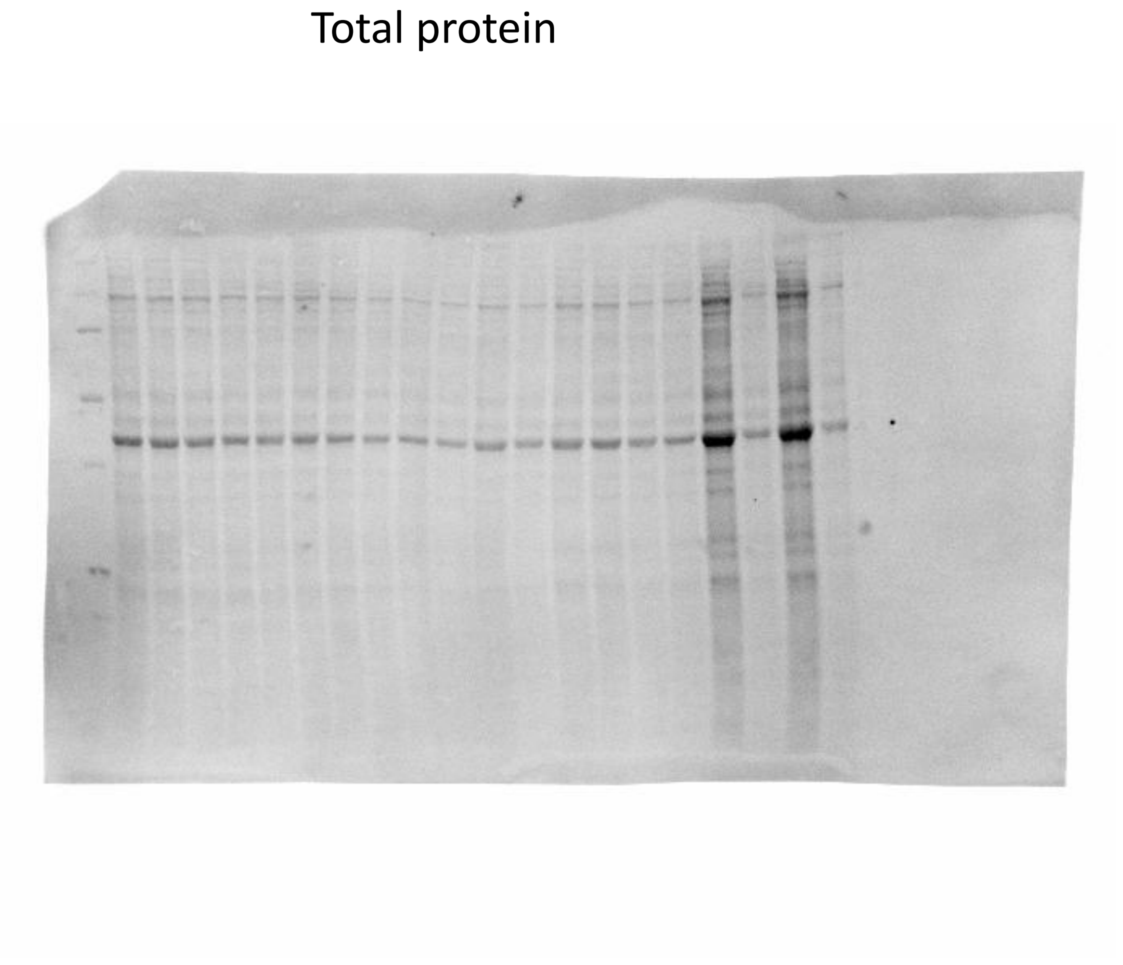

Loading order in all proteins: Control, INS, CBG+INS, PA+INS, PA+CBG+INS

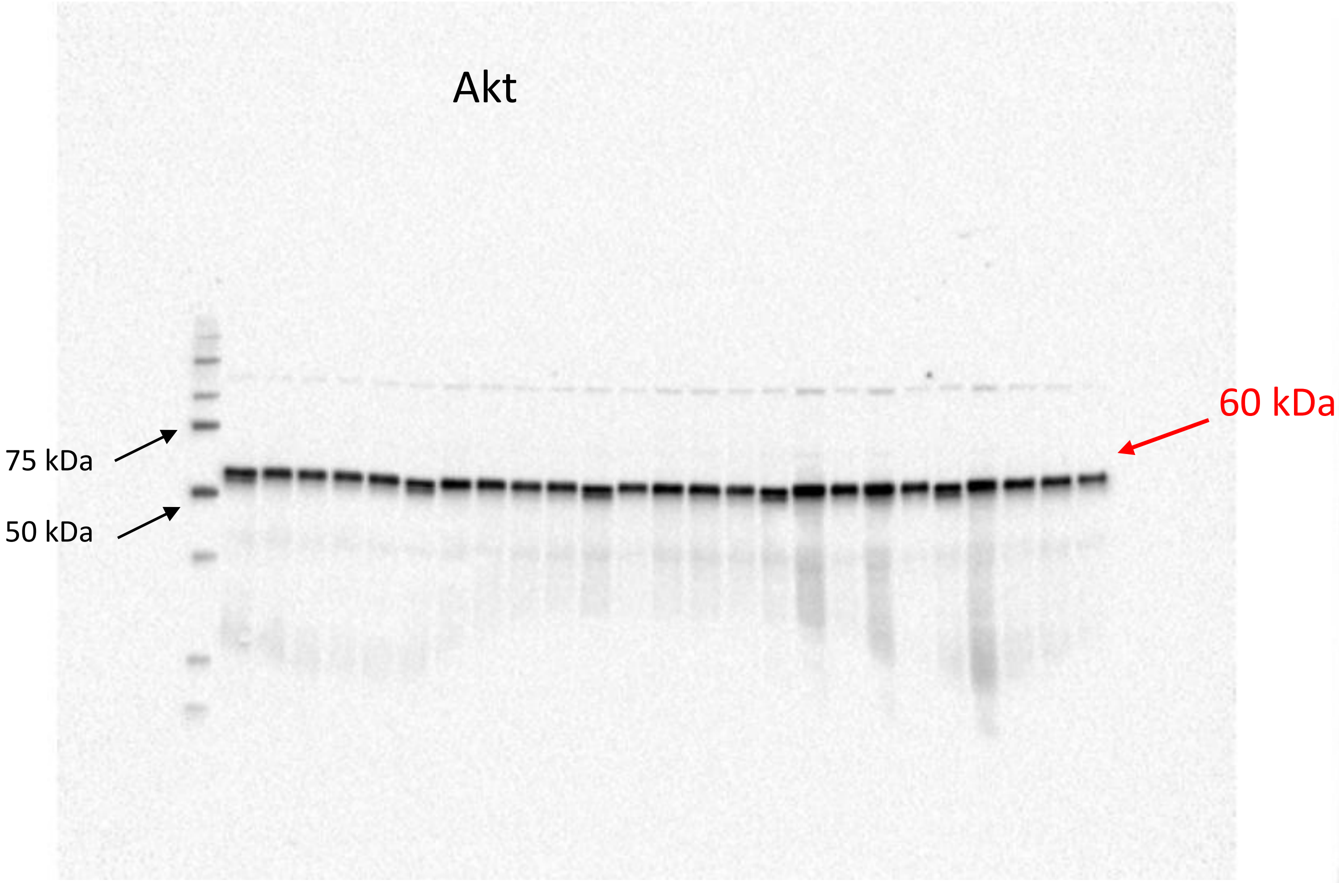

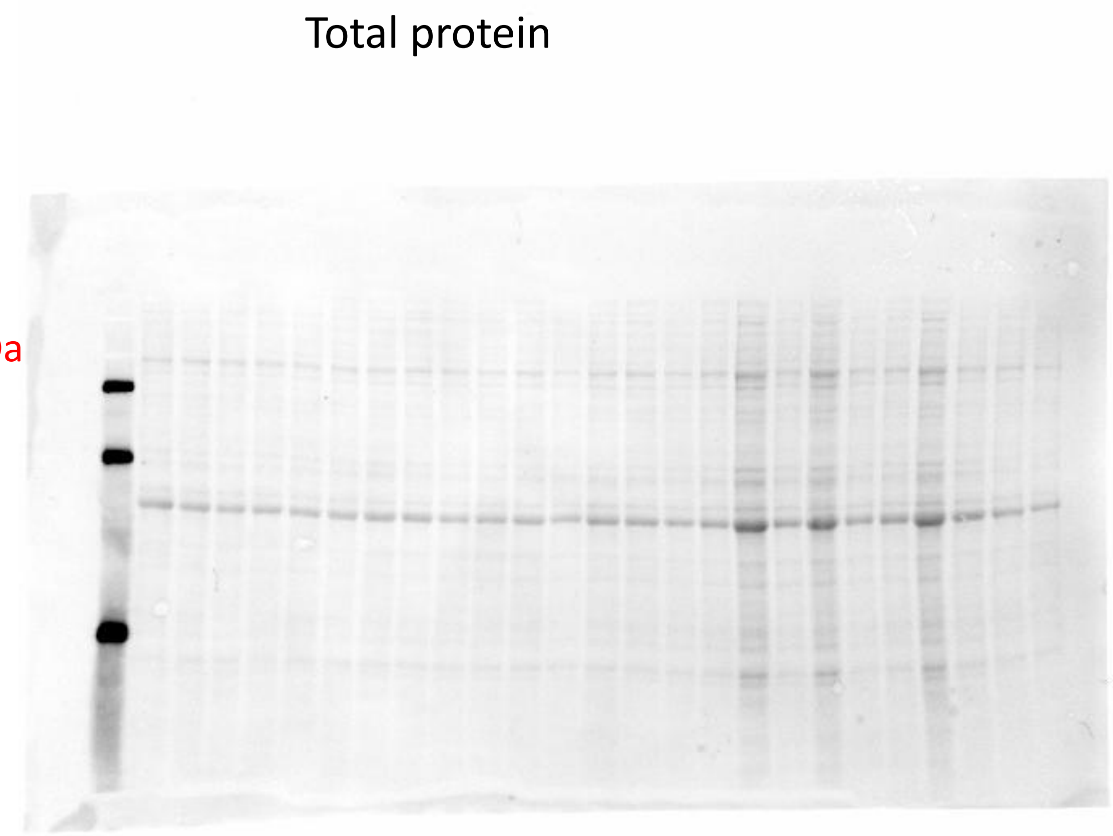

Loading order in all proteins: Control, INS, CBG+INS, PA+INS, PA+CBG+INS

Analysis was repeated and it was not used in the manuscript

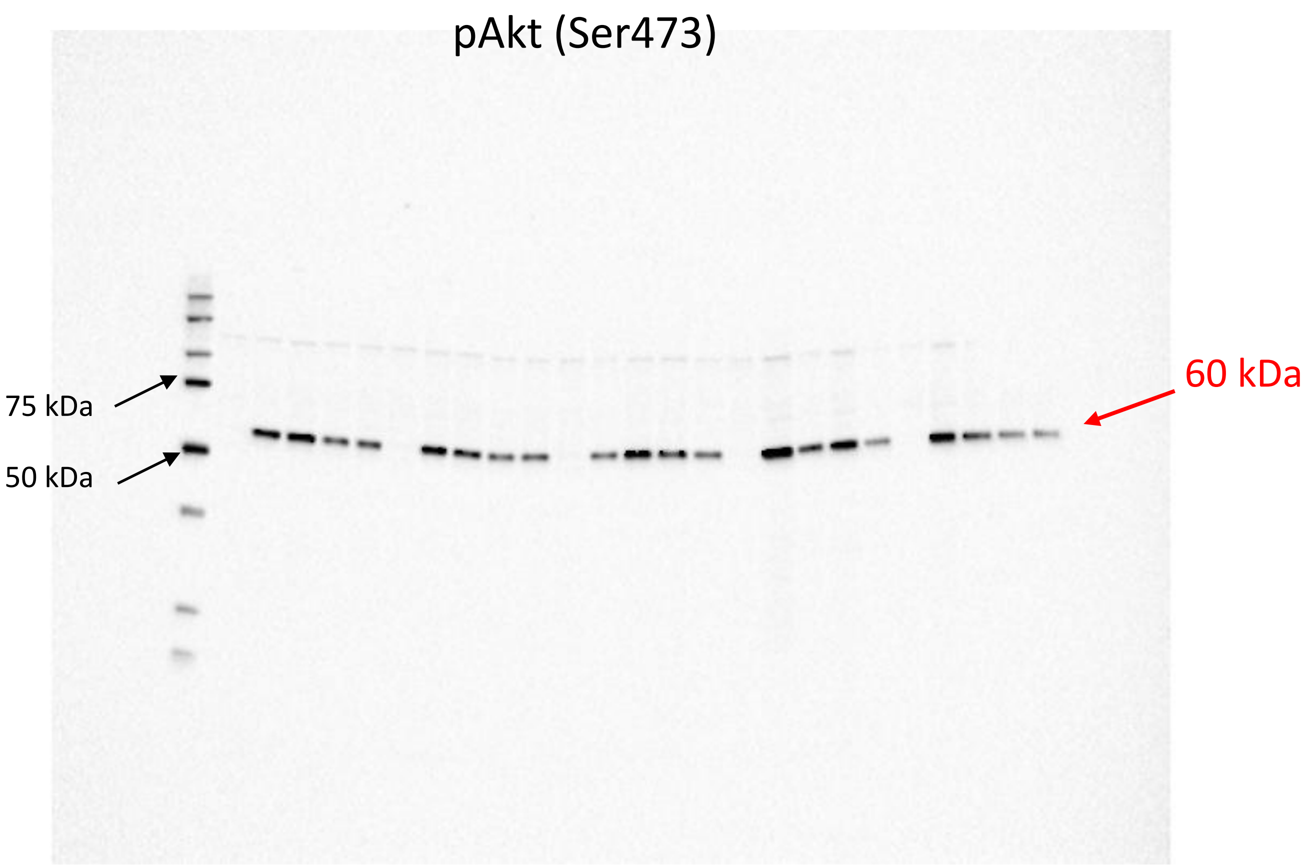

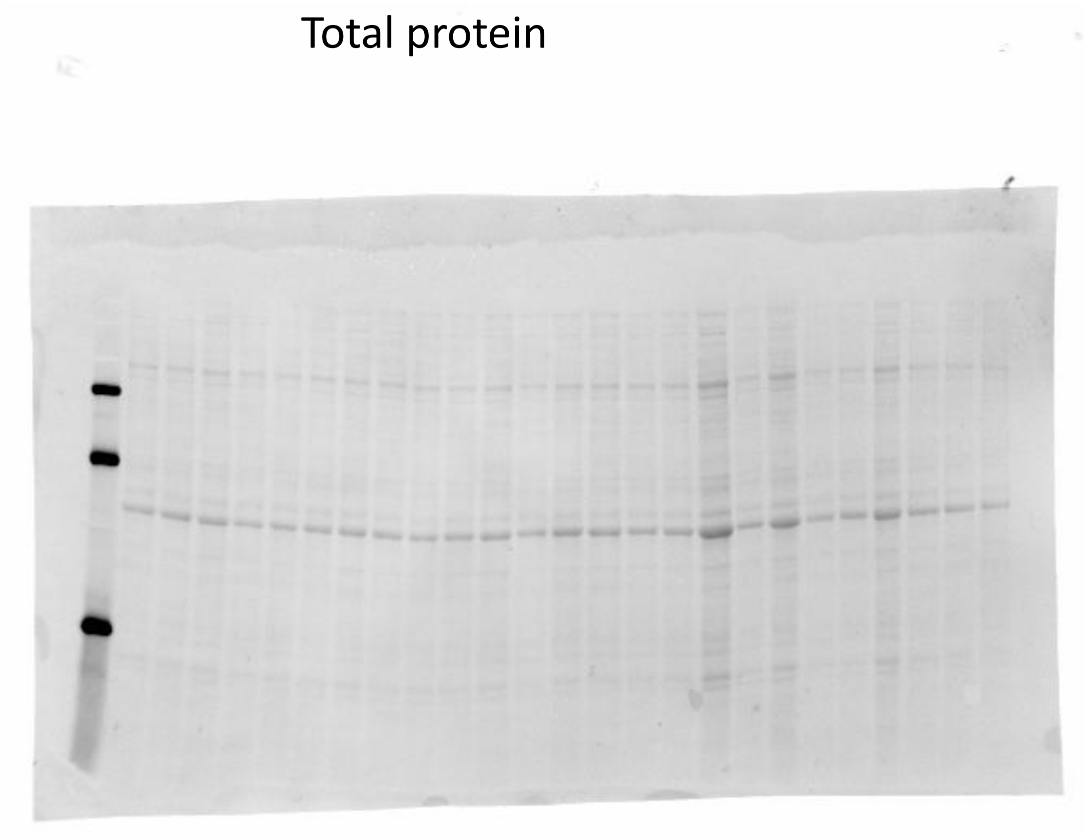

Loading order in all proteins: Control, INS, CBG+INS, PA+INS, PA+CBG+INS

pAkt (Ser473)

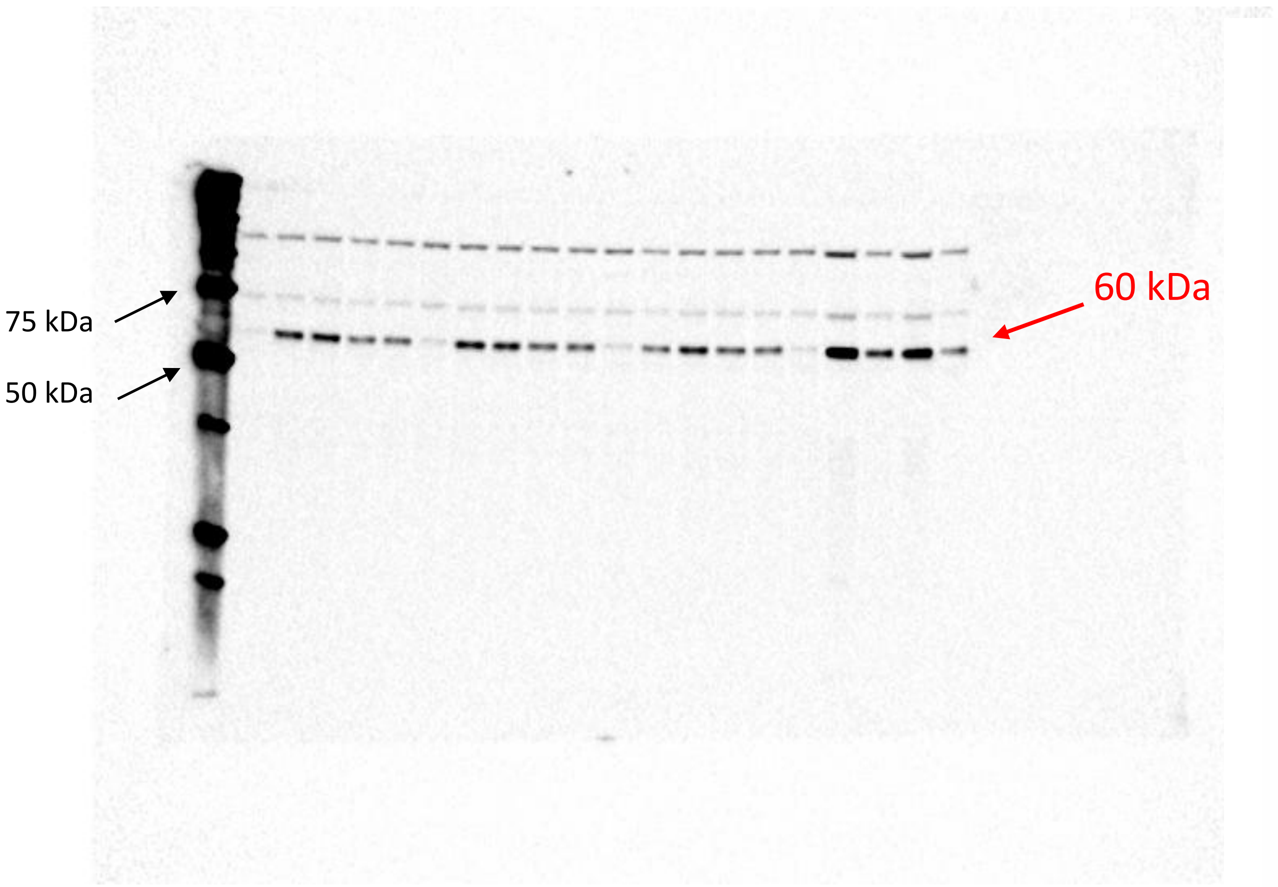

Total protein

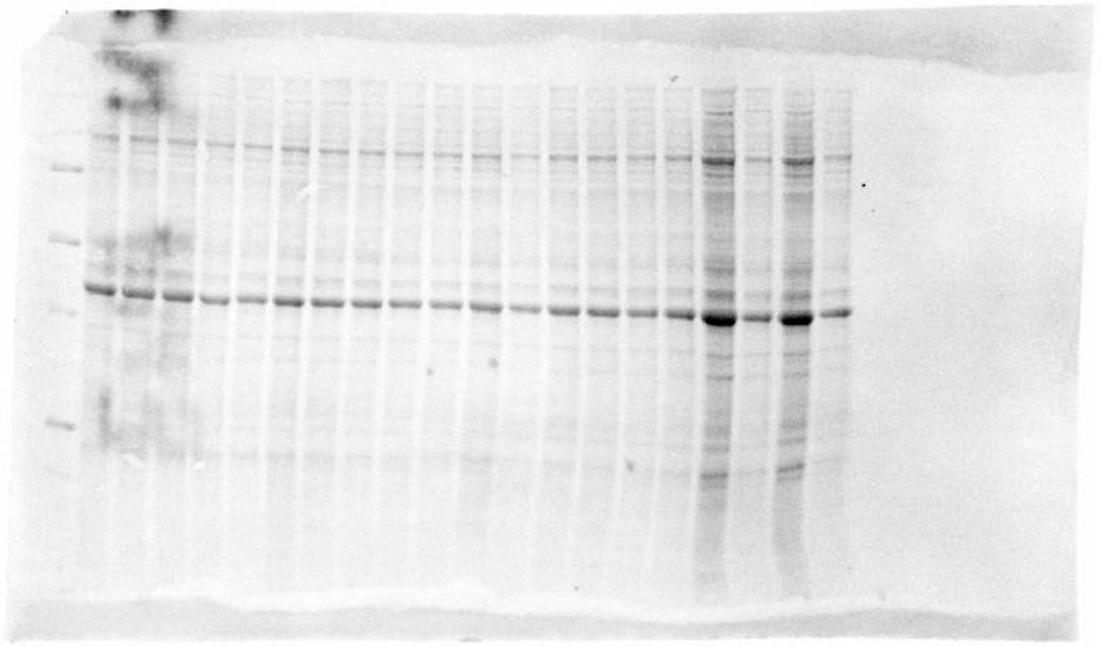

Loading order in all proteins: Control, INS, CBG+INS, PA+INS, PA+CBG+INS

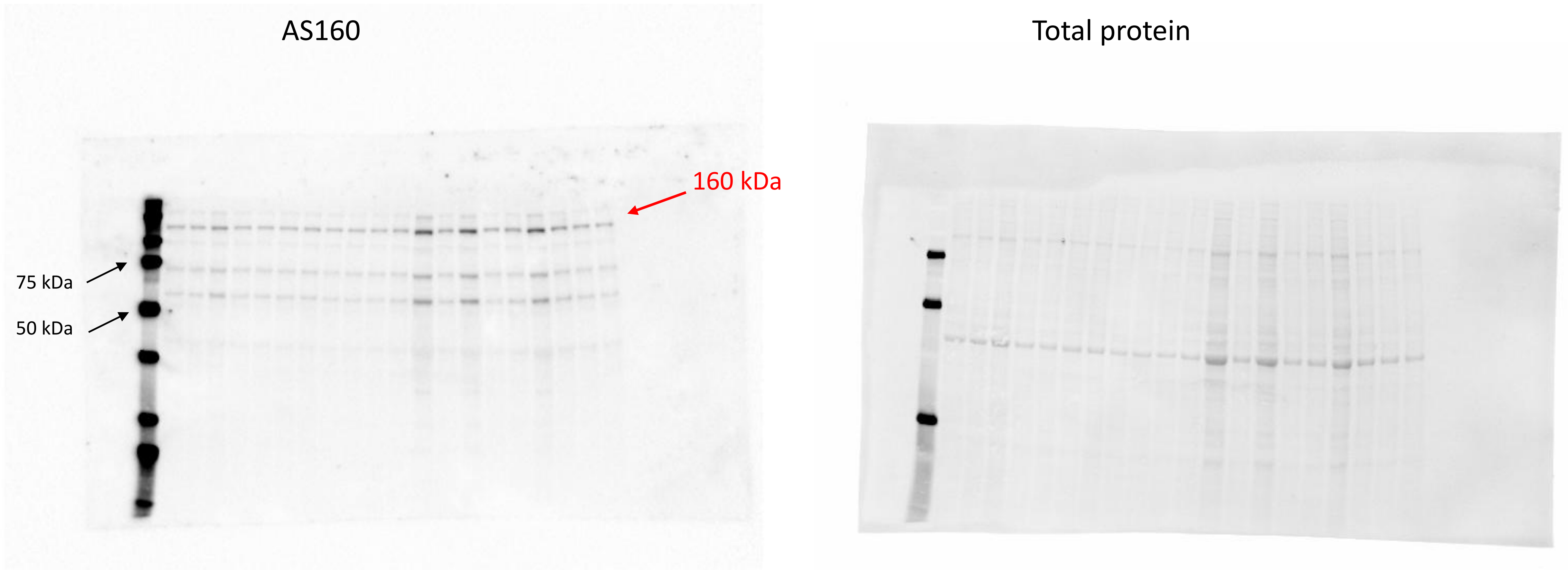

Loading order in all proteins: Control, INS, CBG+INS, PA+INS, PA+CBG+INS

pAS160 (Thr642)

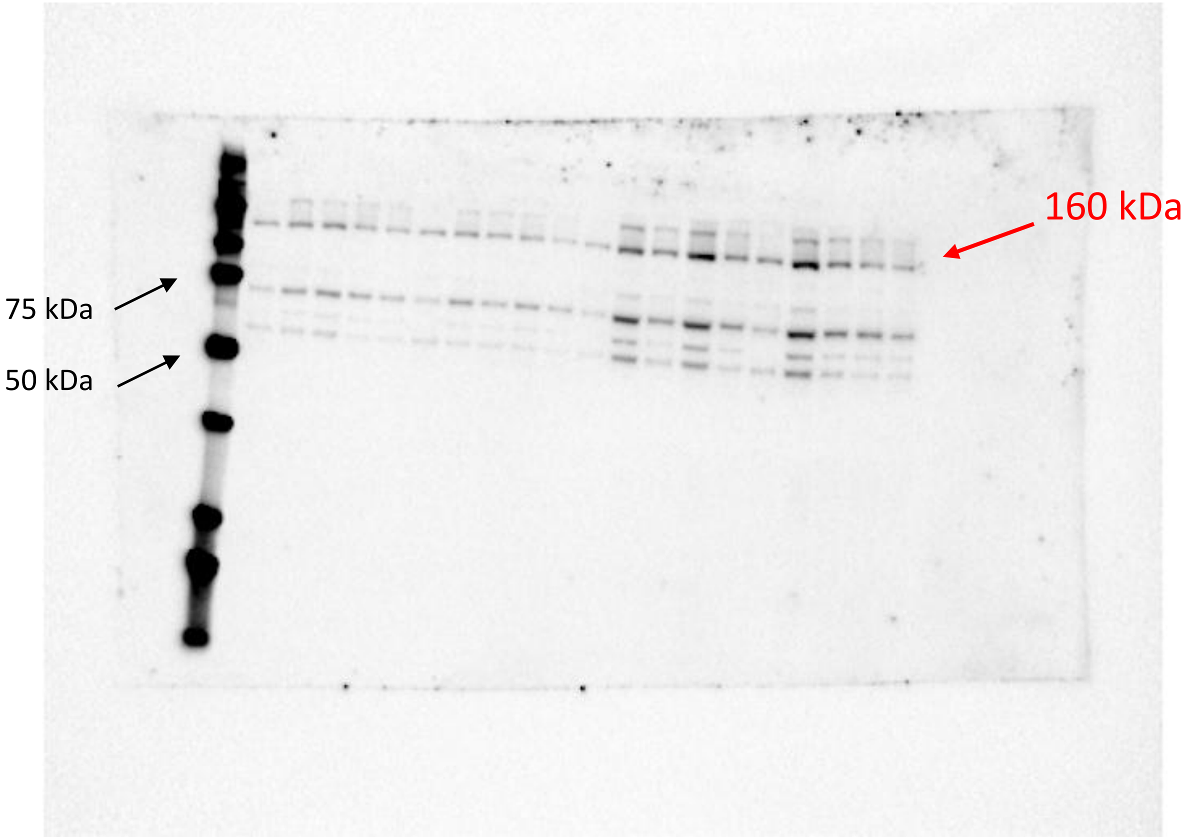

Total protein

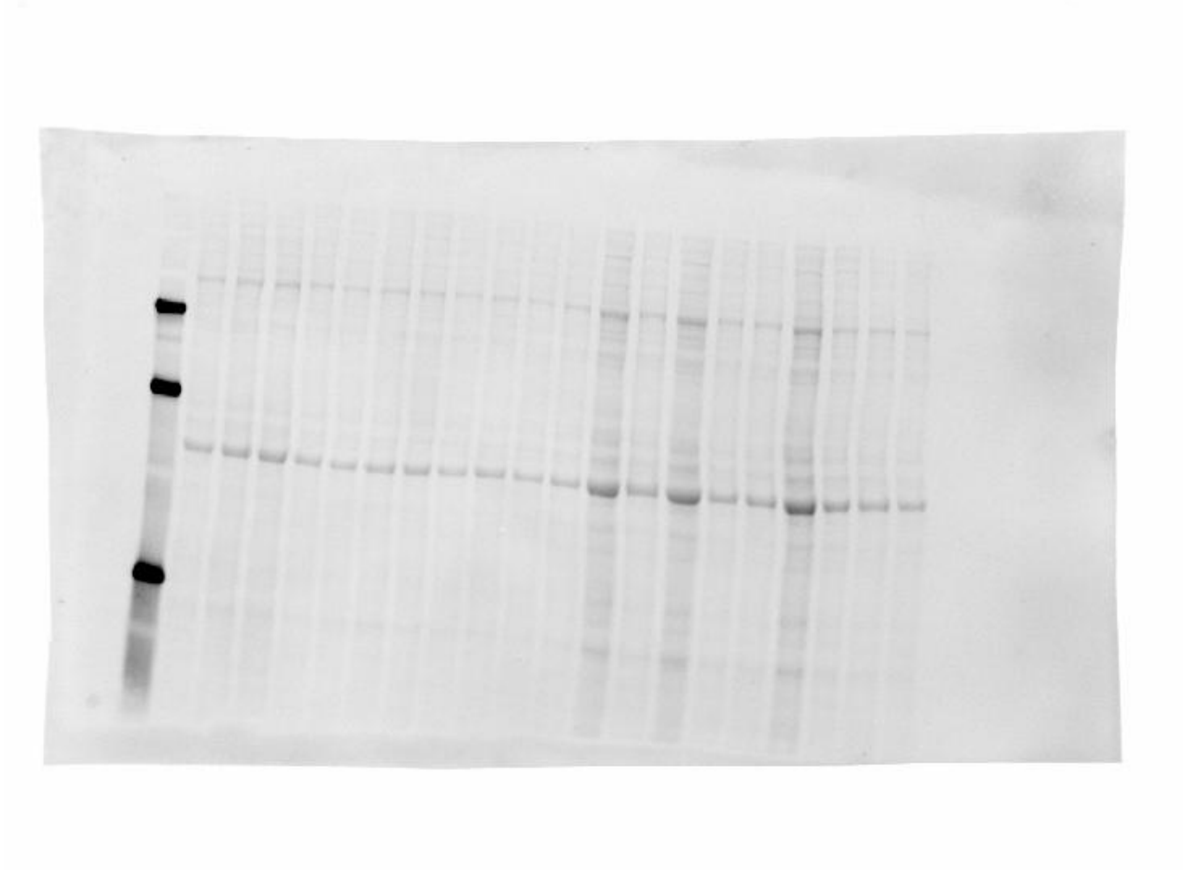

Loading order in all proteins: Control, INS, CBG+INS, PA+INS, PA+CBG+INS

GLUT-4

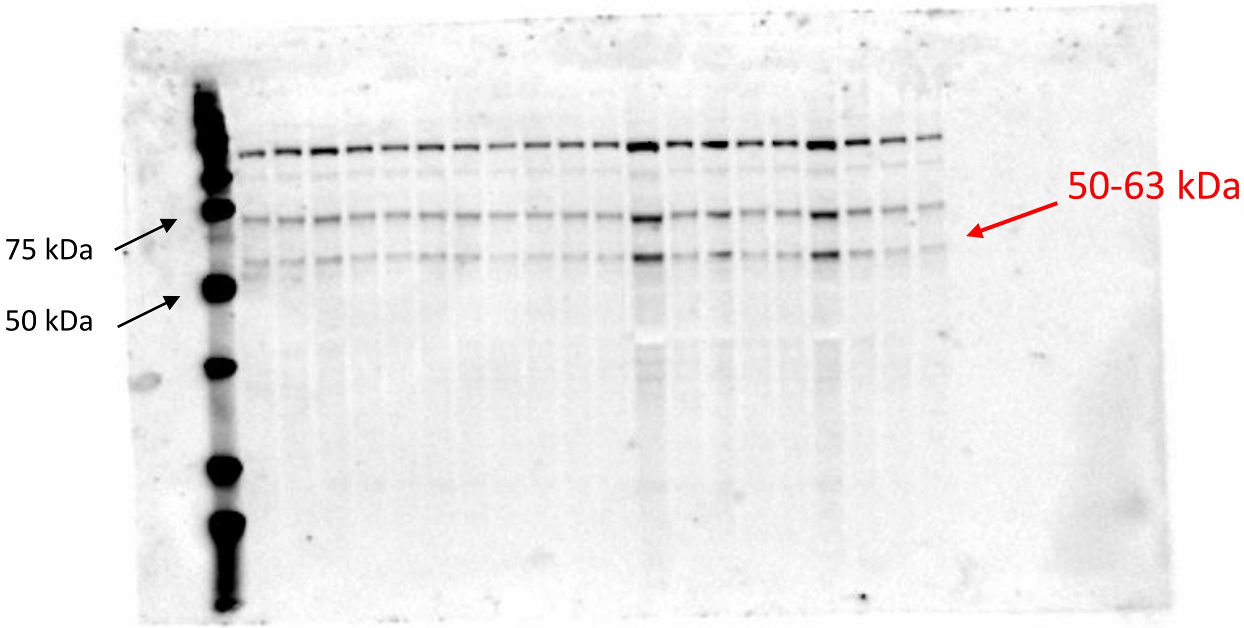

Total protein

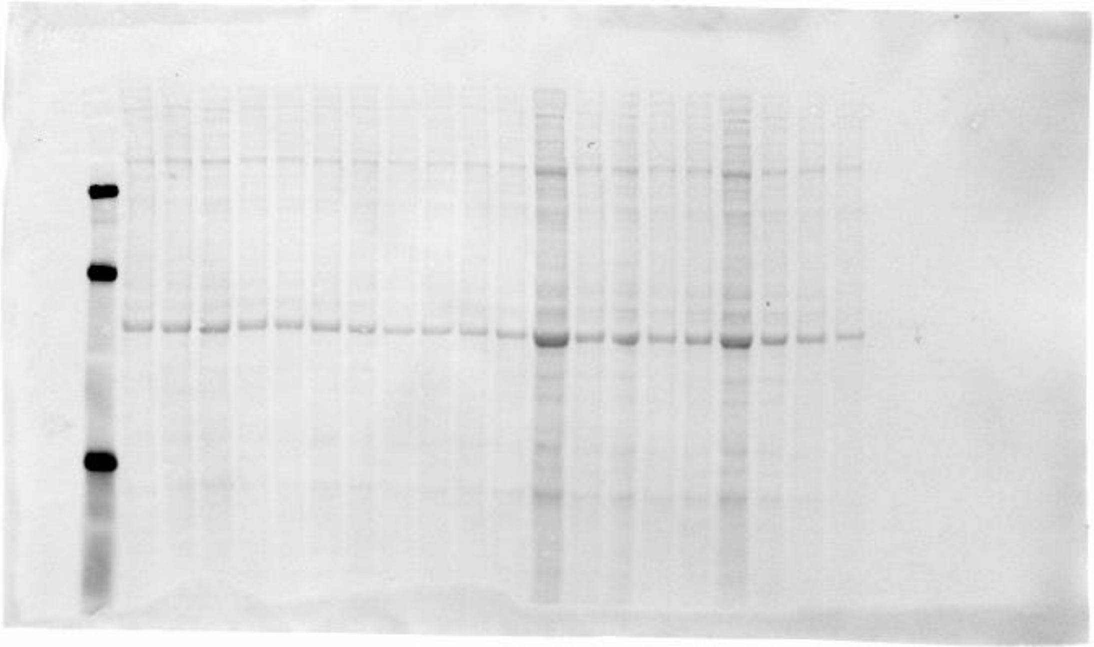

Loading order in all proteins: Control, INS, CBG+INS, PA+INS, PA+CBG+INS

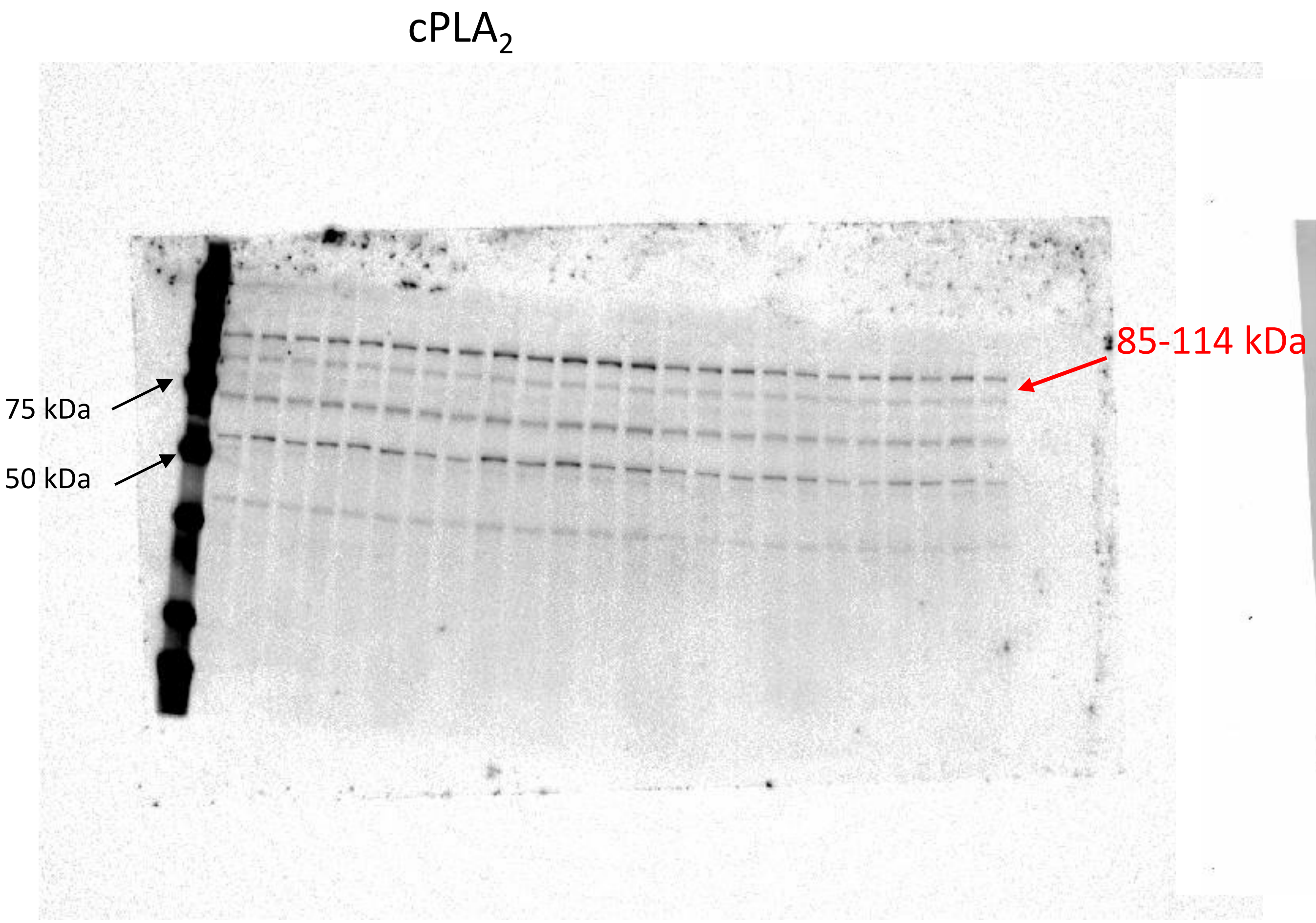

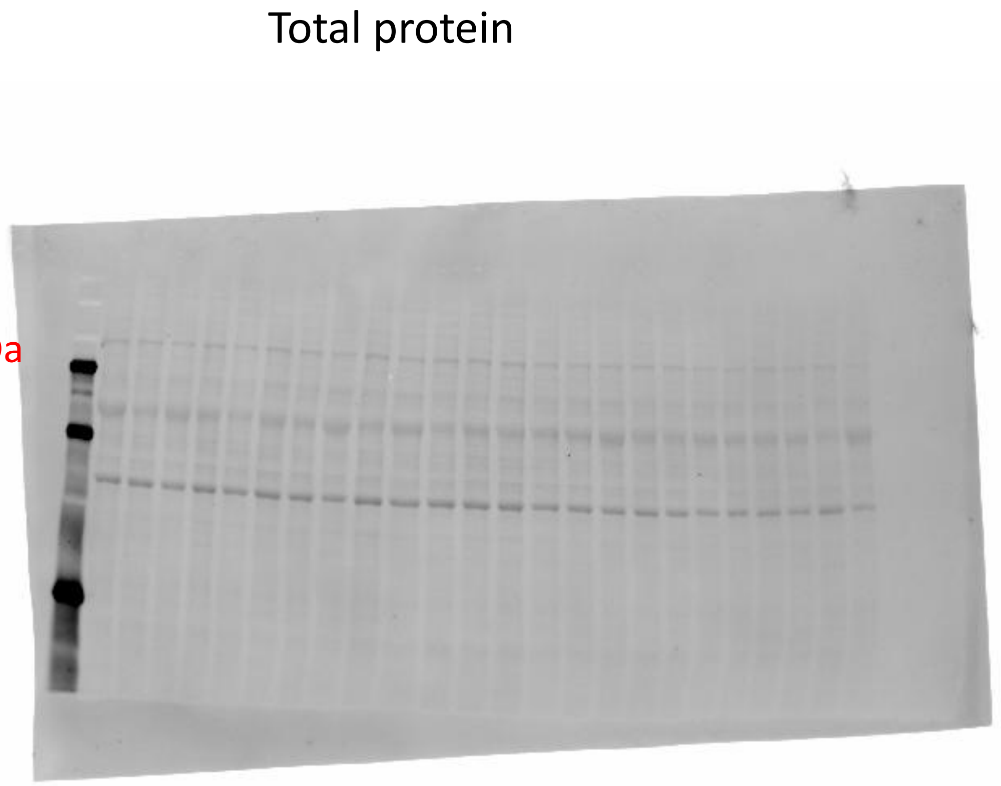

Loading order in all proteins: Control, INS, CBG+INS, PA+INS, PA+CBG+INS

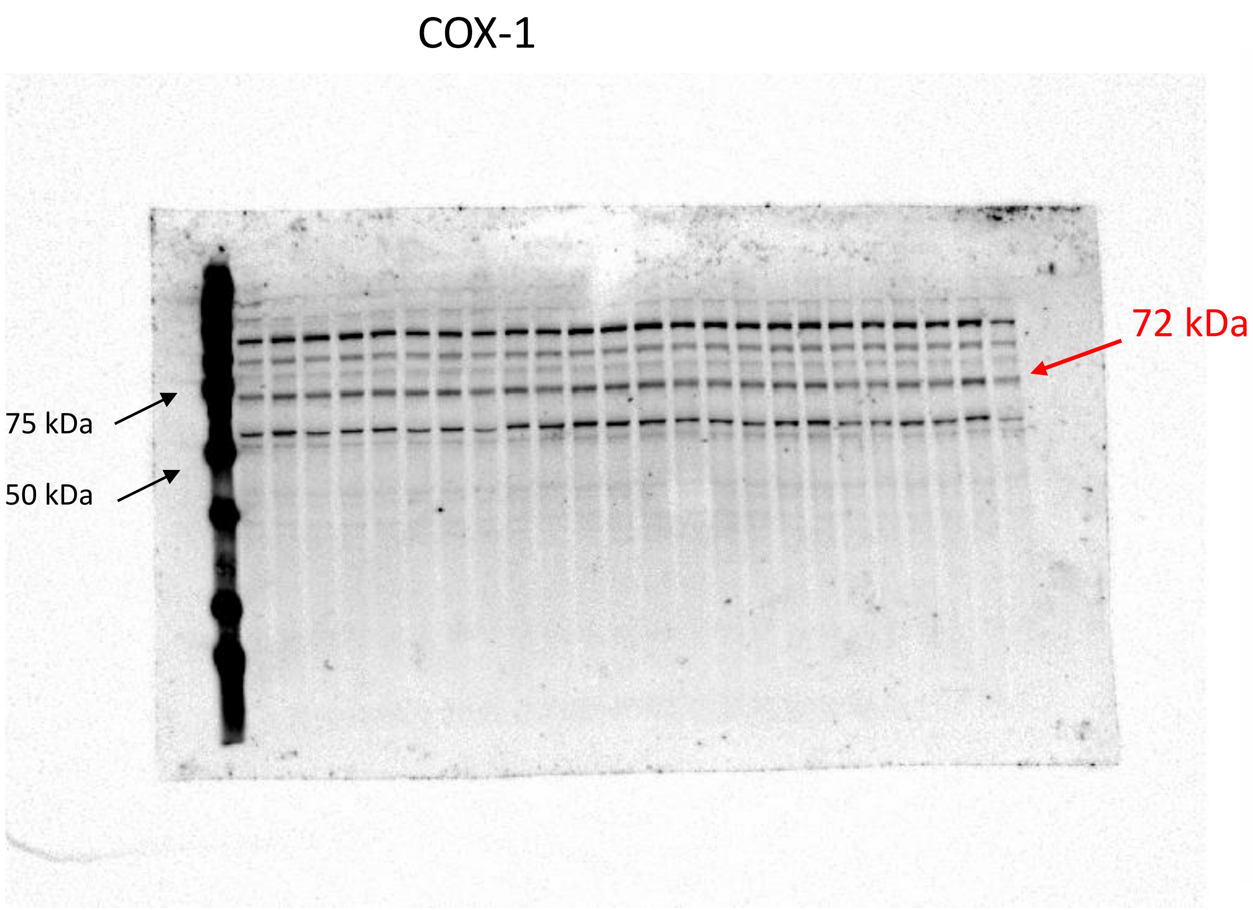

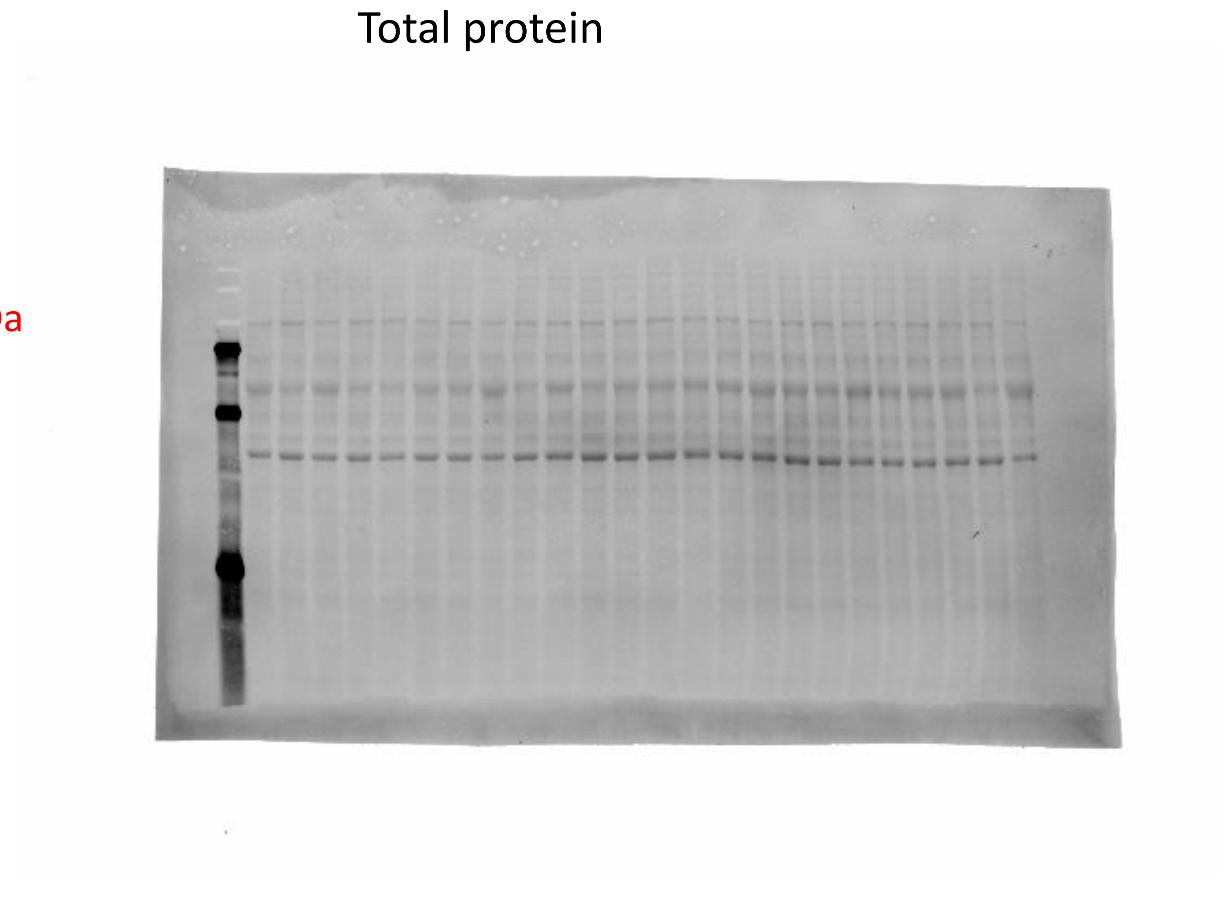

Loading order in all proteins: Control, INS, CBG+INS, PA+INS, PA+CBG+INS

COX-2

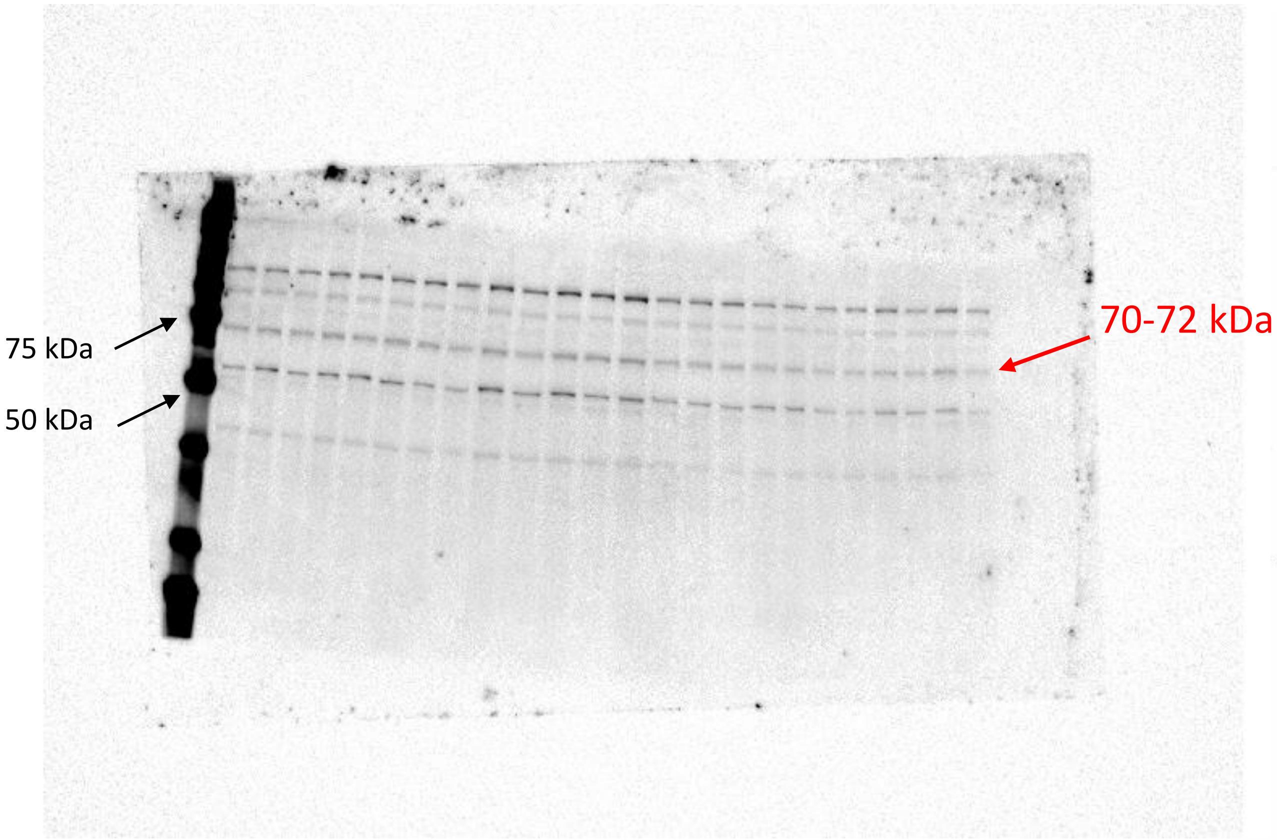

Total protein

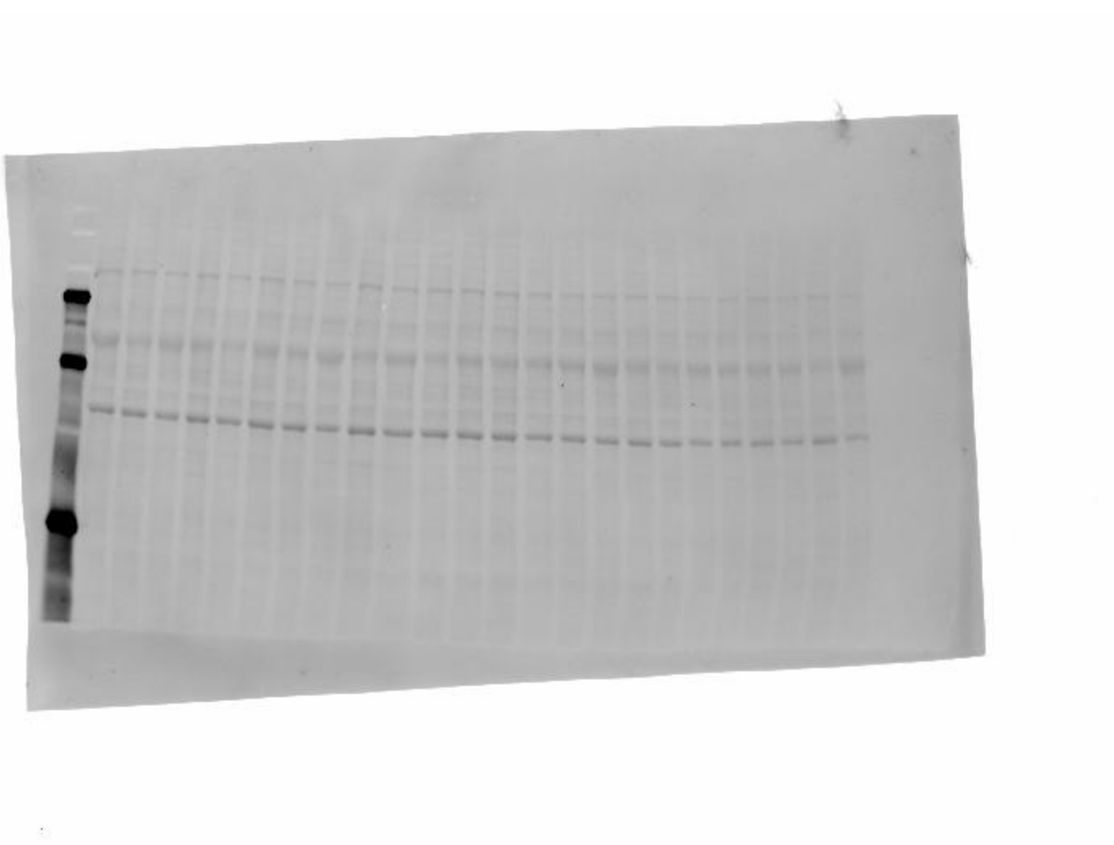

Loading order in all proteins: Control, INS, CBG+INS, PA+INS, PA+CBG+INS

5-LOX

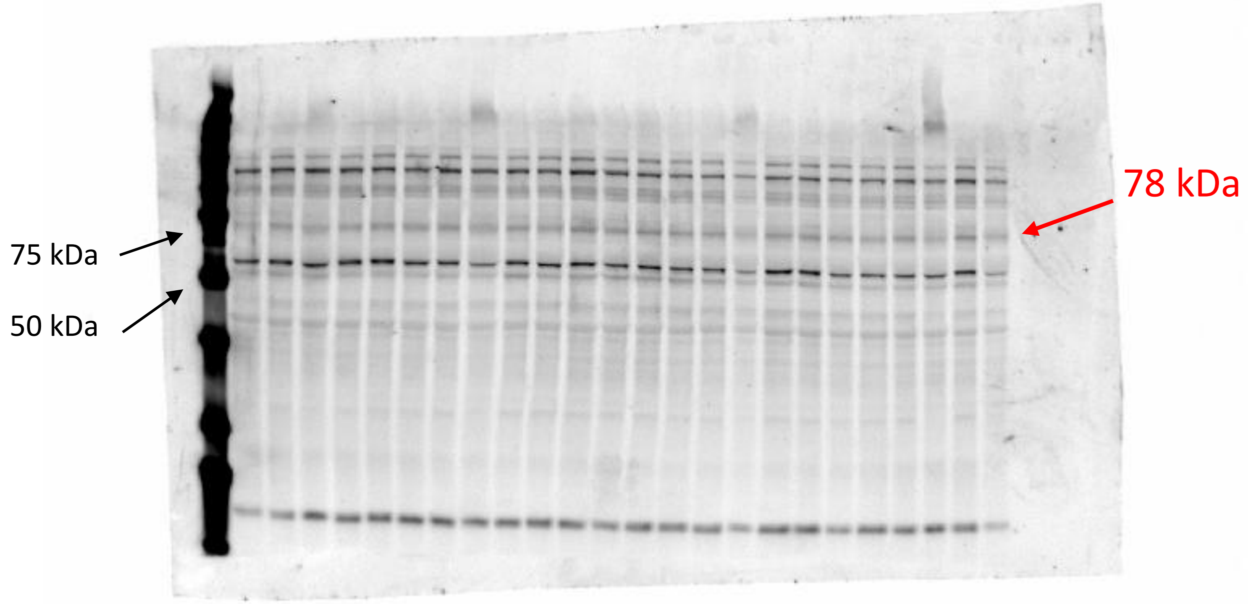

Total protein

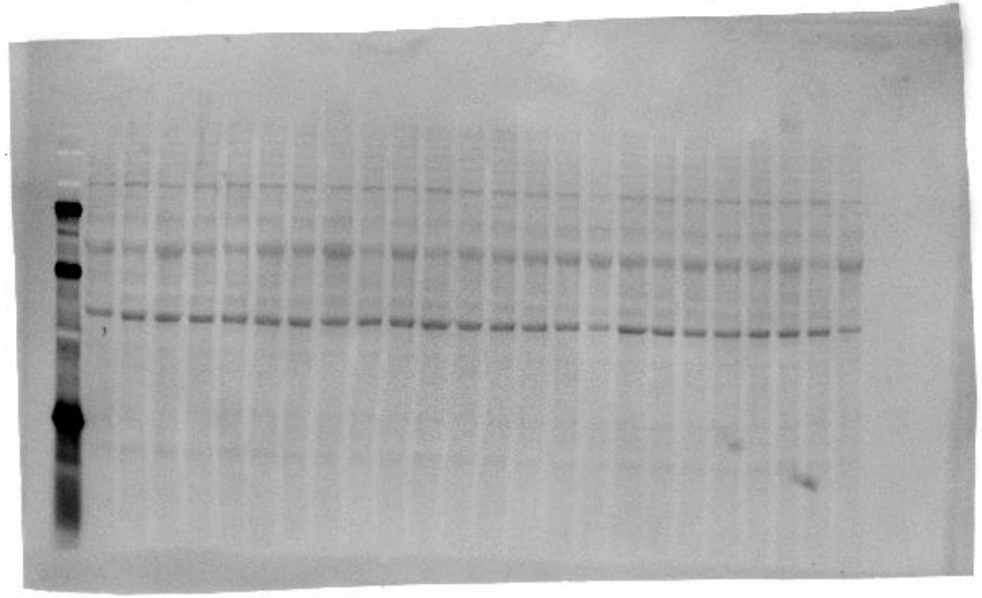

Loading order in all proteins: Control, INS, CBG+INS, PA+INS, PA+CBG+INS

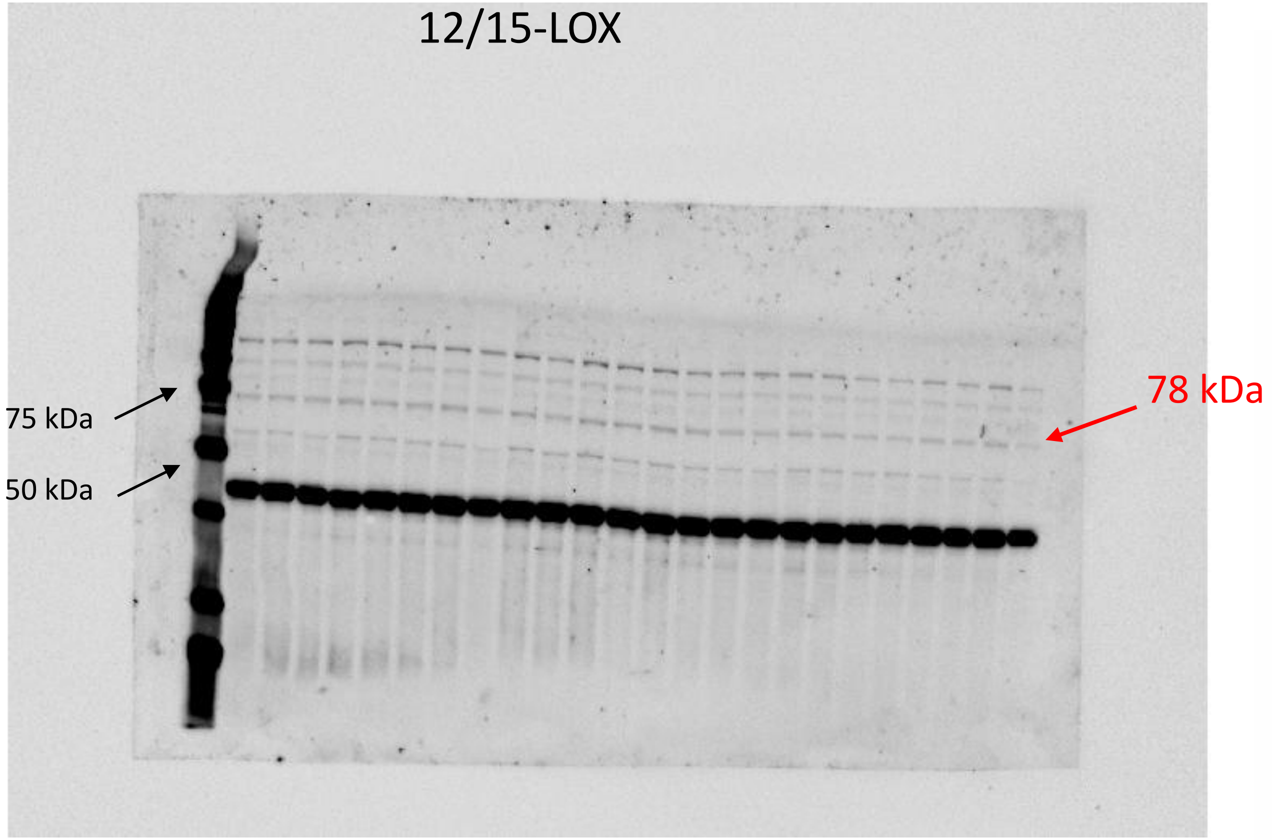

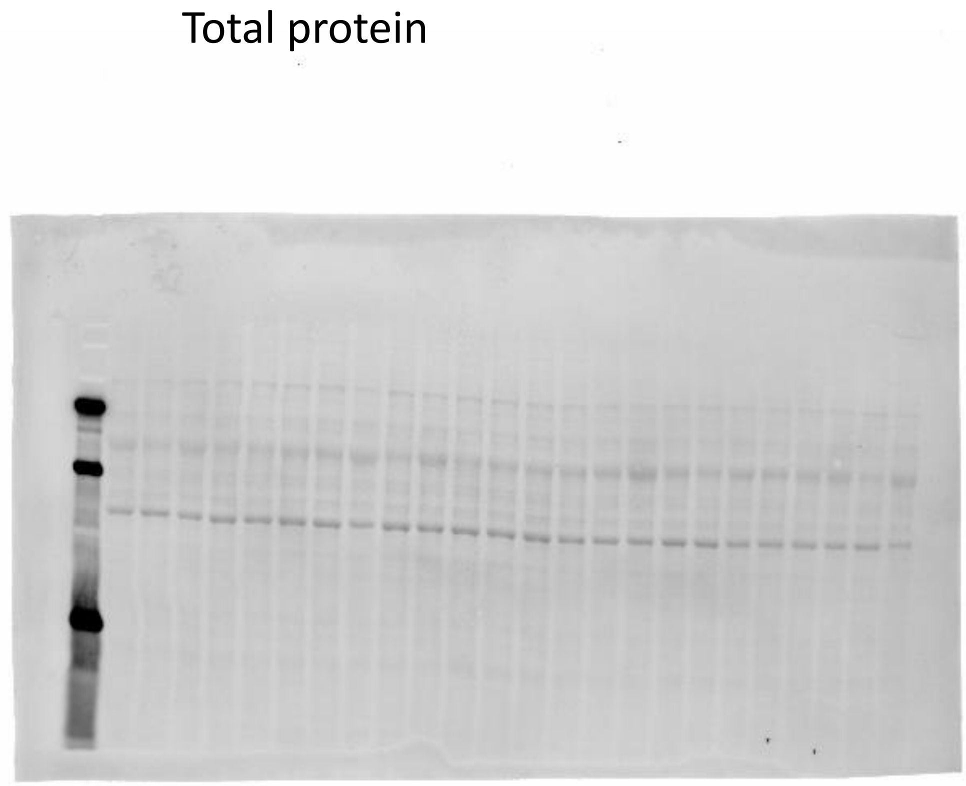

Loading order in all proteins: Control, INS, CBG+INS, PA+INS, PA+CBG+INS

CB$_1$

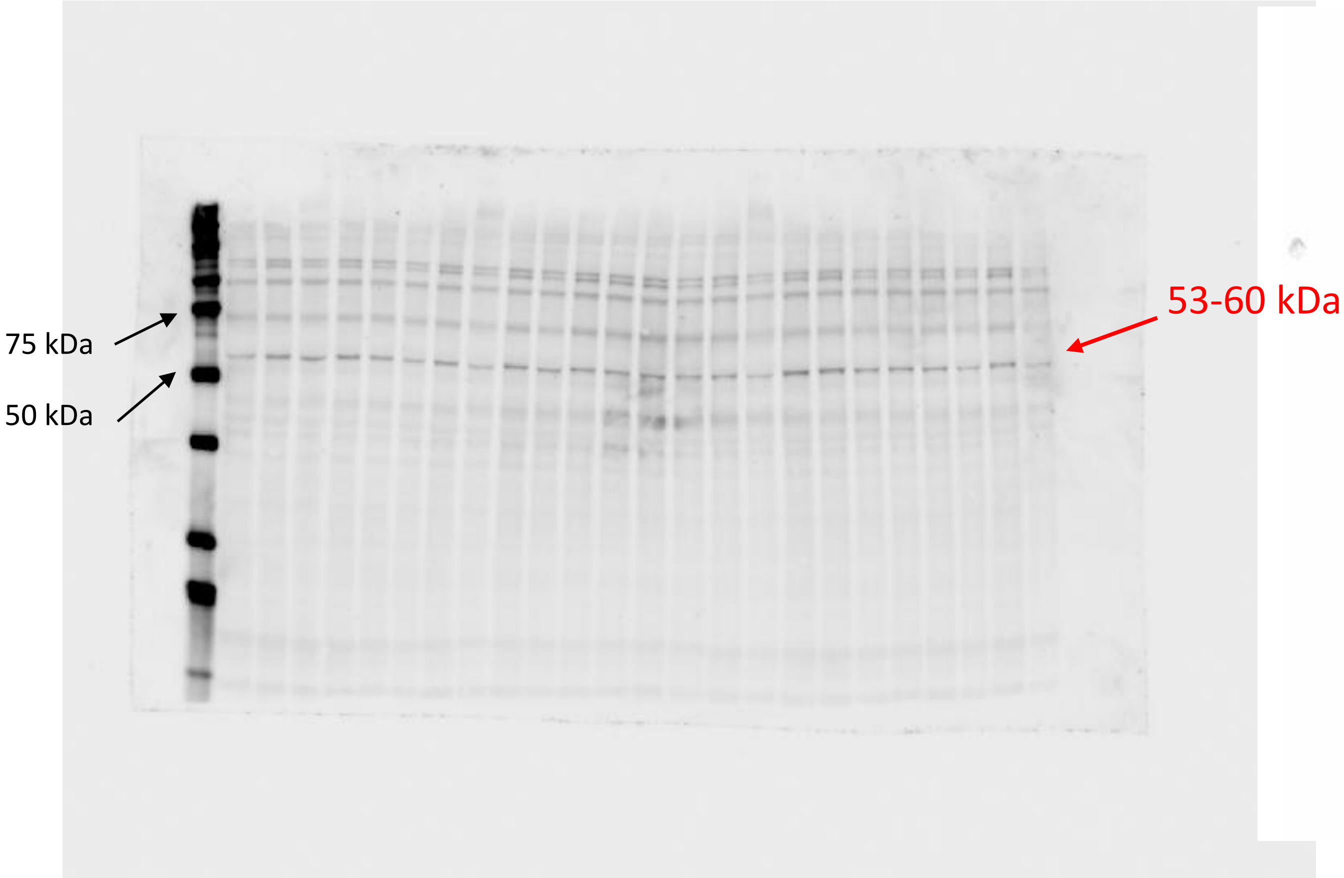

Total protein

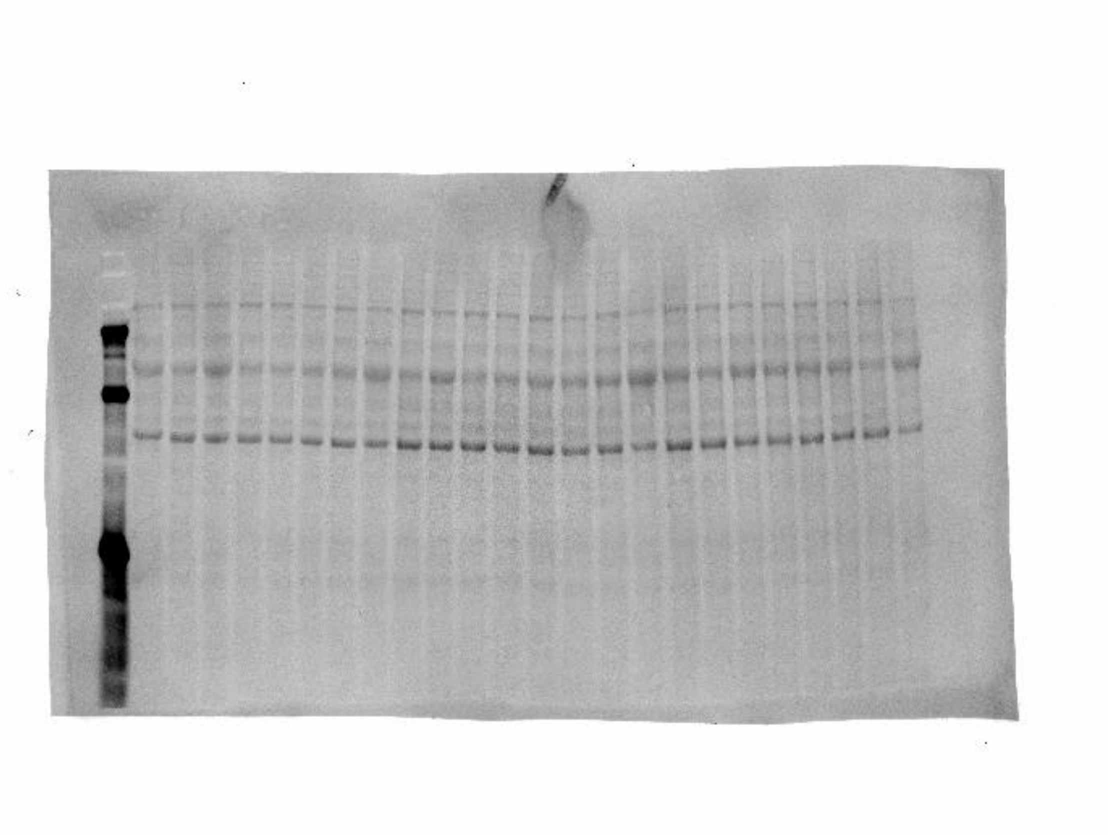

Loading order in all proteins: Control, INS, CBG+INS, PA+INS, PA+CBG+INS

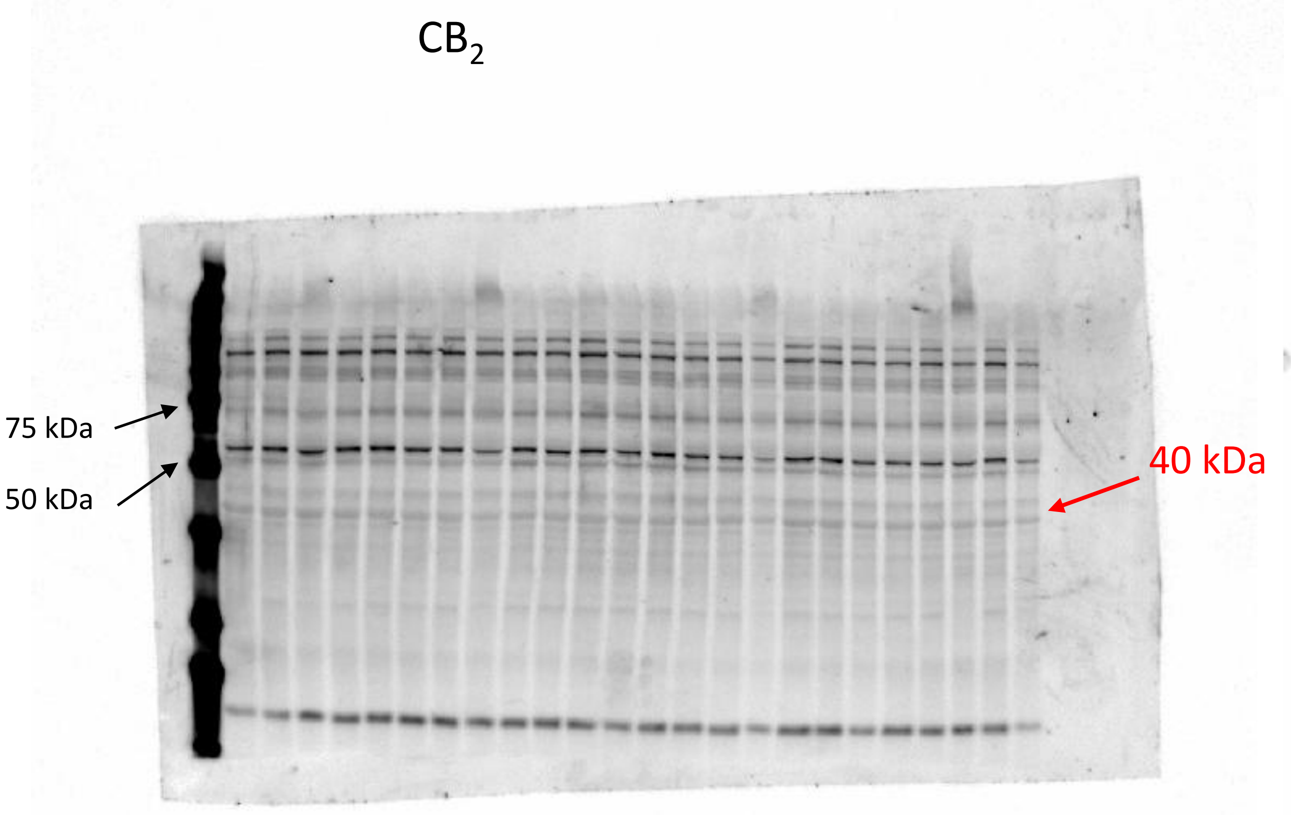

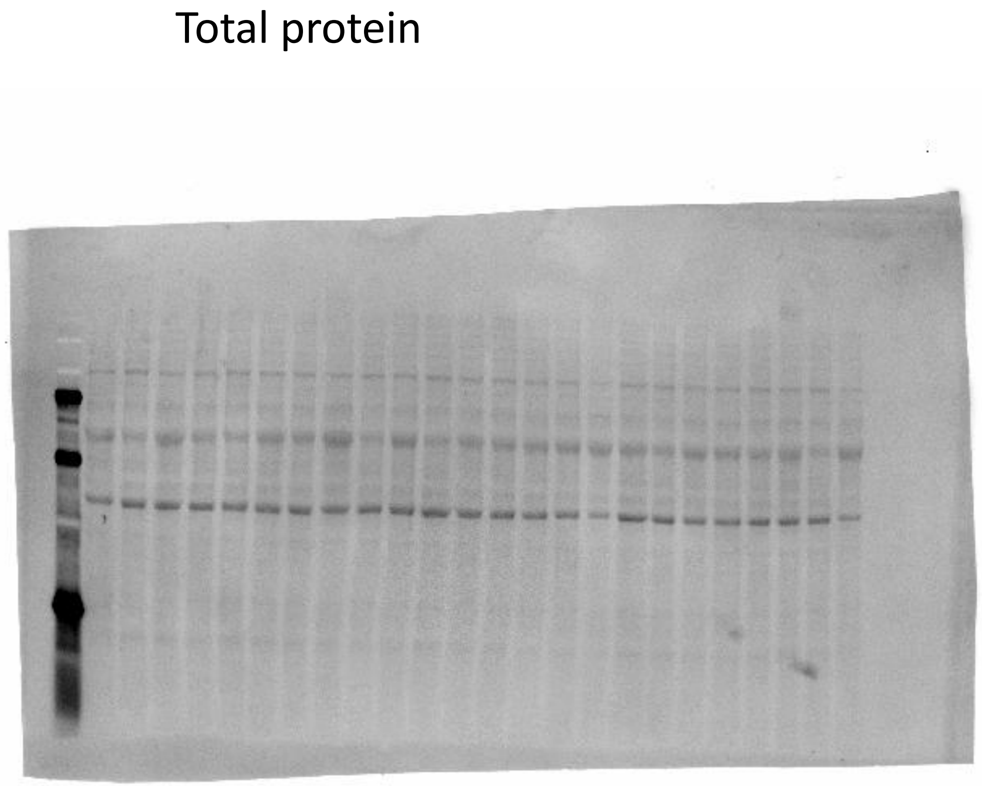

Loading order in all proteins: Control, INS, CBG+INS, PA+INS, PA+CBG+INS

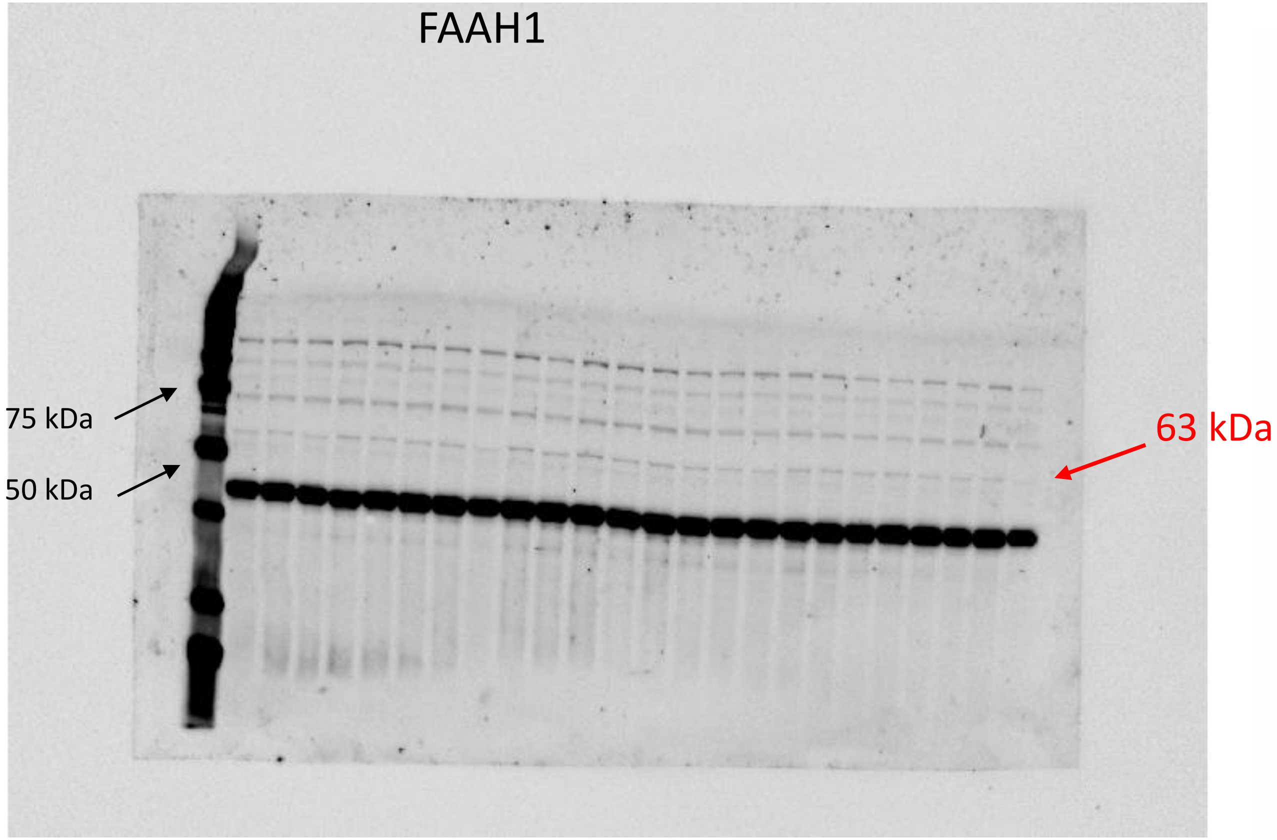

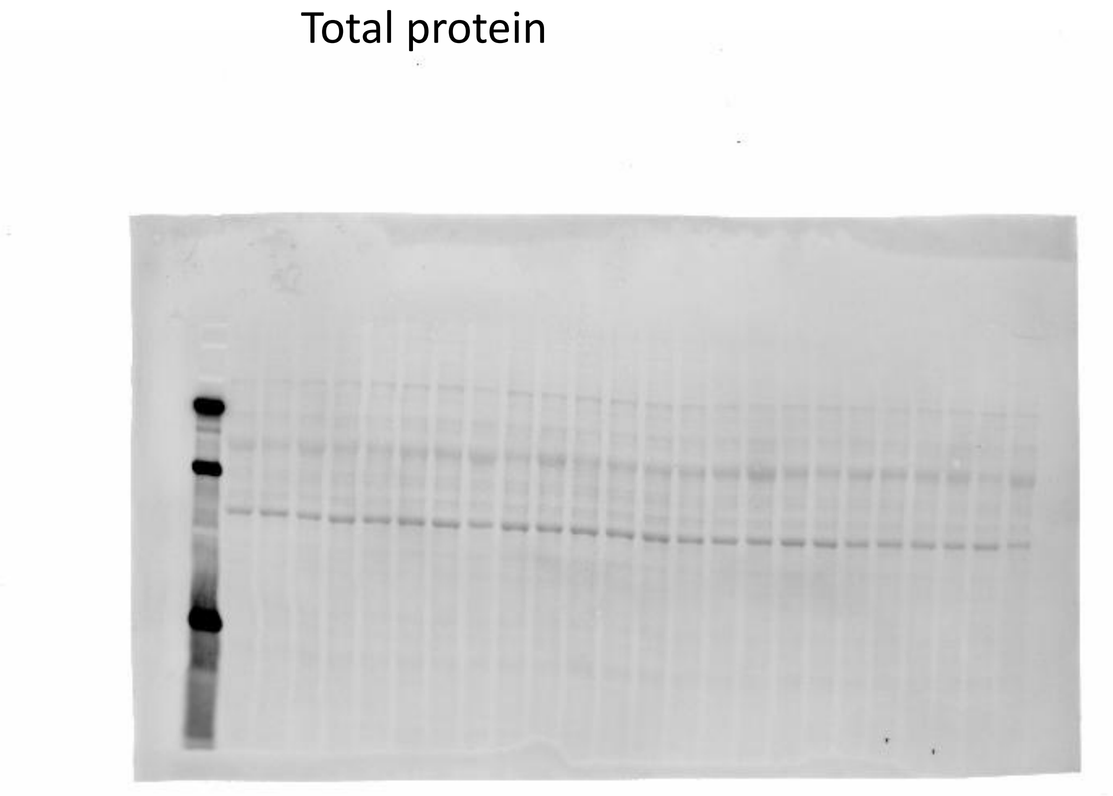

Loading order in all proteins: Control, INS, CBG+INS, PA+INS, PA+CBG+INS

MAGL

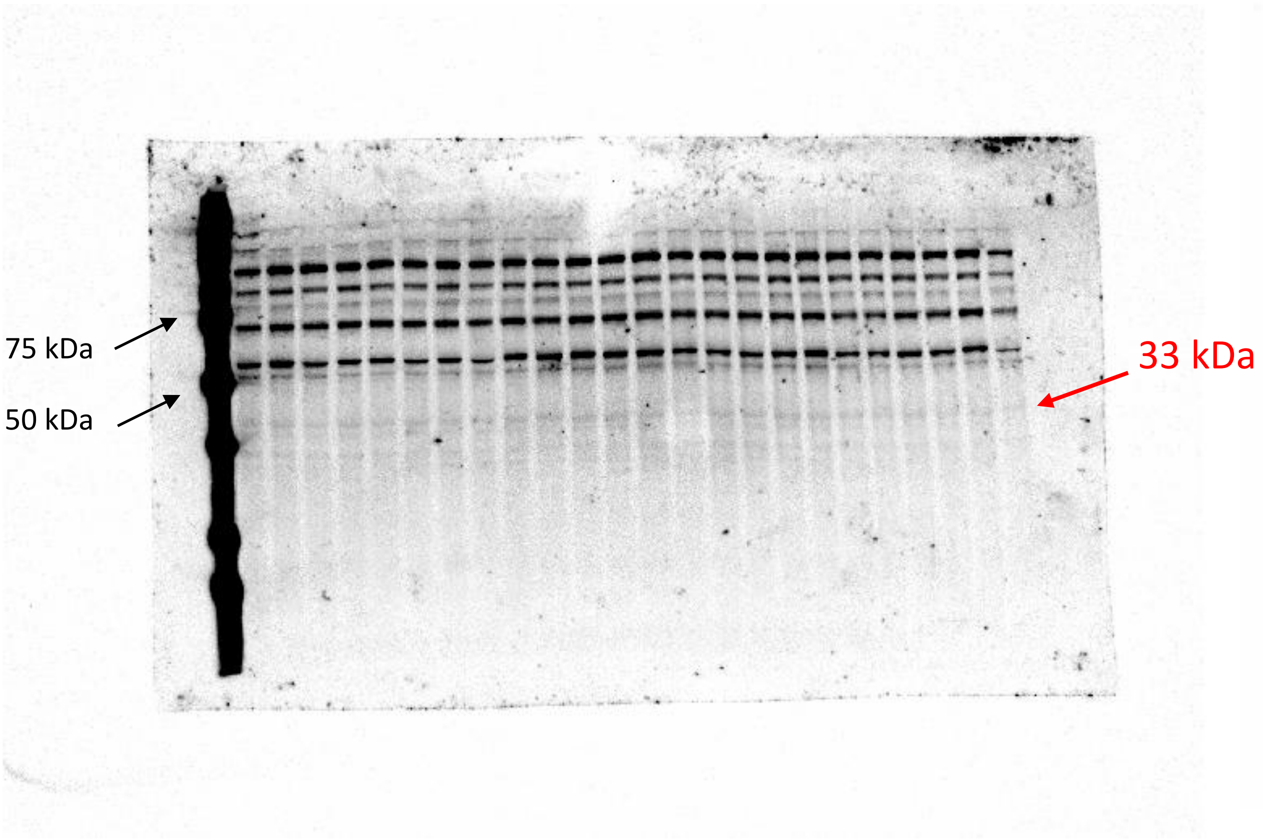

Total protein

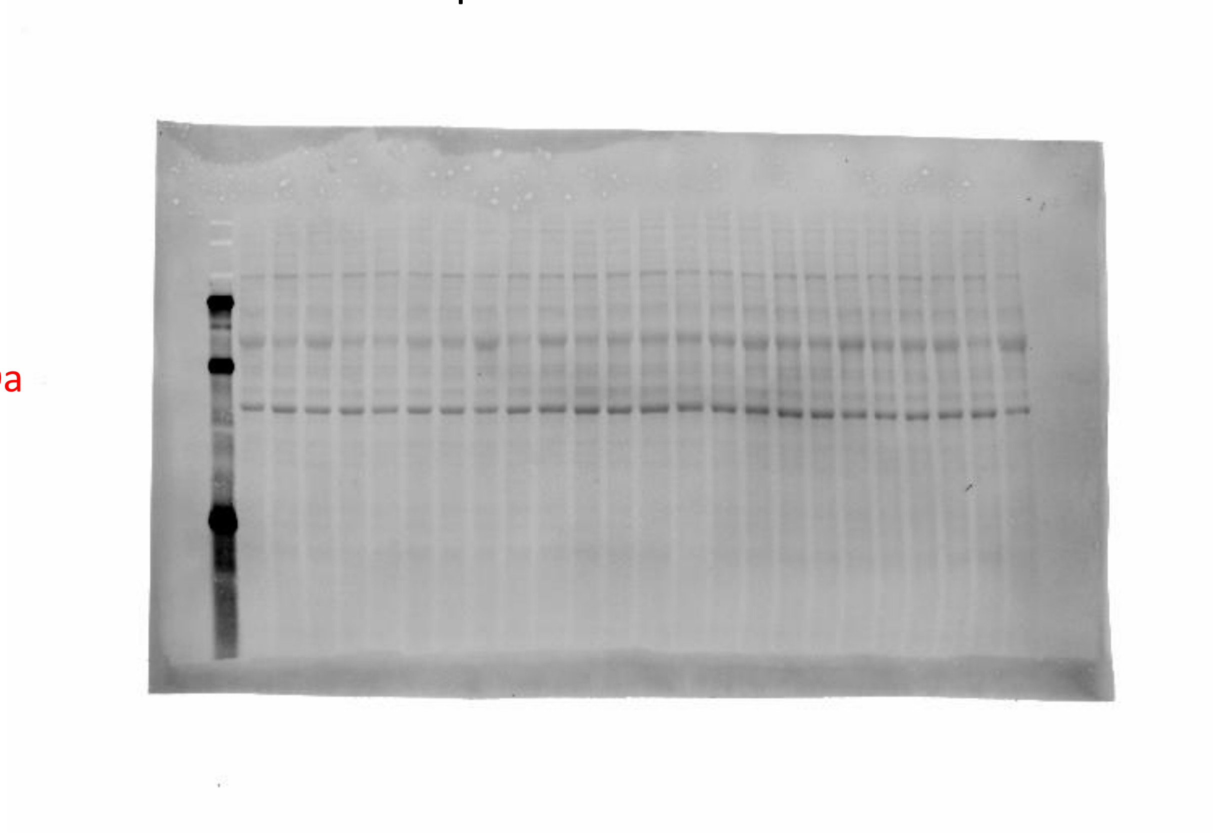

Loading order in all proteins: Control, INS, CBG+INS, PA+INS, PA+CBG+INS
